# Supplementary material for: A general esterolysis strategy for upcycling waste polyesters into high-value esters
Source: Nat Commun. 2025 Aug 12;16:7441. doi: 10.1038/s41467-025-62916-2 (PMC12343957; doi:10.1038/s41467-025-62916-2)
Supplement: Supplementary file 1 — Supplementary Information [file 41467_2025_62916_MOESM1_ESM.pdf]

Supplementary Information

A General Esterolysis Strategy for Upcycling Waste Polyesters into High-Value Esters

Minghao Zhang[1,2], Yunkai Yu[1,2], Buxing Han[3], Qingqing Mei[*1,2,4]

[1]State Key Laboratory of Soil Pollution Control and Safety, Zhejiang University, Hangzhou 310058, China.

[2]Institute of Environment Science and Technology, College of Environmental and Resource Sciences, Zhejiang University, Hangzhou, 310058, Zhejiang, China.

[3]Institute of Chemistry, Chinese Academy of Sciences, Zhongguancun North First Street 2, Beijing, China.

[4]Innovation Center of Yangtze River Delta, Zhejiang University, Zhejiang 311400, China.

*Corresponding author: Qingqing Mei

Address: 866 Yuhangtang Road, Hangzhou 310058, China.

E-mail address: meiqq@zju.edu.cn

Table of Contents

1. General Information 3
2. Catalytic System Exploration 6
3. The study on PET depolymerization process and products 13
4. Study on the kinetics of PET esterolysis 21
5. Study on the causes of initial esterolysis of PET 22
6. Characterization of possible hydrogen bond formation during PET esterolysis 30
7. Study on the influence of functional groups on the benzene ring of carboxylic acids 33
8. The possible reaction mechanism of DMC-involved PET esterolysis 34
9. ^{1}H NMR and ^{13}C NMR characterization date of all compounds 37
10. ^{1}H NMR and ^{13}C NMR spectra of all compounds 41
11. LC-MS spectra of part EG-derived product 60
References 72

1. General Information

1.1 Materials：

Analytical grade solvents and commercially available reagents were purchased from commercial sources and used directly without further purification unless otherwise stated. PET Powder (0.075 mm, Made from Bottle-grade PET Chip, CR Chem-MAT CR-8863), poly(ethylene glycol succinate) (98%, Bide Pharmatech Ltd.), poly(ethylene adipate) (Mw: ~1000, Aladdin), poly(butylene terephthalate) (Alfa), poly(ethylene 2,5-furandicarboxylate) (Alfa), dimethyl carbonate (>99%, Sinopharm), diethyl carbonate (99%, Sigma-Aldrich), dibutyl carbonate (97%, Acmec), dimethyl malonate (98%, Adamas), dimethyl 2-methylpropanedioate (95%, Solarbio), dimethyl decarbonate (98%, Solarbio), methyl phenyl carbonate (98%, Macklin), ethylene carbonate (99%, Meryer), terephthalic acid (99%, Innochem), 2-hydroxyethyl methyl terephthalate (99%, Shang hai D&B), bis(2-hydroxyethyl) terephthalate (99%, Shang hai D&B), ethane-1,2-diyl dibenzoate methyl benzoate (98%, Leyan), 4-(methoxycarbonyl)benzoic acid (98%, Bidepharm), benzoic acid (98%, Aladdin), 4-methylbenzoic acid (98%, TCI), methyl 3-methylbenzoate (99%, Shang hai D&B), 2-methylbenzoic acid (99%, Shang hai D&B), 4-(*tert*-butyl)benzoic acid (99%, Shang hai D&B), 4-methoxybenzoic acid (99%, Shang hai D&B), 4-phenoxybenzoic acid (98%, Shang hai D&B), 4-hydroxybenzoic acid (99%, Aladdin), 4-fluorobenzoic acid (98%, Adamas), 4-chlorobenzoic acid (99%, Aladdin), 4-bromobenzoic acid (99%, Bidepharm), 4-acetylbenzoic acid (96%, Aladdin), cinnamic acid (98%, Shang hai D&B), 5-bromofuran-2-carboxylic acid (98%, Shang hai D&B), 5-methylthiophene-2-carboxylic acid (98%, Shang hai D&B), isonicotinic acid (99%, Bidepharm), 1-naphthoic acid (97%, Heowns), 2-naphthoic acid (98%, Shang hai D&B), anthracene-9-carboxylic acid (96%, Leyan), $3a^1$,9-dihydropyrene-4-carboxylic acid (97%, Damas-beta), [EMIm][Cl] (97%, Aladdin), [EMIm][OAc] (98%, Meryer), [EMIm][Br] (98%, Meryer), [EMIm][I] (97%, Rhawn), [EMIm][$MeSO_3$] (98%, Meryer), [EMIm][BF_4] (98%, Meryer), [BMIm][OAc] (98%, Meryer), dichloromethane (AR, Shanghai Linfeng Chemical Reagent CO. LTD), ethylene glycol (AR, Sinopharm), MeOH (>99%, J&K Scientific), $CDCl_3$ (99%, Sigma-Aldrich), DMSO-*d*6 (99%, Adamas), and deionized water were used herein.

1.2 Characterizations：

^{1}H NMR and ^{13}C NMR spectra were recorded at room temperature using a Bruker Avance-600 instruments (^{1}H NMR at 600 MHz and ^{13}C NMR at 151 MHz), NMR spectra of all products were reported in ppm with reference to solvent signals [^{1}H NMR: CD(H)Cl_3 (7.26 ppm), ^{13}C NMR: CD(H)Cl_3 (77.00 ppm)]. Signal patterns are indicated as s, singlet; d, doublet; dd, doublets of doublet; t, triplet, and m, multiple. The detailed NMR characterizations and spectra of [EMIm][OAc], product DMT and mixture of [EMIm][OAc] with reactants (MB, DMC, and EC) are provided in Supplementary materials. The product yield was analyzed by Gas chromatography (GC): Agilent 8860 with Agilent J&W HP-5 Polysiloxane GC Column and Gas

chromatography-mass spectrometry (GC-MS): Agilent 7890A/5975C GC/MSD with Agilent J&W HP-5 Polysiloxane GC Column. Liquid Chromatograph Mass Spectrometer (LC-MS): Waters ACQUITY Premier/Xevo G2-XS was used in chromatographic experiments. The mobile phases were water (A) and acetonitrile (B). The linear gradient pro-grams were as follows, 0 min 5%B; 4 min 95%B; 7 min 95%B; 10 min 5%B; Sample injection volume, 1μL; Column oven temperature, 40 °C; Flow rate, 0.4mL min^{-1}. And the FT-IR spectra were recorded at a spectral resolution of 2 cm^{-1} using a Nicolet iS20 FT-IR spectrometer (Thermo Fisher Scientific). The surface morphology of PET bottles and their treated counterparts were characterized using cold-field scanning electron microscopy (SEM, Hitachi, SU8010, Japan).

1.3 Experimental

PET powder (1 mmol), [EMIm][OAc] (20 mol%), DMC (4 mmol), and NMP (3 mL) were combined in a polytetrafluoroethylene rotor (25 mL), which was then hermetically sealed within a s tainless-steel reactor. The reactor was stirred at 140°C for 4 hours with a rotational speed of 500 r/min on the heating plate. Following the completion of the reaction, the system was quenched by transferring the reactor into an ice bath. Subsequently, the resulting reaction solution was diluted with 10 mL of dichloromethane. The liquid products were subjected to analysis via gas chromatography (GC), using mesitylene (0.5 mmol) as an internal standard. The yields of ethylene carbonate (EC) and dimethyl terephthalate (DMT) were calculated using the following equation:

$$\text{(1) Theoretically produced product amount (mol)} = \frac{\text{Quality of PET material (g)}}{\text{0.192 g/mol}}$$

$$\text{(2) DMT Yield (\%)}=\frac{\text{DMT amount quantified by GC(mol)}}{\text{Theoretically Produced DMT amount (mol)}}\times 100\%$$

The ^{1}HNMR yield of EC and DMT was determined as follows: After completion of the reaction, the liquid products were analyzed by ^{1}HNMR using mesitylene (1 mmol) as an internal standard. The yield of EC and DMT were calculated using the following equation:

$$\text{(1) Theoretically produced product amount (mol)} = \frac{\text{Quality of PET material (g)}}{\text{0.192 g/mol}}$$

$$\text{(2) DMT Yield (\%)}=\frac{\text{DMT amount quantified by H NMR (mol)}}{\text{Theoretically Produced DMT amount (mol)}}\times 100\%$$

$$\text{(3) EC Yield (\%)}=\frac{\text{EC amount quantified by H NMR (mol)}}{\text{Theoretically Produced EC amount (mol)}}\times 100\%$$

The depolymerization process was extended to various other polyesters, such as poly(ethylene succinate) (PES), poly(ethylene adipate) (PEA), polyethylene 2,5-furandicarboxylate (PEF), and polylactic acid (PLA), employing the same method used for the depolymerization of PET.

Additionally, a recycling experiment was executed to validate the purity of the reclaimed value-added product DMT. The resultant reaction solution underwent an initial wash with deionized water to separate water-soluble compounds ([EMIm][OAc], DMC, and EC) from crude DMT. The resulting crude DMT was

then dried in an oven at 80°C overnight. The purity assessment of the product was conducted through ^{1}H NMR spectroscopy. Higher purity DMT can be further obtained through a recrystallization step using methanol. The DMT separation process and product purity are described in Fig. S3 and Fig. S4-S5. The resulting aqueous mixture may then be distilled, taking advantage of differing boiling points to facilitate subsequent separation processes.

2. Catalytic System Exploration

2.1. Comparison of this strategy with other methanolysis methods

Table S1. Methanolysis of PET with reported catalysts.

Entry	Catalysts	Co-sol.	MeOH:PET	Temp. (°C)	T (h)	Conv. (%)	Yield (%)	Ref.
1	CO_2	-	n(MeOH:PET) = 36:1	220	0.83	100	79.1	1
2	AlP	Toluene	n(MeOH:PET) = 76:1	200	2	-	88.5	2
3	$Pb(AC)_2$+ $Zn(AC)_2$	-	n(MeOH:PET) = 16.8:1	120	2	97.8	97.8	3
4	ZnO nanodispersions	-	n(MeOH:PET) = 36:1	170	0.25	97	95	4
5	K_2CO_3	DCM	n(MeOH:PET) = 50:1	25	24	100	93.1	5
6	Orange peel ash		n(MeOH:PET) = 47.4:1	200	1	-	79	6
7	OPA@Fe_2O_3	-	n(MeOH:PET) = 49:1	200	1	100	83	7
8	LiOMe	DMC	n(MeOH:PET) = 23.4:1	65	5	-	91	8
9	Bamboo leaf ash	-	n(MeOH:PET) = 49.4:1	200	2	100	78	9
10	$Ti_{0.5}Si_{0.5}O_2$	-	n(MeOH:PET) = 46.8:1	160	2	100	98.2	10
11	Calcined sodium silicate	-	n(MeOH:PET) = 30:1	200	0.5	100	95	11
12	MgO/NaY	-	n(MeOH:PET) = 36:1	200	0.5	99	91	12
13	ChCl/$Zn(OAc)_2$	MeCN	n(MeOH:PET) = 15:1	170	1	100	90.1	13
14	[HDBU][Im]	-	n(MeOH:PET) = 4.8:1	140	3	100	75	14
15	PIL-Zn^{2+}	-	n(MeOH:PET) = 24:1	170	1	100	90.3	15
16	$[HO_3S\text{-}(CH_2)_3\text{-}NEt_3]Cl[ZnCl_2]_{0.67}$	-	n(MeOH:PET) = 24:1	195	0.5	-	78.4	16
17	DBN/Phenol	-	n(MeOH:PET) = 18:1	130	1	100	95.3	17
18	[BMIm][OAc]	-	n(MeOH:PET) = 24:1	150	4	88.5	41.7	18
19	[EMIm][OAc]	NMP	n(DMC:PET) = 4:1	140	4	100	99	This work

The products derived from the strategy we proposed are analogous to those obtained from the methanolysis of PET, as both contain DMT. We further investigated the relevant literature on PET methanolysis in detail (Table S1). We found that in most approaches, the reaction system temperature is ≥140°C, and the DMT yield is typically below 95%. Notably, PET methanolysis catalyzed by ionic liquids requires relatively high temperatures; when the temperature is below 150 °C, reaction efficiency and product selectivity decrease significantly. In contrast, DMC enables transesterification reactions to occur at lower temperatures, which decreases energy requirements and allows for milder operational conditions. Additionally, DMC is considered a green regent due to its low toxicity and minimal environmental impact, further eliminating the need for external alcohol solvents and thereby reducing both raw material costs and separation challenges. During reaction process, DMC is not only as a methylating agent, but also an EG capturing reagent. Our developed esterolysis process of PET using DMC exhibited superior reaction selectivity, achieving complete PET conversion and simultaneously yielding two high-value esters, DMT and EC, at temperatures ≤140°C without the need for strong bases or acids.

2.2. The different structures of ILs

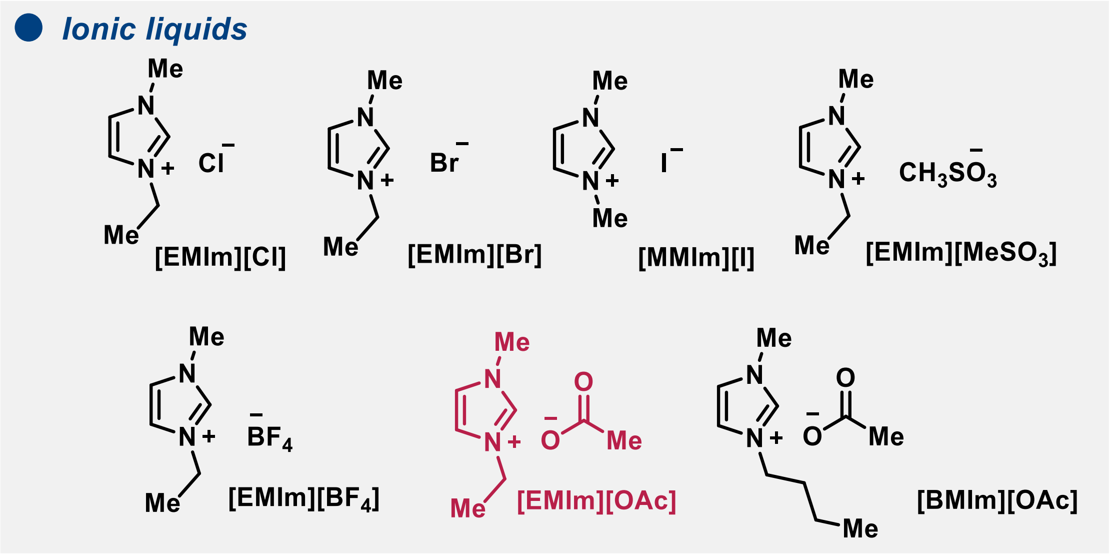

Fig. S1. The different structures of ILs used in this study.

2.3. Optimization of reaction solvent

Table S2. Comparison of reaction solvent

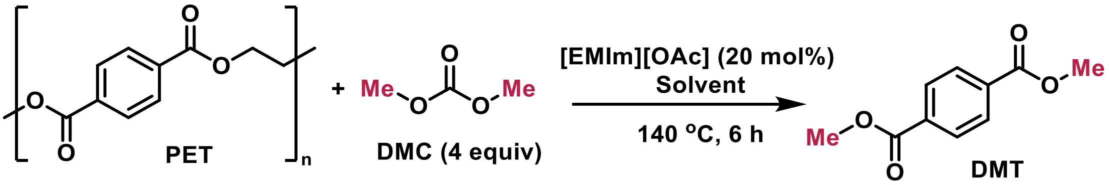

Entry	Catalyst	loading	Temperature	Solvent	DMT Yield (%)[b]
1	[Emim][OAc]	20 mol%	140 °C	DMSO	89
2	[Emim][OAc]	20 mol%	140 °C	NMP	99
4	[Emim][OAc]	20 mol%	140 °C	DMF	96
5	[Emim][OAc]	20 mol%	140 °C	Benzotrifluoride	94
6	[Emim][OAc]	20 mol%	140 °C	1,4-Dioxane	92
7	[Emim][OAc]	20 mol%	140 °C	MeCN	91
8	[Emim][OAc]	20 mol%	140 °C	Toluene	91
10	[Emim][OAc]	20 mol%	140 °C	Cyclohexane	85
11	[Emim][OAc]	20 mol%	140 °C	THF	80
12	[Emim][OAc]	20 mol%	140 °C	Acetone	92
13	[Emim][OAc]	20 mol%	140 °C	Chlorobenzene	9
14	[Emim][OAc]	20 mol%	140 °C	Bromobenzene	-
15	[Emim][OAc]	20 mol%	140 °C	Chloroform	-
16	[Emim][OAc]	20 mol%	140 °C	DMC	99

Catalytic system and reaction conditions exploration. [a] Standard reaction conditions: PET (1 mmol), DMC (4 mmol), [Emim][OAc] (20 mol%), and solvent (3 mL) at 140 °C for 4 h. [b] Yields were mesitylene by GC analysis with mesitylene as internal standard.

2.4. Optimization of catalyst loading

Table S3. Comparison of catalyst loading

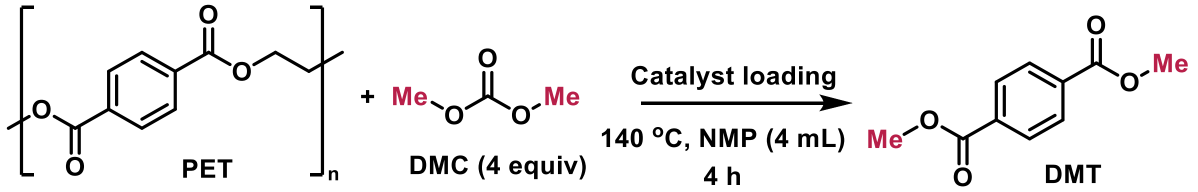

Entry	Catalyst	Catalyst loading	Temperature	Solvent	DMT Yield (%)[b]
1	[Emim][OAc]	5 mol%	140 °C	NMP	76
2	[Emim][OAc]	10 mol%	140 °C	NMP	79
3	[Emim][OAc]	15 mol%	140 °C	NMP	93
4	[Emim][OAc]	20 mol%	140 °C	NMP	99
5	[Emim][OAc]	25 mol%	140 °C	NMP	99

Catalytic system and reaction conditions exploration. [a] Standard reaction conditions: PET (1 mmol), DMC (4 mmol), [Emim][OAc] (20 mol%), and solvent (3 mL) at 140 °C for 4 h. [b] Yields were determined by GC analysis with mesitylene as internal standard.

2.5. Optimization of methylation reagent dosage by GC analysis

Table S4. Comparison of methylation reagent dosage.

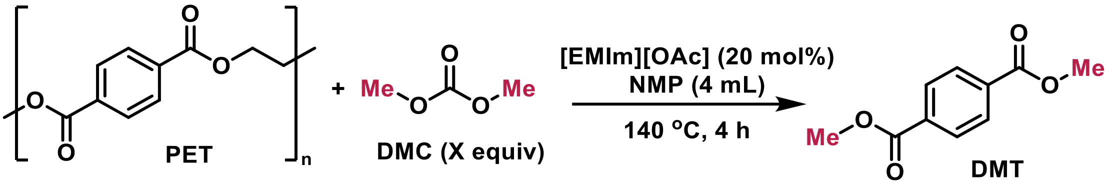

Entry	Catalyst	loading	Temperature	Solvent	DMC (mmol)	DMT Yield (%)[b]
1	[Emim][OAc]	20 mol%	140 °C	NMP	1	39
2	[Emim][OAc]	20 mol%	140 °C	NMP	2	57
3	[Emim][OAc]	20 mol%	140 °C	NMP	3	94
4	[Emim][OAc]	20 mol%	140 °C	NMP	4	99
5	[Emim][OAc]	20 mol%	140 °C	NMP	5	99

Catalytic system and reaction conditions exploration. [a] Standard reaction conditions: PET (1 mmol), DMC (X mmol), [Emim][OAc] (20 mol%), and NMP (3 mL) at 140°C for 4 h. [b] Yields were determined by GC analysis with mesitylene as internal standard.

2.6. Optimization of reaction conditions by ^{1}H NMR analysis

Table S5. Comparison of IL-catalyzed PET esterolysis condition with DMC*[a]*

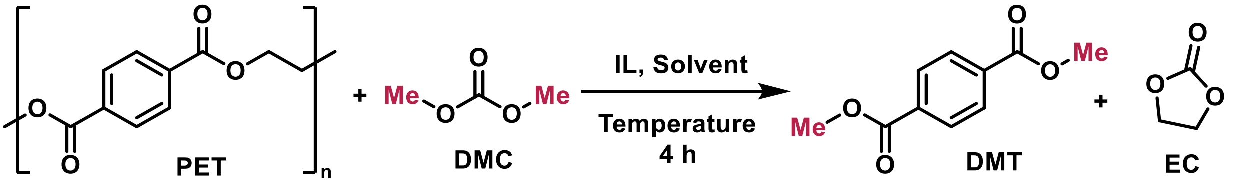

Entry	Catalyst	loading	Temperature	Solvent	DMT Yield (%)[b]	EC Yield (%)[b]
1	-	-	130 °C	DMSO	0	0
2	[EMIm][Cl]	20 mol%	130 °C	DMSO	18	20
4	[EMIm][Br]	20 mol%	130 °C	DMSO	-	-
5	[EMIm][I]	20 mol%	130 °C	DMSO	-	-
6	[EMIm][$MeSO_3$]	20 mol%	130 °C	DMSO	-	-
7	[EMIm][BF_4]	20 mol%	130 °C	DMSO	-	-
8	[EMIm][OAc]	20 mol%	130 °C	DMSO	73	62
9	[BMIm][OAc]	20 mol%	130 °C	DMSO	59	51
10	[EMIm][OAc]	20 mol%	120 °C	DMSO	32	28
11	[EMIm][OAc]	20 mol%	140 °C	DMSO	90	76
12	[EMIm][OAc]	20 mol%	150 °C	DMSO	94	65
13	[EMIm][OAc]	20 mol%	140 °C	NMP	99 (99)[c]	90
14	[EMIm][OAc]	20 mol%	140 °C	DMF	90	73
15	[EMIm][OAc]	20 mol%	140 °C	Toluene	93	68
16	[EMIm][OAc]	20 mol%	140 °C	THF	85	70
17	[EMIm][OAc]	15 mol%	140 °C	NMP	90	75
18	[EMIm][OAc]	10 mol%	140 °C	NMP	83	71

Catalytic system and reaction conditions exploration. [a] Standard reaction conditions: PET (1 mmol), DMC (4 mmol), catalyst (20 mol%), and solvent (3 mL) at 140 °C for 4 h. [b] Yields were determined by ^{1}H NMR analysis with mesitylene as internal standard. [c] Yields were determined by GC analysis with mesitylene as internal standard.

The ILs-catalyzed PET esterolysis system involving PET depolymerization and EG transesterification with DMC was investigated. Initially, no PET depolymerization occurred at 130 °C in the absence of ILs catalyst (Table S4, entry 1). To investigate the catalytic effect of cations and anions on PET esterolysis, a variety of imidazolium-based ILs were examined (Fig. S1). It was observed that only the basic [EMIm][OAc] exhibited significant catalytic activity in DMC-involved PET esterolysis, yielding DMT and EC in 73% and 62% (^{1}H NMR yield), respectively. In contrast, other imidazolium-based ILs such as [EMIm][X], [EMIm][$MeSO_3$], or [EMIm][BF_4] were ineffective in catalyzing the reaction combined with DMSO as solvent, underscoring the significant role of $[OAc]^-$ in this process. (Table S5, entries 2-8). These results can be attributed to the excellent hydrogen bond formation ability and basic strength of the anions.[19-21] Additionally, the [BMIm][OAc] catalyzed PET esterolysis led to a decrease in both DMT and EC yields. The results demonstrated that $[EMIm]^+$ has better catalytic activity than $[BMIm]^+$, indicating an inverse relationship

between the H-bond donor capacity and the cationic alkyl chain length of $[C_nMIm]^+$.[21] Previous studies have emphasized the effectiveness of *N*-methylpyrrolidone (NMP) as a solvent and promoter in transesterification reactions.[22,23] NMP could potentially serve as an effective facilitator in PET esterolysis. The use of NMP as a solvent led to the rapid dissolution of PET (SEM analysis, Fig. S2) and a significant enhancement in the production of both DMT and EC, achieving yields of 99% and 90%, respectively. Subsequent experiments involving adjustments in solvents or catalyst loading did not result in further improvements in product yield (Table S5, entries 13-18 and Table S1-S2). Decreasing the amount of DMC led to an incomplete esterolysis reaction, while using four equivalents or higher amounts of DMC resulted in the complete esterolysis of PET to DMT with 99% yield (Table S4). The produced DMT can be easily separated and purified, as shown in Fig. S4. Therefore, this PET esterolysis strategy presents a novel and effective pathway for transforming PET into the valuable product DMT without additional alcohol sources.

3. The study on PET depolymerization process and products

3.1. SEM characterization of the change process of plastic bottles in NMP.

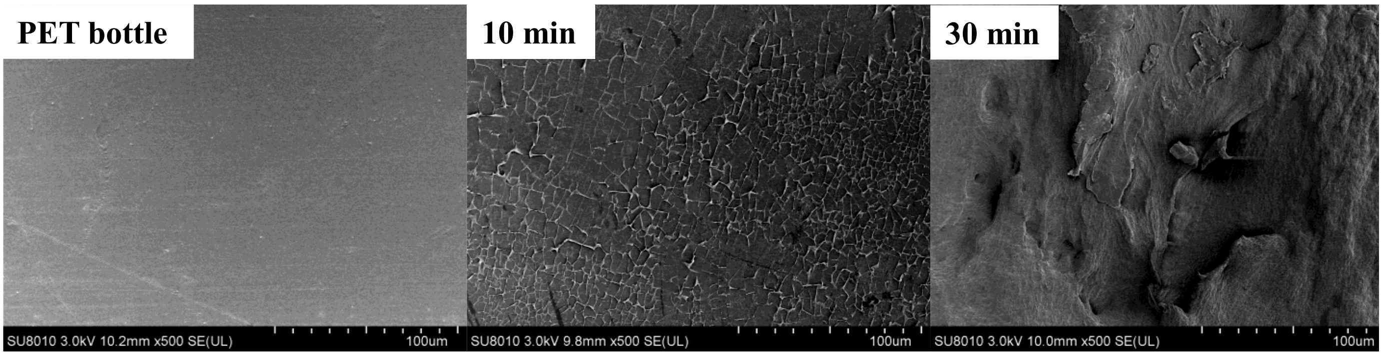

Fig. S2. SEM images of various PET bottles treated with NMP for different durations.

Scanning Electron Microscopy (SEM) analysis on raw PET bottle and their treated counterparts at 140 °C for different times (10min and 30min) were performed and the results were shown in Fig. S2. The SEM image revealed that the raw PET bottle samples exhibited a smooth and flat structure. After being treated with NMP, the PET bottle displayed a noticeable transformation: the smooth and flat structure disappeared and transformed into an uneven structure with cracks, and this phenomenon gradually became obvious as time went by. This structural change can be attributed to the good solubility of PET in NMP with increasing temperature, facilitating the PET depolymerization.

3.2. Analysis of PET esterification products by ^1H NMR

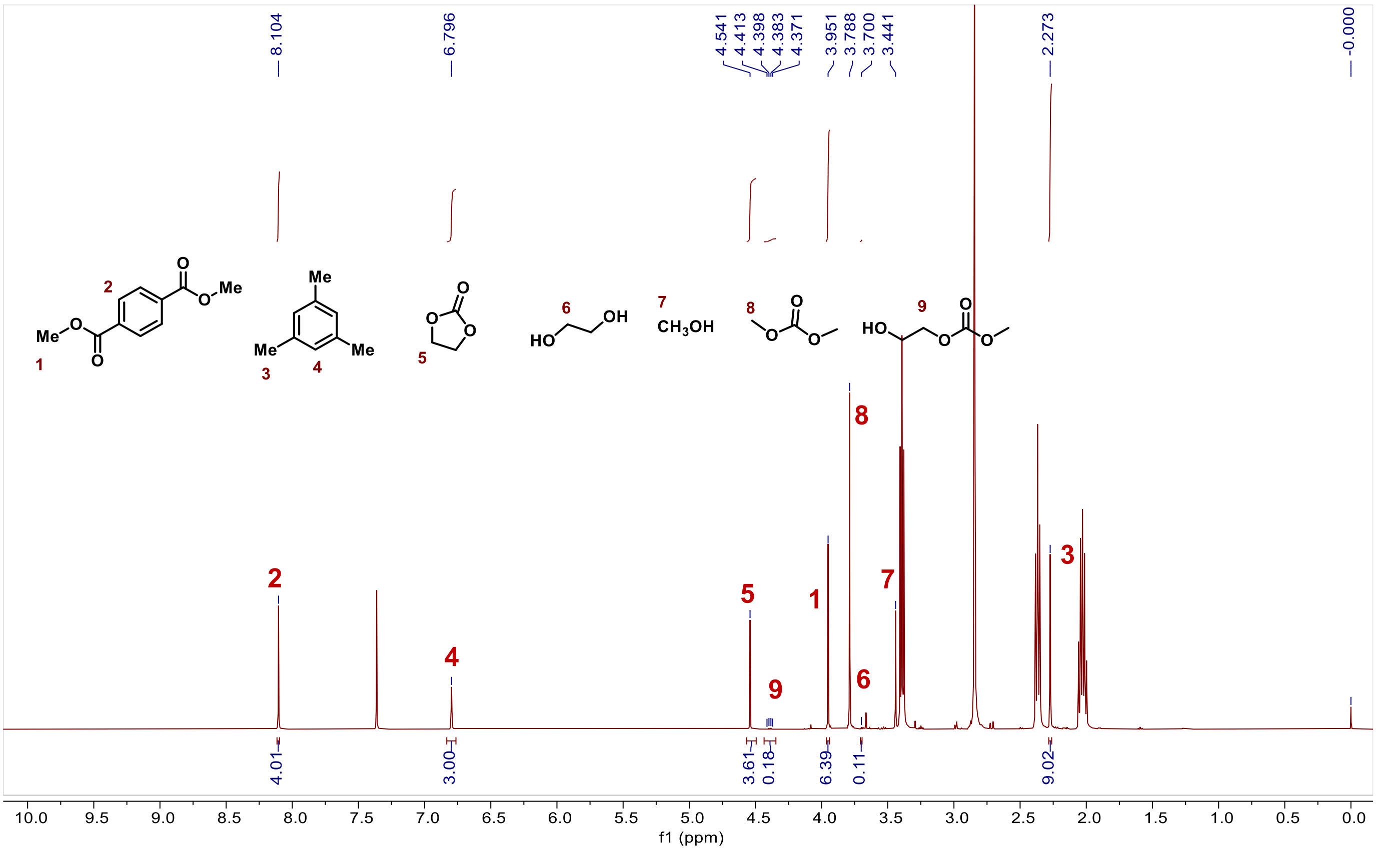

Fig. S3. ^1H NMR spectrum (600 MHz, $CDCl_3$, 298 K) of reaction mixture with mesitylene (1 mmol) as internal standard.

This ^1H NMR spectrum, as described in Fig. S3, indicated that PET was fully converted into DMT and EC with a yield of 99% and 90%, respectively. The product mixture contained unreacted EG (~3-4%) and 2-hydroxyethyl methyl carbonate (~5%), as well as EC, with no detectable formation of the by-product 1,2-dimethoxycarbonyloxyethane.

3.3. Simple product separation process after PET esterolysis

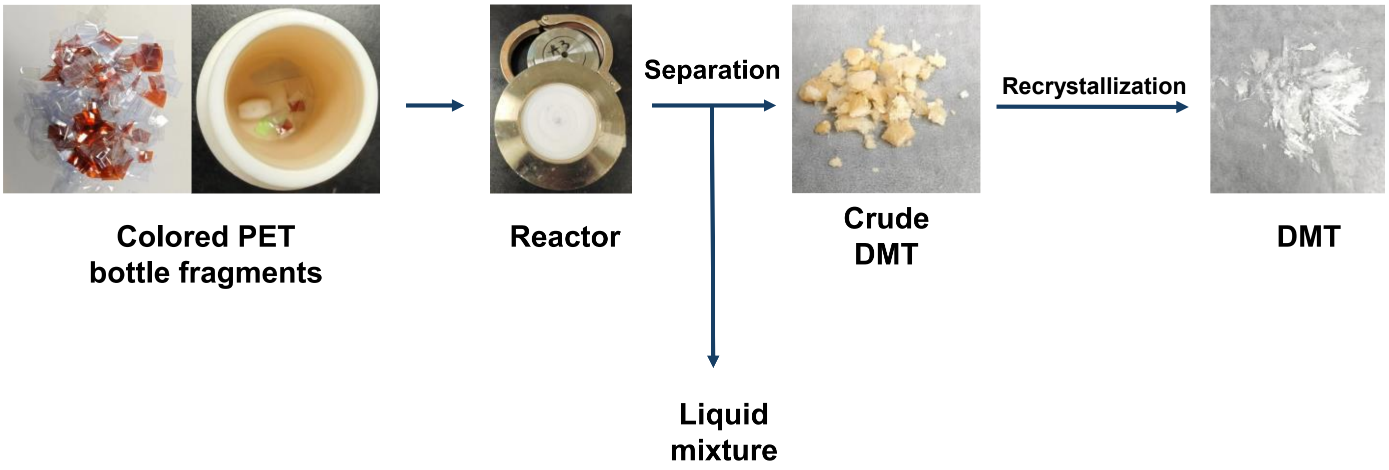

Fig. S4. Simple separation of product DMT after PET esterolysis.

The simple separation process of product DMT was illustrated in Fig. S4. Following the completion of the depolymerization reaction, the resultant solution was subjected to an initial wash with deionized water to separate water-soluble compounds ([EMIm][OAc], DMC, and EC) and water-insoluble product (DMT). Crude DMT was then obtained through solid-liquid separation. Highly pure, transparent DMT crystals were subsequently harvested via recrystallization using methanol as the solvent.

168 3.4 Simple solvent and product separation after 50 g-Scale PET esterolysis

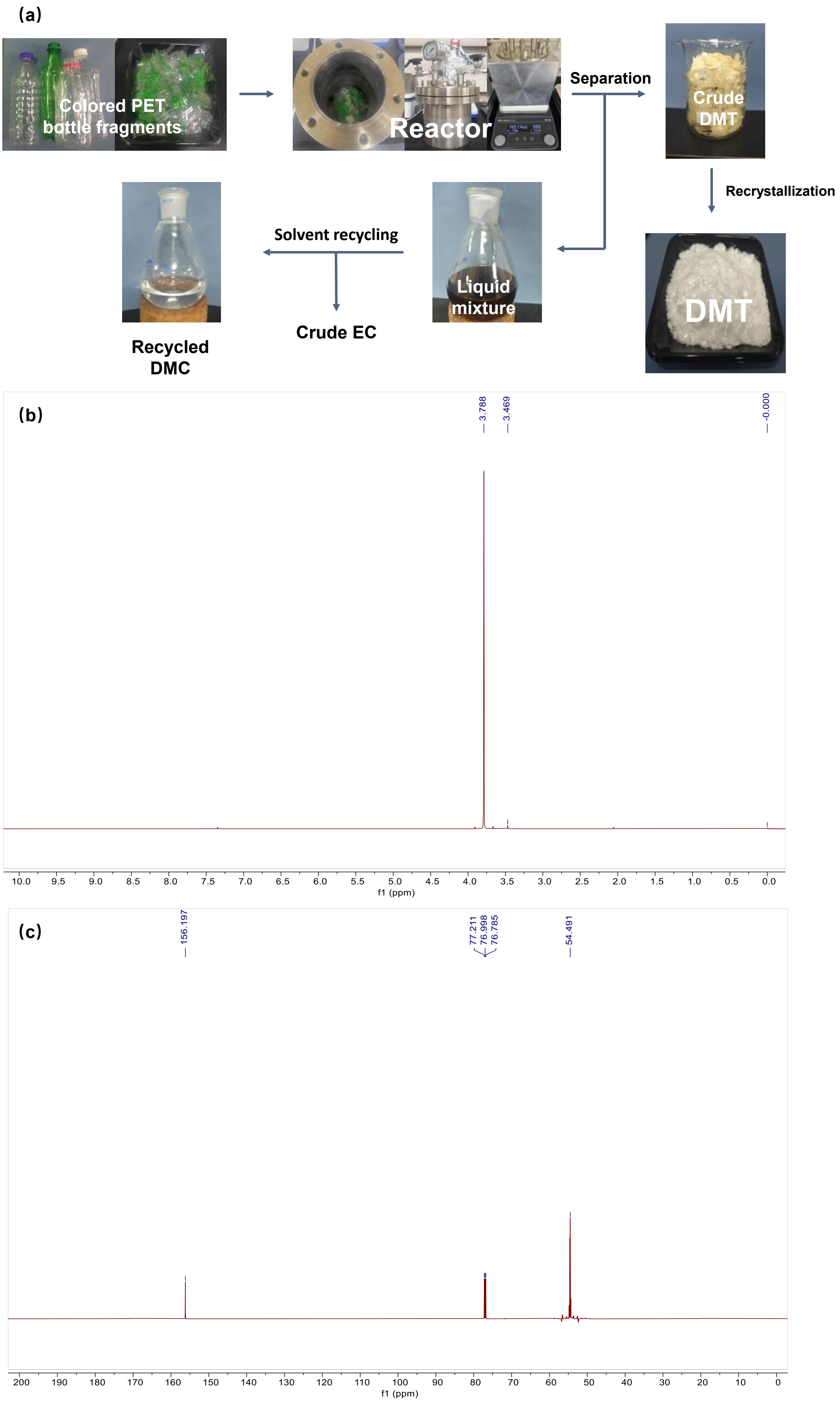

Fig. S5. Solvent recovery experiments. (a) Separation of solvent and recovery of products; (b) ^{1}H NMR spectrum (600 MHz, $CDCl_3$, 298 K) of the recycled DMC; (c) ^{13}C NMR spectrum (600 MHz, $CDCl_3$, 298 K) of the recycled DMC.

To validate the effectiveness of this strategy, we conducted a scale-up experiment on a 50 g scale using PET bottle flakes, with DMC serving as both the solvent and methanol source, without the need for additional methanol. As shown in Fig. S5a, the post-reaction mixture could be readily separated via solid–liquid separation, yielding a liquid product phase and crude DMT. The crude DMT was purified by recrystallization to afford high-purity DMT (98%), while the liquid phase was efficiently recycled by rotary evaporation to recover DMC, leaving only trace amounts of methanol (Fig. S5b and 5c).

3.5. ^{1}H NMR characterization of obtained DMT

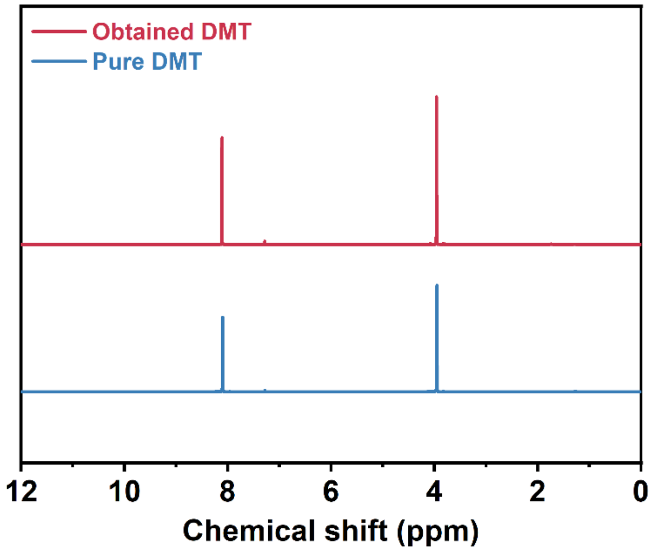

Fig. S6. ^{1}H NMR (600 MHz, $CDCl_3$, 298 K) patterns of the obtained DMT of gram-scale experiment and the commercial DMT (99.9%).

Fig. S6 illustrated the purity characterization of the DMT product, comparing it with data from a standard sample (commercially available DMT) using ^{1}H NMR analysis. The results from the ^{1}H NMR analysis indicated that the alkyl and aromatic peak position of the recycled DMT product aligns with that of the standard product, demonstrating that the recycled product exhibits high purity.

3.6. Condition optimization for poly(ionic liquid)s-catalyzed PET esterolysis

Table S6. PILs-catalyzed PET esterolysis condition with DMC

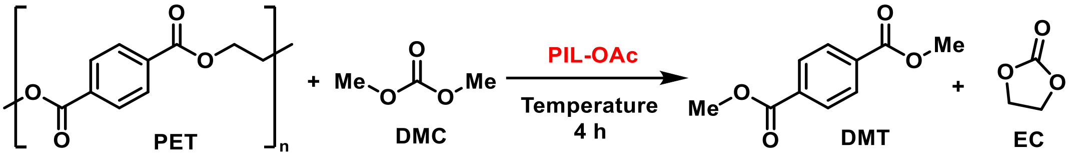

Entry	Catalyst	loading	Temperature	Solvent	DMT Yield (%)[b]	EC Yield (%)[b]
1	PIL-OAc	0.05g	150 °C	NMP	95	70
2	PIL-OAc	0.05g	160 °C	NMP	99	78
3	PIL-OAc	0.05g	170 °C	NMP	99	45
4	PIL-OAc	0.1g	160 °C	NMP	99	60
5	PIL-OAc	0.03g	160 °C	NMP	97	71
6	PIL-OAc	0.05g	160 °C	MeCN	99	81
7	PIL-OAc	0.05g	160 °C	Acetone	99	39
8	PIL-OAc	0.05g	160 °C	DMC	99	52

Catalytic system and reaction conditions exploration. [a] Standard reaction conditions: PET (0.192g, 1 mmol structural unit), DMC (4 mmol), and solvent (3 mL) at 160 °C for 4 h. [b] Yields were determined by ^{1}H NMR analysis with mesitylene as internal standard.

In response to the challenges associated with recycling [EMIm][OAc], we explored the use of poly(ionic liquid)s and successfully designed and synthesized an efficient catalyst, PIL-OAc, using *p*-divinylbenzene, zinc acetate, and 1-vinyl-3-methylimidazolium acetate as monomers, with AIBN as the initiator and MeCN as the solvent. To optimize the reaction conditions, a preliminary screening of catalyst loading, temperature, and solvent was performed. The results demonstrated that the synthesized PIL-OAc exhibited excellent catalytic activity, enabling complete depolymerization of PET. Notably, when NMP was used as the solvent at 160 °C, DMT and EC were obtained in yields of 99% and 78%, respectively. Notably, efficient PET conversion can be achieved using low-boiling-point solvents such as acetonitrile, yielding DMT and EC in 99% and 81%, respectively.

3.7. Recyclability test of PIL-OAc

Table S7. Recyclability test of PIL-OAc

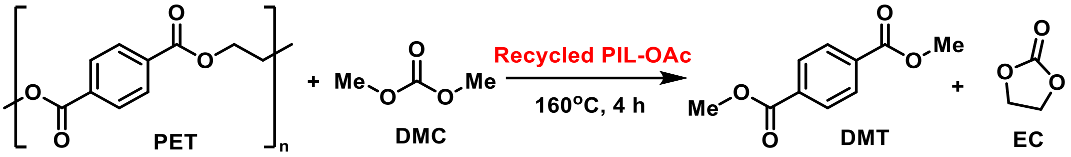

Cycle Number	Load.	Temp.	Sol.	DMT Yield (%)[b]	EC Yield (%)[b]
1	0.05g	160 °C	NMP	99	77
2	0.05g	160 °C	NMP	99	72
3	0.05g	160 °C	NMP	97	63

Catalytic system and reaction conditions exploration. [a] Standard reaction conditions: PET (0.192g, 1 mmol structural unit), DMC (4 mmol), and solvent (3 mL) at 160 °C for 4 h. [b] Yields were determined by ^{1}H NMR analysis with mesitylene as internal standard.

The PIL-OAc catalyst can be readily separated from the reaction system after the reaction. Even after three recycling cycles, it still enables efficient conversion of PET to DMT with a high yield of 97%. Although a slight decrease in DMT yield was observed upon extended recycling, the successful implementation of this process provides strong evidence supporting the recyclability of this catalytic system.

4. Study on the kinetics of PET esterolysis

4.1. Kinetic studies of the overall reaction

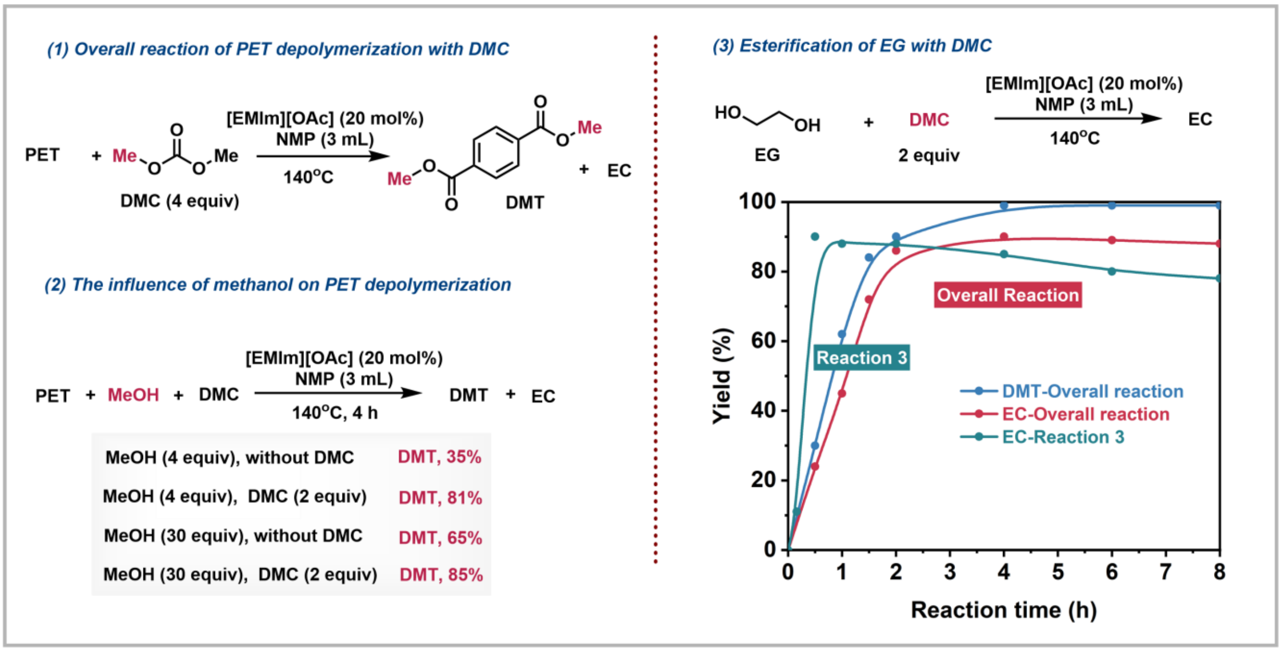

Fig. S7. Kinetic studies. Standard reaction conditions: PET (1 mmol), DMC (4 mmol), [EMIm][OAc] (20 mol%), and NMP (3 mL) at 140 °C for 4 h. Yields were determined by ^{1}H NMR analysis with mesitylene as internal standard.

To comprehend the reaction route, we performed controlled experiments and analyzed the reaction kinetics. To investigate the individual effects of methanol and EG transesterification on the reaction, we classified the reactions into three categories: (1) DMC-involved PET esterolysis (Reaction 1), (2) PET methanolysis (Reaction 2) and (3) EG transesterification with DMC (Reaction 3). When methanol was used as the methylation reagent instead of DMC, the yield of DMT was only 35% catalyzed by [EMIm][OAc] at 140°C for 4 hours. Even with an extremely excessive amount of 30 equivalents of methanol, the yield only reached 65% (Reaction 2). However, the incorporating 2 equivalents of DMC into the methanolysis system effectively facilitated the methylation process of PET, resulting in higher yields of 81% and 85% DMT, respectively. This finding confirmed that DMC promotes the depolymerization of PET. As a result, the yield of DMT is significantly higher compared to the PET methanolysis system operating alone.

Subsequently, we compared the reaction rate of the overall esterolysis of PET (Reaction 1) with the transesterification of EG with DMC (Reaction 3). The yields of DMT and EC reflected the reaction rates of methylation and esterification. The results indicated that the transesterification of EG with DMC occurs rapidly under the catalysis of [EMIm][OAc]. DMC rapidly reacts with EG (Reaction 2), leading to a 90% yield of EC within 20 minutes. The maximum production of EC in 92% yield was reached within 40 minutes. This result demonstrated that the transesterification of DMC with EG (Reaction 3) progresses significantly faster than the depolymerization of PET (Reaction 1), indicating that the PET depolymerization is the rate-determining step of this system.

5. Study on the causes of initial esterolysis of PET

5.1 The effect of water on reactions

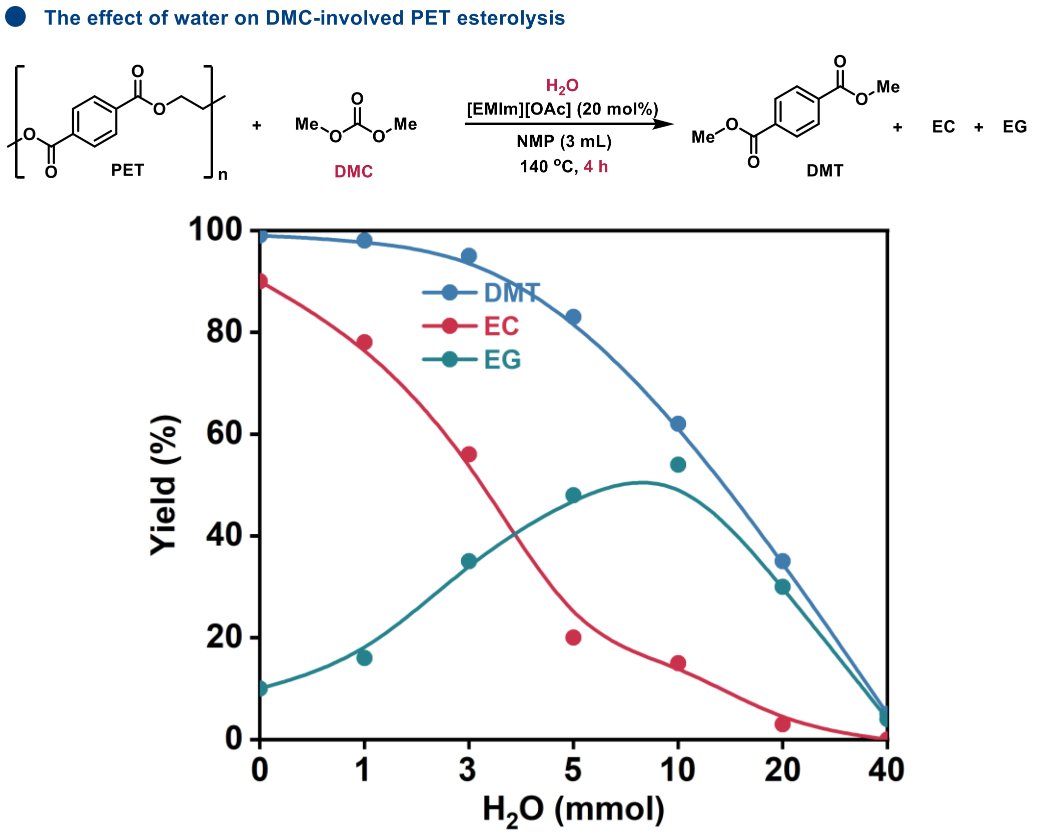

Fig. S8. The effect of amounts of water on PET esterolysis reaction. Standard reaction conditions: PET (1 mmol), DMC (4 mmol), [EMIm][OAc] (20 mol%), and NMP (3 mL) at 140 °C for 4 h. Yields were determined by ^{1}H NMR analysis with mesitylene as internal standard.

During the PET esterolysis process, the influence of water quantity on the reaction was investigated under standard conditions. An increase in the amount of water gradually reduced the PET conversion rate after four hours, influenced by varying water contents. Specifically, the initial addition of water had a gradual negative impact on the yields of DMT and EC; however, as the water amount increased from 1 to 10 mmol, the yield exhibited a gradual rise. Conversely, the further increase in the amount of water, the yields of all three products declined. Notably, when the amount of water reached 40 mmol, the depolymerization of PET was markedly insufficient. These results may be attributed to the fact that a small quantity of water has a minimal effect on the depolymerization of PET to yield DMT, while the presence of water may significantly enhance the hydrolysis of both EC, leading to the production of methanol and EG.

5.2. Exploring the effects of both trace amount of H_2O and MeOH on the esterolysis of PET

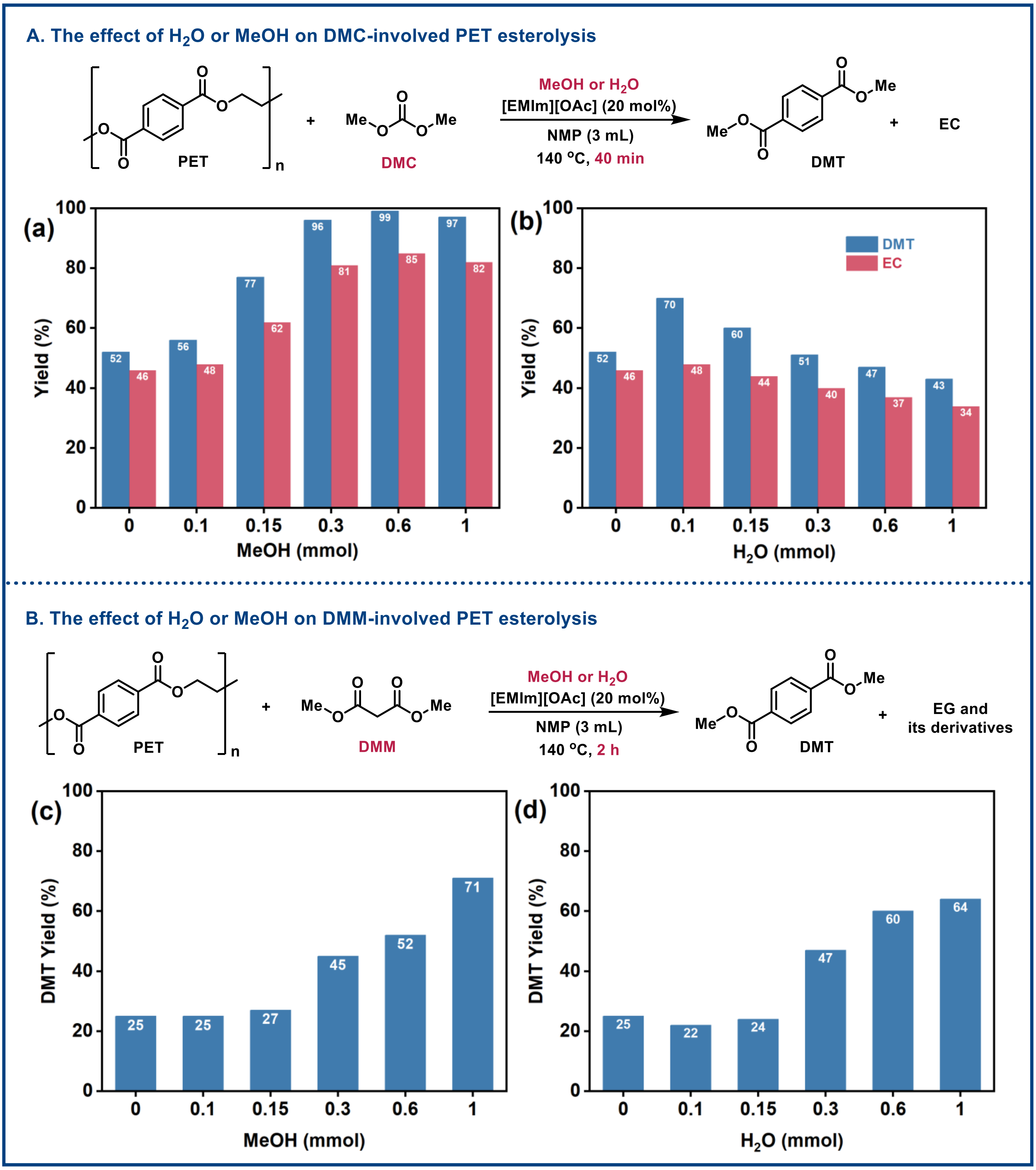

Fig. S9. The effect of MeOH or water content on DMC or DMM-involved PET esterolysis. A. Standard reaction conditions: PET (1 mmol), DMC (4 mmol), [EMIm][OAc] (20 mol%), and NMP (3 mL) at 140°C for 40 min. Yields were determined by ^{1}H NMR analysis with mesitylene as internal standard. B. Standard reaction conditions: PET (1 mmol), DMM (6 mmol), [EMIm][OAc] (20 mol%), and NMP (3 mL) at 140°C for 2 h. Yields were determined by ^{1}H NMR analysis with mesitylene as internal standard.

Typically, DMC hydrolysis may produce MeOH and CO_2 at elevated temperatures in the presence of H_2O and a catalyst, which may initiate the depolymerization of PET by nucleophilic attack of MeOH on ester

bond of PET.[24,25] To investigate this hypothesis, we examined the effects of both MeOH and H_2O concentrations on the reaction after 40 minutes (Fig. S9A). These results indicated that the trace amounts of MeOH can significantly enhance the PET depolymerization process. In contrast, the water content had a minimal effect, with MeOH demonstrating a more pronounced promotional effect compared to H_2O. Conversely, excessive amounts of H_2O gradually inhibited the progress of the reaction (Fig. S8 and S9A).

The effects of both water and methanol contents on the DMM-involved PET esterolysis were also investigated. The yield variations of the product DMT were detected after reacting at 140 °C for 2 hours and the results illustrated in Fig. S9B. When DMM was employed as the methyl source, both the addition of MeOH and H_2O enhanced the conversion of PET into DMT (2 hours); however, this enhancement is less pronounced compared to the DMC-involved PET esterolysis (40 minutes).

5.3. Exploring the causes of DMC decomposition

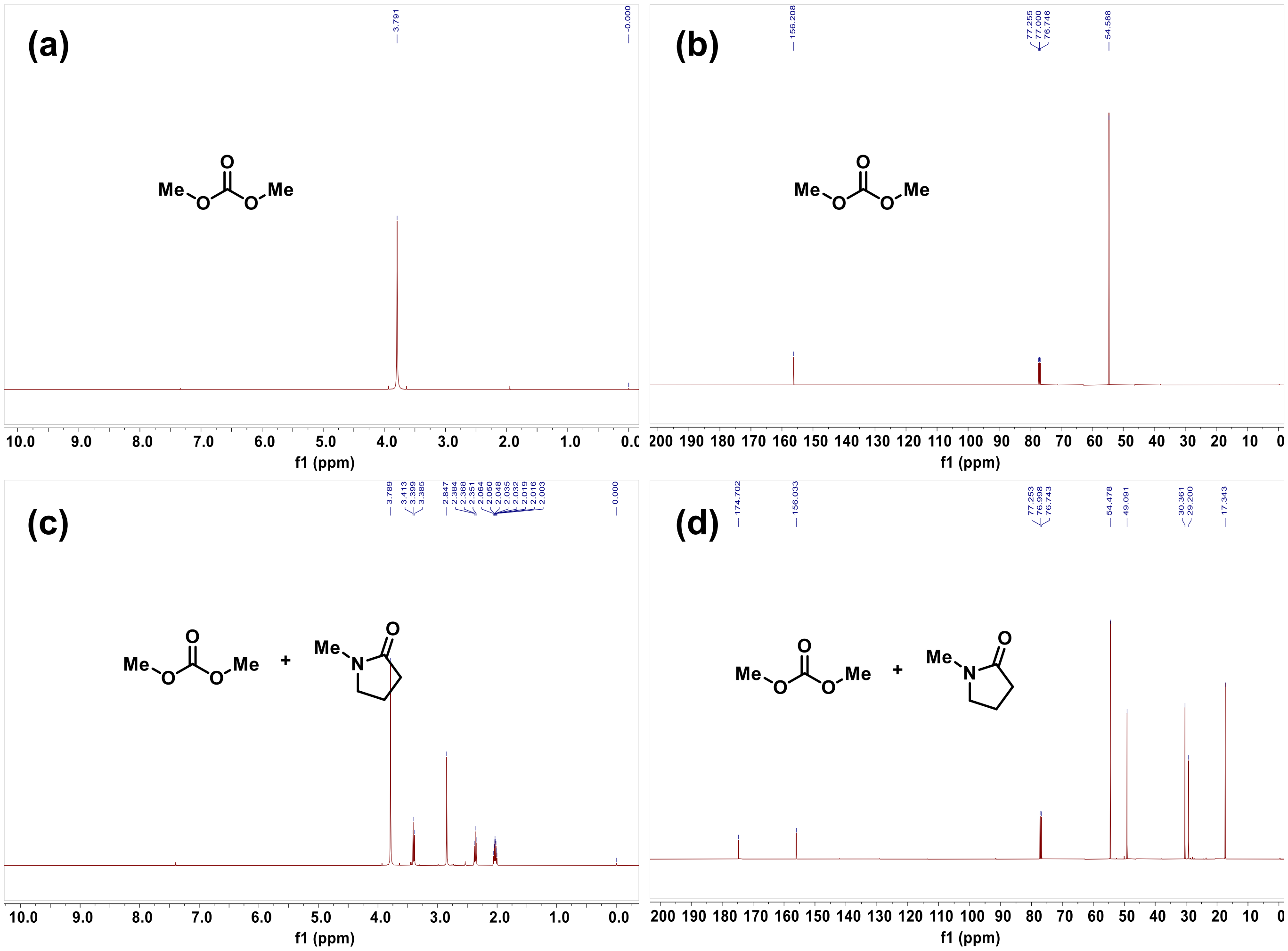

Fig. S10. NMR spectrum (600 MHz, $CDCl_3$, 298 K) of DMC processed in different ways. (a) ^{1}H NMR spectrum of pure DMC at 140 °C for 4 h. (b) ^{13}C NMR spectrum of pure DMC at 140 °C for 4 h. (c) ^{1}H NMR spectrum of DMC in NMP at 140 °C for 4 h. (d) ^{13}C NMR spectrum of DMC in NMP at 140 °C for 4 h.

To verify the source of methanol in the PET esterolysis reaction system, we conducted relevant control experiments. Pure DMC was reacted at 140 °C for 4 hours and subsequently analyzed using ^{1}H and ^{13}C NMR. The ^{1}H and ^{13}C NMR results indicated that DMC did not decompose to produce methanol. Furthermore, the reaction between DMC and NMP was performed at 140 °C for 4 hours, and trace amount of methanol was detected.

5.4. Exploring the causes of PET decomposition

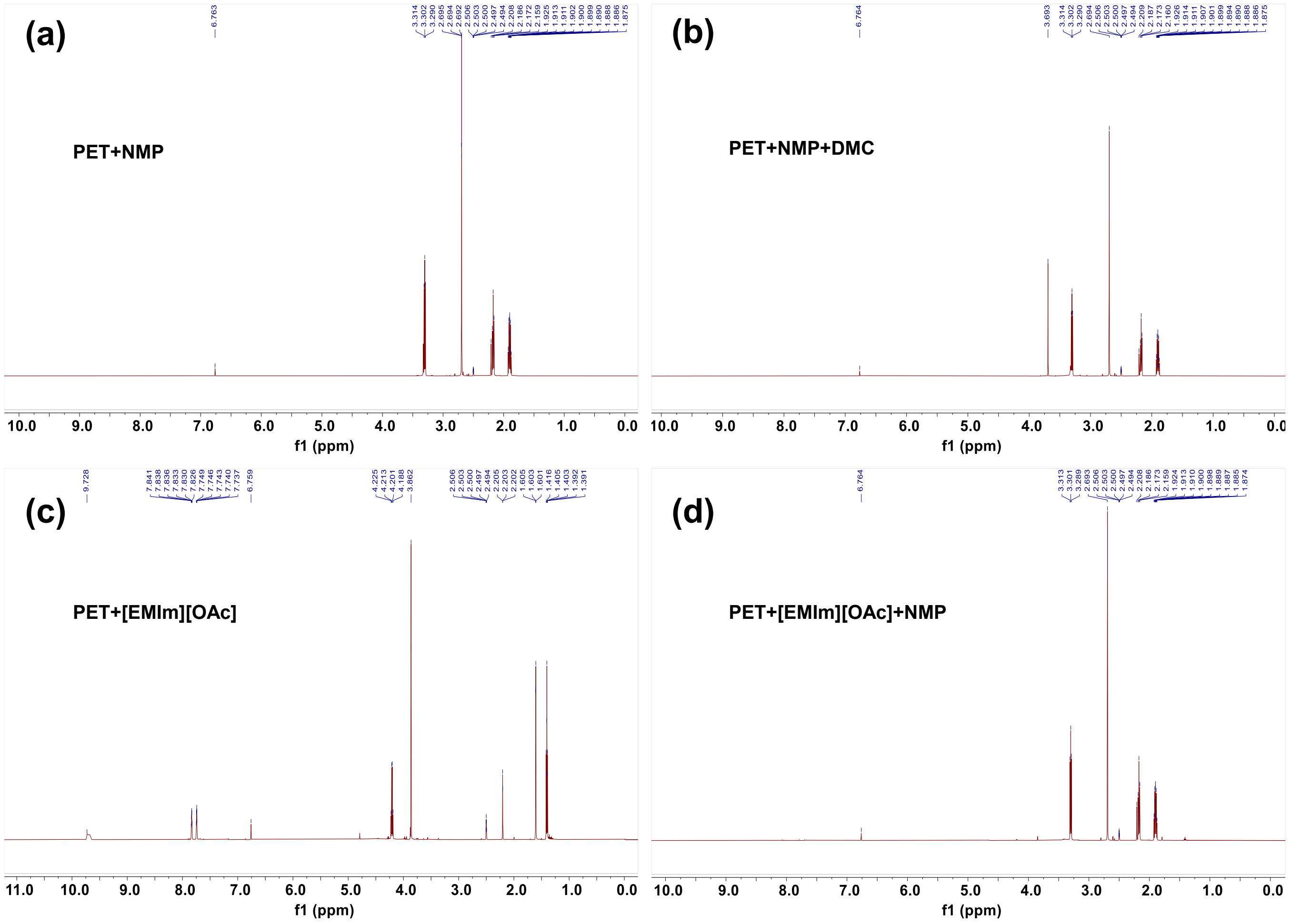

Fig. S11. NMR spectrum (600 MHz, DMSO-*d6*, 298 K) of the reaction mixture after processing PET in different ways. (a) ^{1}H NMR spectrum of PET (1 mmol) in NMP (3 mL) at 140 °C for 4 h. (b) ^{1}H NMR spectrum of PET (1 mmol) and DMC (4 mmol) in NMP (3 mL) at 140 °C for 4 h. (c) ^{1}H NMR spectrum of PET in [EMIm][OAc] (3 mL) at 140 °C for 4 h. (d) ^{1}H NMR spectrum of and PET (1 mmol) and [EMIm][OAc] (0.2 mmol) in NMP (3 mL) at 140 °C for 4 h.

To further validate other potential pathways that could induce PET degradation, we investigated the interactions between PET and other reactants (NMP and [EMIm][OAc]), with the results shown in Fig. S12. The ^{1}H NMR results indicated that NMP does not induce PET depolymerization, either in the presence or absence of DMC, as evidenced by the lack of aromatic compounds in the spectrum, aside from the internal standard mesitylene in DMSO-*d6* (Fig. S12a and S12b). When PET was reacted with the [EMIm][OAc] alone or in the presence of NMP, no obvious depolymerization of PET was observed (Fig. S11c and S11d).

5.5. Controlled experiments on PET depolymerization

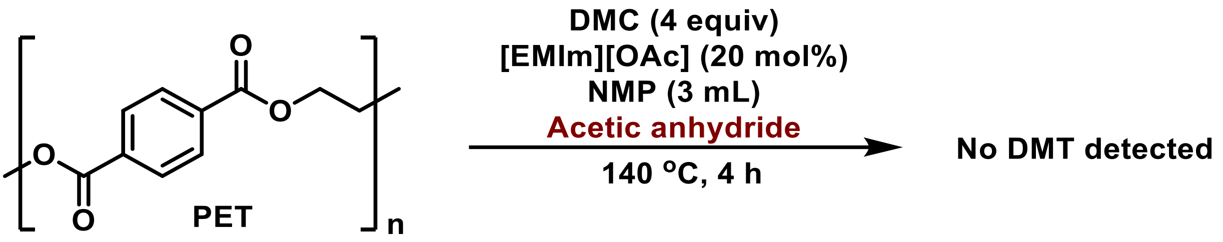

Fig. S12. Controlled experiments on the effect on PET depolymerization. The product yields are determined based on ^{1}H NMR analysis of the reaction mixture in DMSO-*d*6.

When acetic anhydride was added to the standard system, the esterolysis of PET to DMT did not occur. This result further underscored the critical role of DMC hydrolysis in initiating the depolymerization of PET.

5.6. In-situ production of methanol from DMM decomposition catalyzed by [EMIm][OAc]

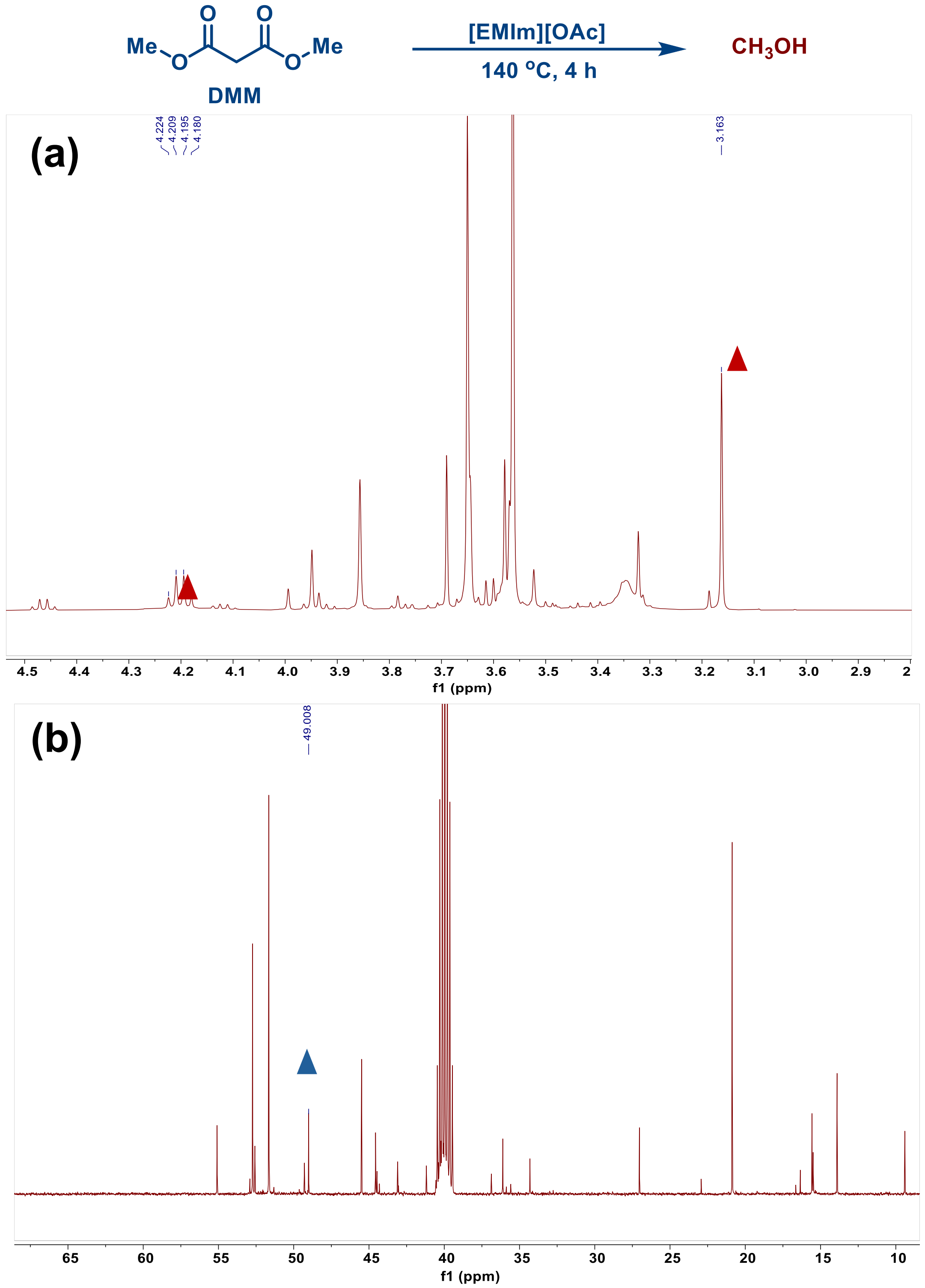

Fig. S13. Verification of MeOH produced during DMM-involved PET esterolysis. Standard reaction conditions: DMM (1 mmol) and [EMIm][OAc] (0.2 mmol) at 140 °C for 4 h. (a) ^{1}H NMR analysis of MeOH production (600 MHz, DMSO-*d*6, 298 K) (b) ^{13}C NMR analysis of MeOH production (600 MHz, DMSO-*d*6, 298 K).

We further independently verified the interaction between DMM and [EMIm][OAc] without the addition of other substances 140 °C for 4 hours. The in-situ production of MeOH, via DMM hydrolysis when catalyzed by [EMIm][OAc], was verified by NMR analysis. Both the^{1}H and ^{13}C NMR results indicated that DMM

decomposed to MeOH catalyzed by [EMIm][OAc]. This result confirmed that the MeOH produced during DMM-involved PET esterolysis was due to the hydrolysis of DMC catalyzed by [EMIm][OAc].

6. Characterization of possible hydrogen bond formation during PET esterolysis

6.1. ^{1}H NMR characterization of possible hydrogen bonds

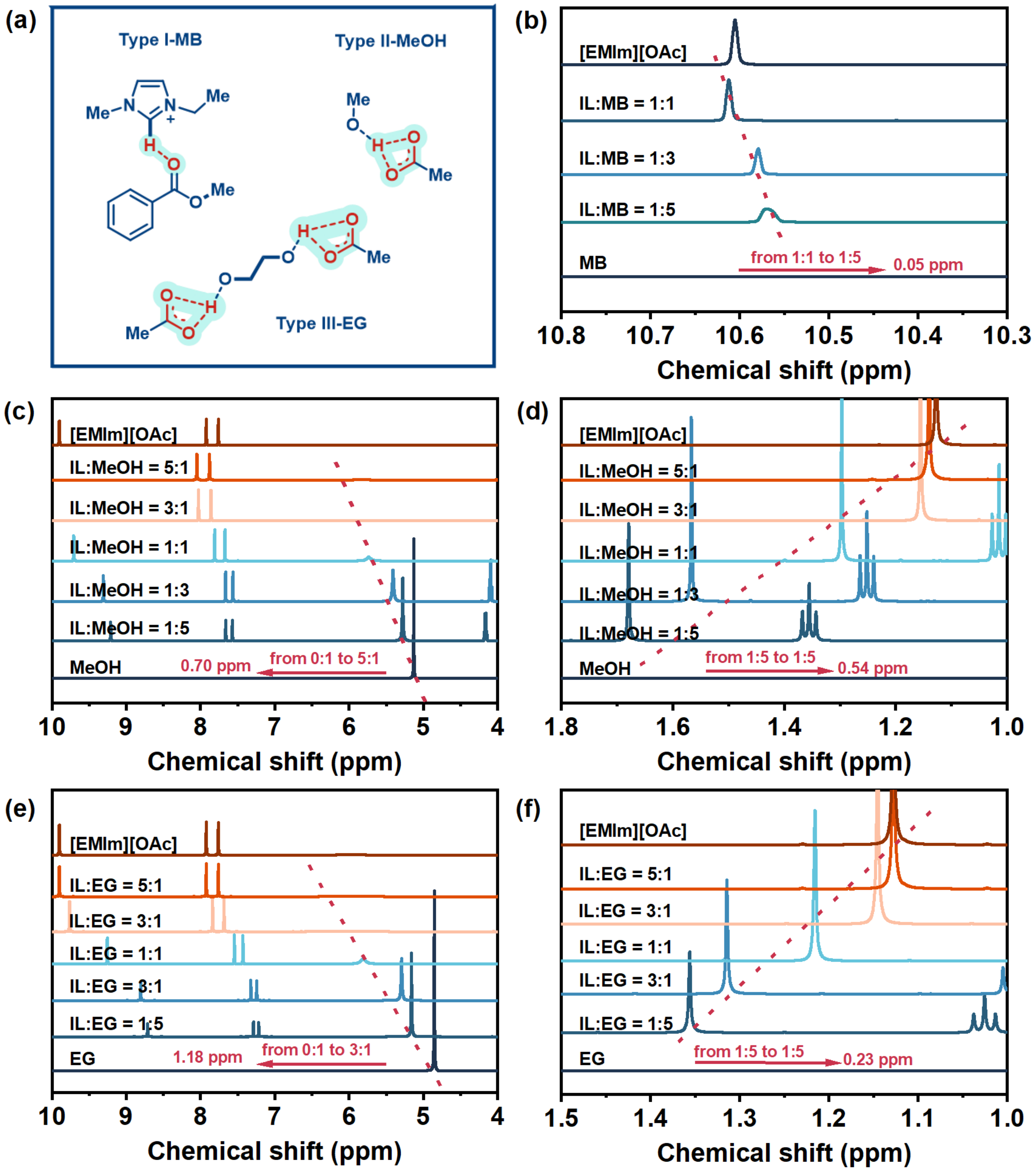

Fig. S14. ^{1}H NMR spectra (600 MHz, DMSO-*d6*, 298 K) of the [EMIm][OAc] and different reactants mixture with different molar ratios.

Based on previous studies[18,20,26-28], the proposed PET esterolysis process potentially involves the formation of multiple hydrogen bonds between [EMIm][OAc] and the reactants. To verify this, pure reactants (methyl benzoate, MeOH, and EG) were utilized, and their interactions with [EMIm][OAc] were examined via ^{1}H NMR. The hydrogen chemical shifts are illustrated in Fig. S14.

In the ^{1}H NMR spectra of mixtures containing methyl benzoate and [EMIm][OAc], the C-2 hydrogen of $[EMIm]^+$ consistently shifted to a higher field as the concentration of methyl benzoate increases (Fig. S14a). Specifically, the C-2 hydrogen of $[EMIm]^+$ shifted from 10.61 to 10.56 ppm as the ratio of [EMIm][OAc] to methyl benzoate changed from 1:1 to 1:5. This indicated the formation of hydrogen bonds between the C-2 hydrogen of $[EMIm]^+$ and the carbonyl oxygen of the reactants (Fig. S15, hydrogen bond Type I with methyl benzoate).[18]

The interaction between alcohols (MeOH and EG) and [EMIm][OAc] was also investigated (Fig. S14c-S14f). The single peak corresponding to the hydroxyl hydrogen of MeOH appeared at 5.13 ppm. Notably, the chemical shift of the hydroxyl hydrogen of MeOH shifted downfield from 5.13 to 5.83 ppm when the molar ratio of [EMIm][OAc] to MeOH changed from 1:5 to 1:1 (Fig. S14a, hydrogen bond Type II). Similarly, a significant downfield shift from 4.85 to 6.03 ppm of the hydroxyl hydrogens of EG was observed as the concentration of [EMIm][OAc] increased from 1:0 to 1:5 (Fig. S14a, hydrogen bond Type III). This shift indicated the formation of a robust hydrogen bond between the hydroxyl group of the alcohols and the acetate anion in [EMIm][OAc]. The chemical shift of the methyl hydrogen of $[OAc]^-$ shifted toward the high field as the concentration of [EMIm][OAc] increases, regardless of whether it is mixed with MeOH or EG. This observation further confirmed the presence of strong hydrogen bonding between [EMIm][OAc] and alcohols (MeOH and EG).

6.2. FT-IR characterization of possible hydrogen bonds

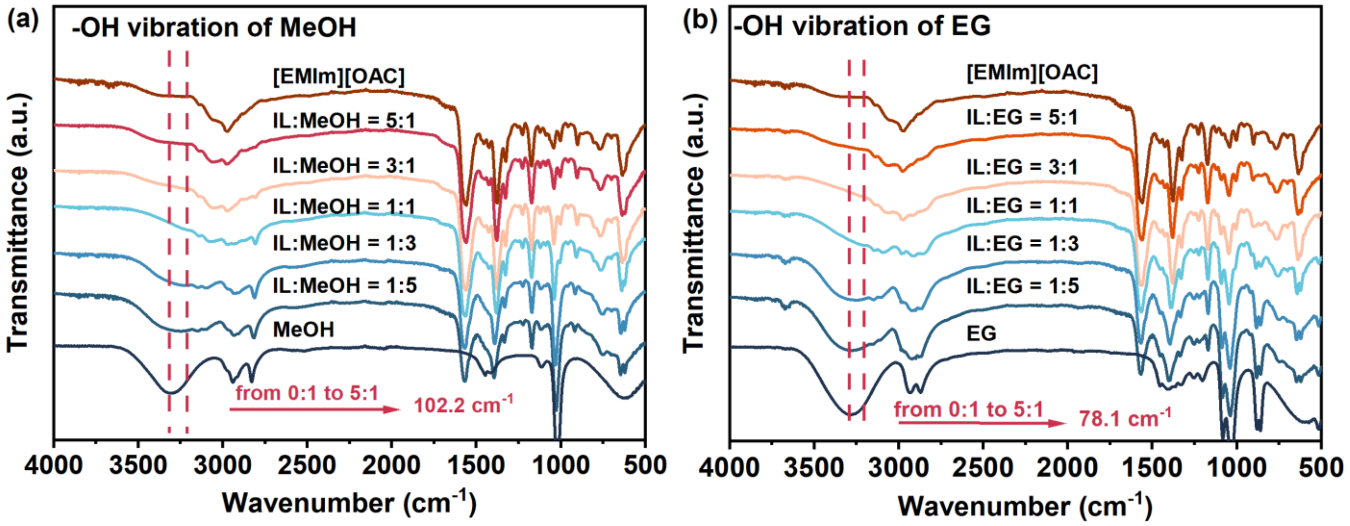

Fig. S15. FT-IR spectra of the [EMIm][OAc] and different reactants mixture with different molar ratios.

Furthermore, the interaction of alcohols (MeOH and EG) with [EMIm][OAc] was examined via FT-IR analysis. As the molar ratio of [EMIm][OAc] versus alcohols increased from 0:1 to 5:1, the -OH vibration of alcohols gradually broadened and nearly disappeared in the FT-IR spectrum. The redshift of -OH in MeOH was 102.2 cm^{-1}, from 3316.5 to 3214.3 cm^{-1} (Fig. S15a). The redshift of -OH in EG was 78.1 cm^{-1}, from 3282.7 to 3204.6 cm^{-1} (Fig. S15b). The observed redshifts were ascribed to the formation of robust hydrogen bonds between the oxygen of the acetate anion and the hydrogen of the hydroxyl group in alcohols.[18,20] In summary, the above results suggested that [EMIm][OAc] boosts the reactivity of reactants by increasing the electropositivity of the carbonyl carbon and the electronegativity of the hydroxyl oxygen, respectively. This makes PET esterolysis and EG transesterification with DMC more favorable.

7. Study on the influence of functional groups on the benzene ring of carboxylic acids

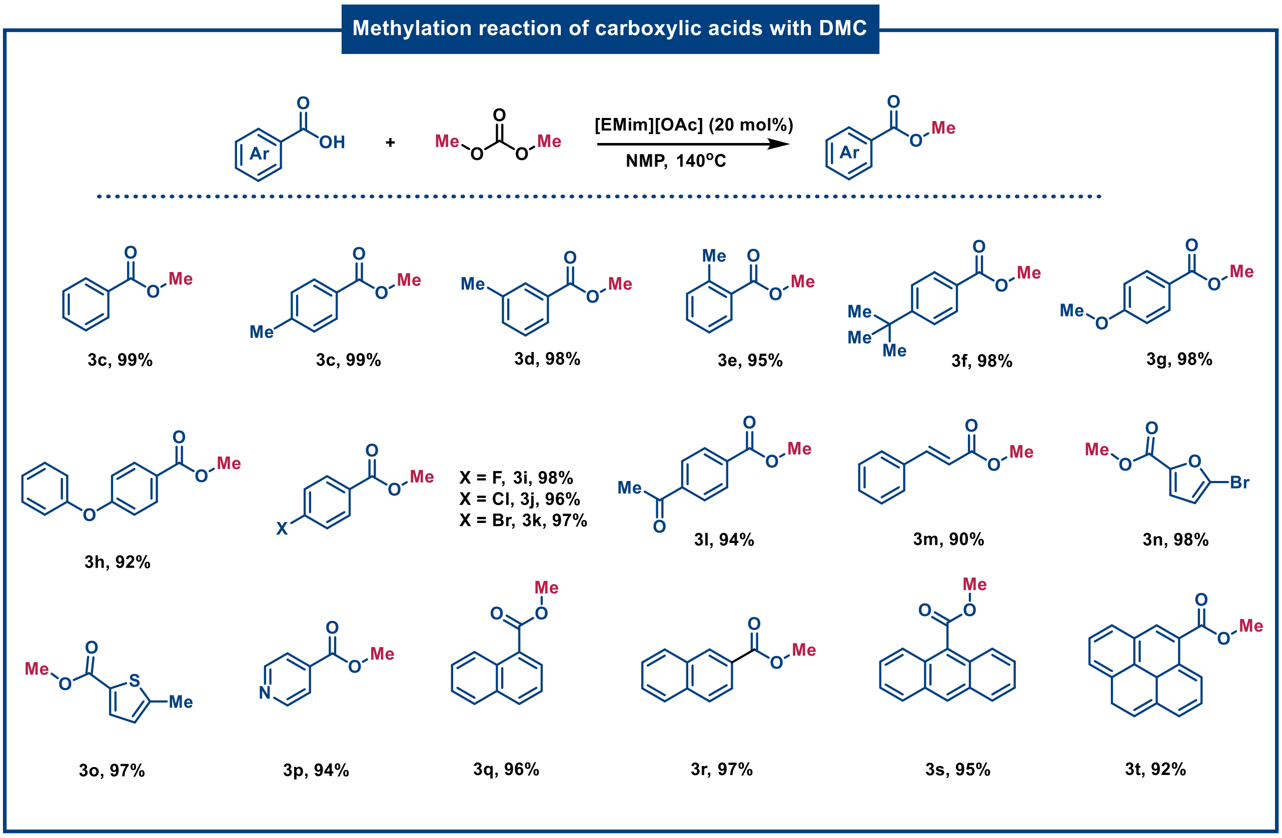

Fig. S16. Methylation reaction of carboxylic acids with DMC. [a] Standard reaction conditions: Carboxylic acid or other monomer (1 mmol), DMC (4 mmol), [EMIm][OAc] (20 mmol%), and NMP (3 mL) at 140 °C for 4 h. [b] Yields were determined by ^{1}H NMR analysis (600 MHz, $CDCl_3$, 298 K) with mesitylene as internal standard.

Methylation of carboxylic acids is a crucial tool for accessing chemicals and plays a pivotal role in medicinal chemistry, natural product synthesis, and reaction development.[29] Given the significance of developing carboxylic acid methylation strategies to investigate the influence of functional groups on the benzene ring of benzoic acid, the effectiveness of methylation in carboxylic acids with DMC was further explored. Fig. S17 confirmed that this strategy features a broad carboxylic acid substrate scope with excellent electronic and steric tolerance. Specifically, *para*-, *meso*-, and *meta*-substituted benzoic acids experienced negligible site-blocking effects and were transformed into the corresponding methyl carboxylate 3c-3e, with yields ranging from 95% to 99%. Carboxylic acids with electron-donating (*p*-*t*-Bu, *p*-OMe, and *p*-OPh) or electron-withdrawing groups (*p*-F, *p*-Cl, *p*-Br and *p*-$COCH_3$) were well-tolerated, leading to the formation of carboxylates 3f-3l with yields ranging from 94% to 98%. Additionally, styryl, fused aromatic or heteroaromatic rings-substituted carboxylic acids undergo the methylation reaction to form methyl carboxylates 3m-3t in yields ranging from 80% to 98%.

8. The possible reaction mechanism

8.1 The possible reaction mechanism of DMC-involved PET esterolysis

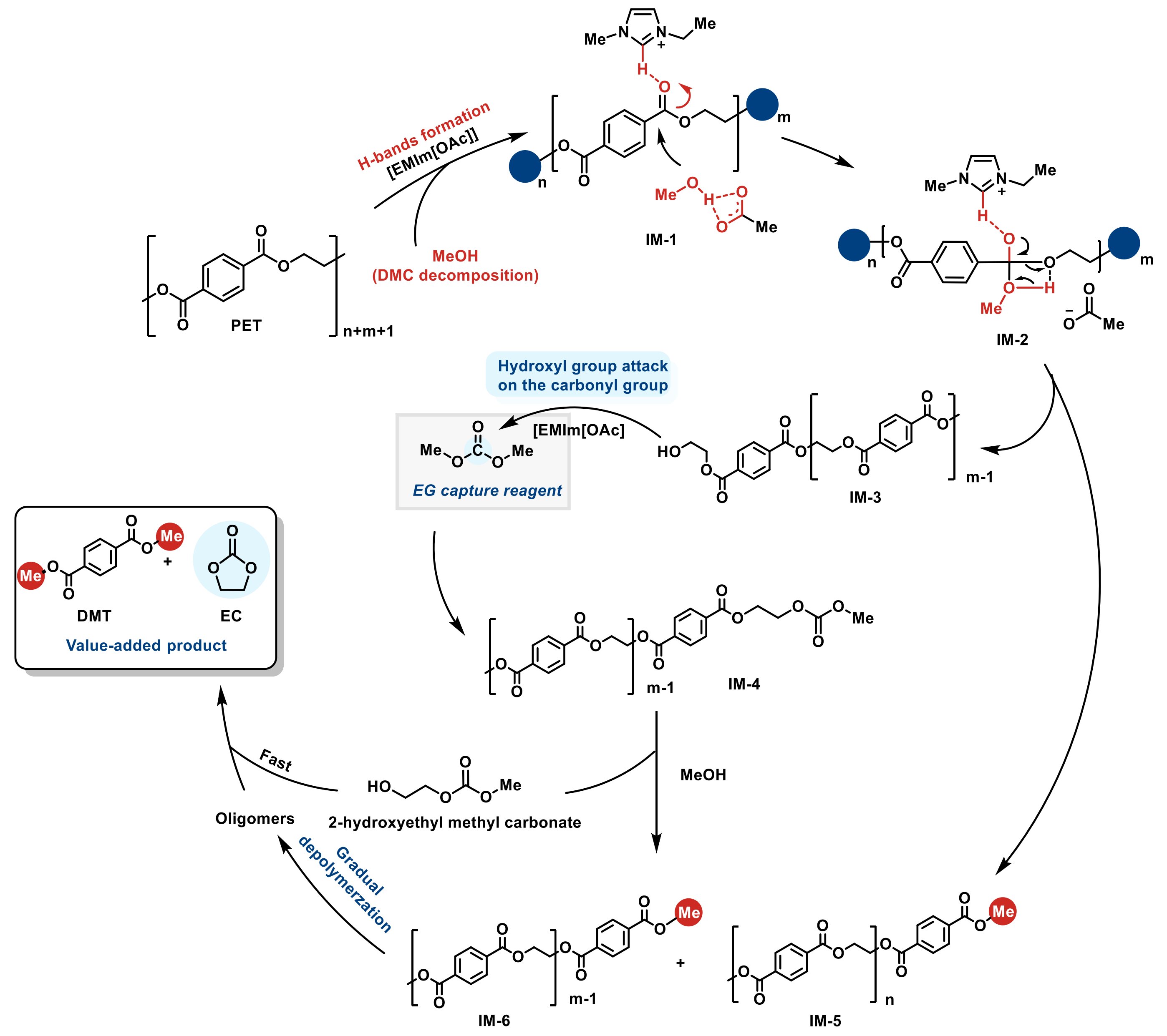

Fig. S17. The possible reaction mechanism of DMC-involved PET esterolysis catalyzed by [EMIm][OAc].

Based on our latest experimental data, we present a DMC-involved PET esterolysis mechanism facilitated by [EMIm][OAc], as outlined in Fig. S17. The dual functionality of the anion and cation of [EMIm][OAc] activates both MeOH derived from DMC decomposition and the oxygen in the carboxyl group of PET by forming H-bonds. This interaction triggers electron transfer, resulting in the formation of carbonyl carbocation. Afterward, MeOH, acting as a nucleophilic reagent, reacts with carbonyl carbocation, initiating the formation of a tetrahedral intermediate IM-2. This intermediate gradually decomposed via intramolecular C-O bond cleavage, resulting in the formation of intermediates IM-3 and IM-5, respectively. Intermediate IM-3 containing the EG unit undergoes preferential transesterification with DMC to produce IM-4. Subsequently, IM4 reacts with MeOH to form 2-hydroxyethyl methyl carbonate (fast intramolecular cyclization to produce

EC) and the methylated intermediate IM-6. As the oligomers fragment or cleave, the system efficiently produces small monomer molecules through transesterification reactions. These small molecules then undergo transesterification with MeOH or DMC, ultimately resulting in the production of the desired value-added products DMT and EC.

8.2 The possible reaction mechanism of DMM-involved PET esterolysis

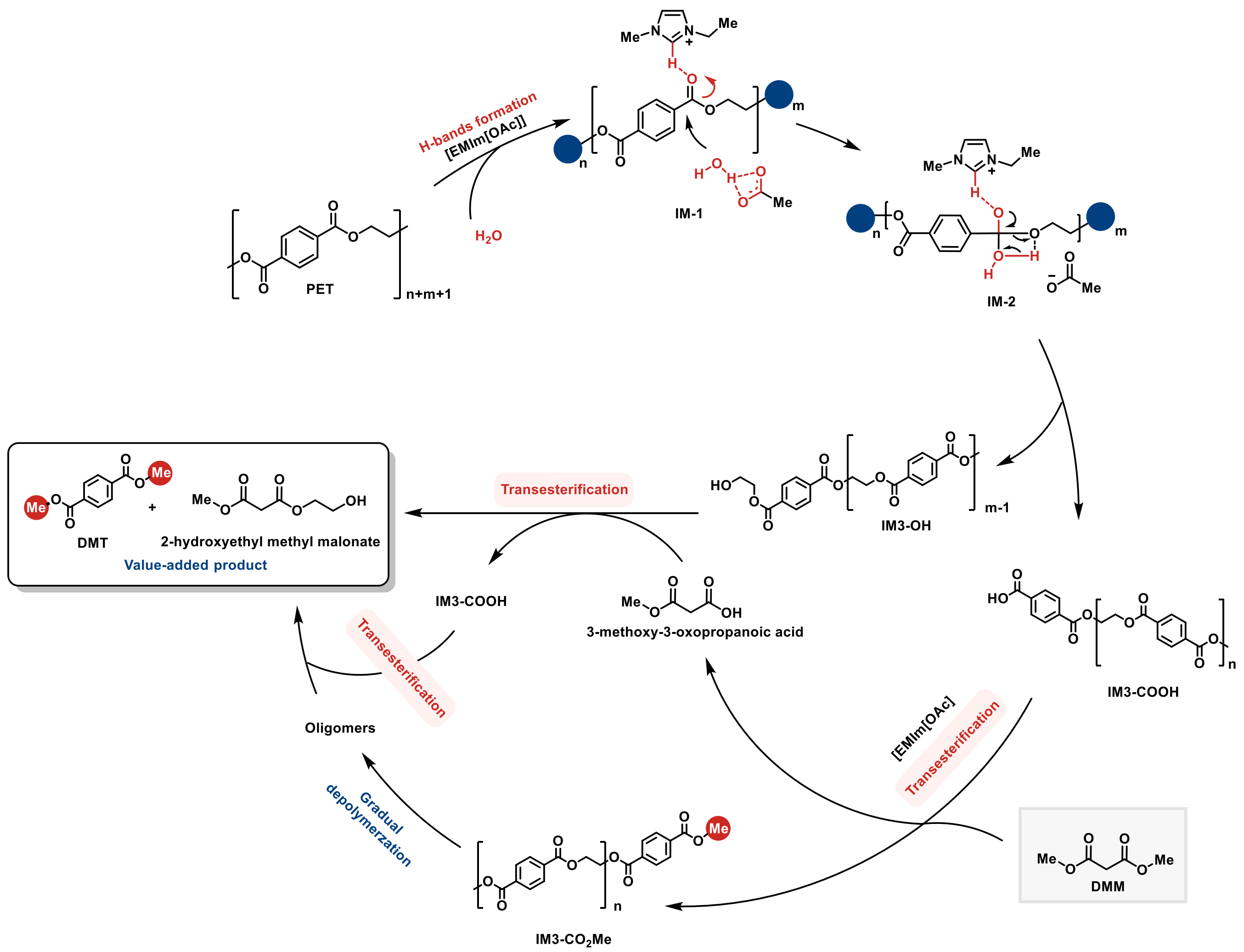

Fig. S18. The possible reaction mechanism of DMM-involved PET esterolysis catalyzed by [EMIm][OAc].

Based on experimental evidence for the promoting effect of water on PET esterolysis involving carboxylic acid ester substrates and the methylation of carboxylic acid intermediates (as illustrated in Fig.s 5B, 5C, S9, and S16), we propose that although methanolysis remains the dominant pathway in the system, DMM-involved PET esterolysis may also proceed via hydrolysis and involve direct methylation of the carboxylic acid intermediates by DMM.

9. ^{1}H NMR and ^{13}C NMR characterization date of all compounds

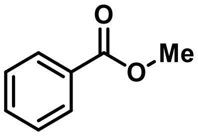

Methyl benzoate **3b** [30]: colorless oil, ^{1}H NMR yield 98%; ^{1}H NMR (600 MHz, $CDCl_3$) δ = 8.22 (d, *J* = 8.4 Hz, 2H), 7.55 (t, *J* = 9.0 Hz, 2H), 7.43 (t, *J* = 9.6 Hz, 3H), 3.91 (s, 3H); ^{13}C NMR (151 MHz, $CDCl_3$) δ = 167.1, 132.9, 130.1, 129.5, 128.3, 52.0.

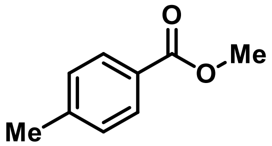

Methyl 4-methylbenzoate **3c** [31]: colorless oil, ^{1}H NMR yield 99%; ^{1}H NMR (600 MHz, $CDCl_3$) δ = 7.93 (d, *J* = 10.2 Hz, 2H), 7.23 (d, *J* = 9.6 Hz, 2H), 3.89 (s, 3H), 3.92 (s, 3H); ^{13}C NMR (151 MHz, $CDCl_3$) δ = 167.1, 143.5, 129.5, 129.0, 127.4, 51.9, 21.6.

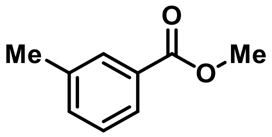

Methyl 3-methylbenzoate **3d** [32]: white solid, ^{1}H NMR yield 98%; ^{1}H NMR (600 MHz, $CDCl_3$) δ = 7.86 (s, 1H), 7.83 (d, *J* = 9.0 Hz, 1H), 7.37-7.30 (m, 2H),3.90 (s, 3H), 2.39 (s, 3H); ^{13}C NMR (151 MHz, $CDCl_3$) δ = 167.2, 138.1, 133.6, 130.1, 130.0, 128.2, 126.6, 52.0, 21.2.

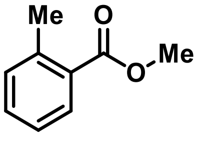

Methyl 2-methylbenzoate **3e** [30]: colorless oil, ^{1}H NMR yield 95%; ^{1}H NMR (600 MHz, $CDCl_3$) δ = 7.93 (d, *J* = 9.6 Hz, 1H), 7.23 (t, *J* = 9 Hz, 1H), 7.25-7.21 (m, 3H), 3.88 (s, 3H) , 2.60 (s, 3H); ^{13}C NMR (151 MHz, $CDCl_3$) δ = 168.0, 140.1, 131.9, 131.6, 130.5, 129.5, 125.6, 51.7, 21.6.

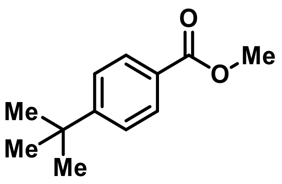

Methyl 4-(*tert*-butyl)benzoate **3f** [30]: colorless oil, ^{1}H NMR yield 98%; ^{1}H NMR (600 MHz, $CDCl_3$) δ = 7.97 (d, *J* = 8.4 Hz, 2H), 7.43 (d, *J* = 8.4 Hz, 2H), 3.88 (s, 3H), 1.322 (s, 9H); ^{13}C NMR (151 MHz, $CDCl_3$) δ = 166.9, 156.3, 129.3, 127.3, 125.2, 51.7, 34.9, 31.0.

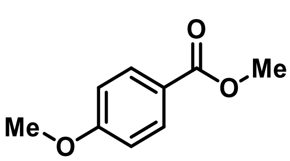

Methyl 4-methoxybenzoate **3g** [31]: colorless oil, ^{1}H NMR yield 98% (1 mmol of methyl 4-hydroxybenzoate as feedstocks, ^{1}H NMR yield 96%); ^{1}H NMR (600 MHz, $CDCl_3$) δ = 7.99 (d, *J* = 10.8 Hz, 2H), 6.91 (d, *J* = 10.8 Hz, 2H), 3.88 (s, 3H), 3.85 (s, 3H); ^{13}C NMR (151 MHz, $CDCl_3$) δ = 166.8, 163.3, 131.5, 122.6, 113.6, 55. 4, 51.8.

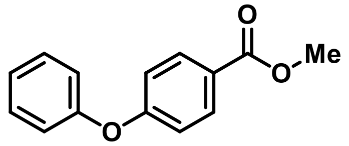

Methyl 4-phenoxybenzoate **3h** [33]: white solid, ^{1}H NMR yield 92%; ^{1}H NMR (600 MHz, $CDCl_3$) δ = 8.50 (d, *J* = 10.8 Hz, 2H), 7.39-7.36 (m, 2H), 7.19-7.16 (m, 1H), 7.07-7.05 (m, 2H), 6.98 (d, *J* = 10.8 Hz, 2H), 3.89 (s, 3H); ^{13}C NMR (151 MHz, $CDCl_3$) δ = 166.5, 164.7, 155.5, 131.6, 130.0, 130.0, 124.4, 120.0, 117.2, 51.9.

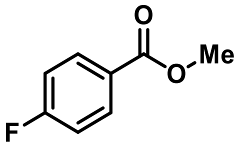

Methyl 4-fluorobenzoate **3i** [30]: colorless liquid, ^{1}H NMR yield 98%; ^{1}H NMR (600 MHz, $CDCl_3$) δ = 8.07-8.04 (m, 2H), 7.12-7.09 (m, 2H), 3.91 (s, 3H); ^{13}C NMR (151 MHz, $CDCl_3$) δ = 166.1, 165.7 (d, $J_{C\text{-}F}$ = 251.3 Hz), 132.1 (d, $J_{C\text{-}F}$ = 8.8 Hz), 126.4 (d, $J_{C\text{-}F}$ = 2.5 Hz) , 115.5 (d, $J_{C\text{-}F}$ = 22.5 Hz), 52.1.

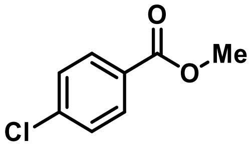

Methyl 4-chlorobenzoate **3j** [30]: white solid, ^{1}H NMR yield 96%; ^{1}H NMR (600 MHz, $CDCl_3$) δ = 7.96 (d, *J* = 10.2 Hz, 2H), 7.40 (d, *J* = 10.8 Hz, 2H), 3.91 (s, 3H); ^{13}C NMR (151 MHz, $CDCl_3$) δ = 166.1, 139.3, 130.9, 128.6, 128.5, 52.2.

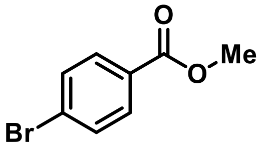

Methyl 4-bromobenzoate **3k** [30]: white solid, ^{1}H NMR yield 97%; ^{1}H NMR (600 MHz, $CDCl_3$) δ = 7.89 (d, *J* = 10.2 Hz, 2H), 7.57 (d, *J* = 10.2 Hz, 2H), 3.91 (s, 3H); ^{13}C NMR (151 MHz, $CDCl_3$) δ = 166.3, 131.6, 131.0, 129.0, 128.0, 52.2.

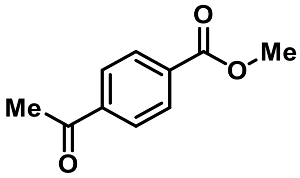

Methyl 4-acetylbenzoate **3l** [31]: white solid, ^{1}H NMR yield 94%; ^{1}H NMR (600 MHz, $CDCl_3$) δ = 8.12 (d, *J* = 10.2 Hz, 2H), 8.01 (d, *J* = 10.2 Hz, 2H), 3.95 (s, 3H), 2.65 (s, 3H); ^{13}C NMR (151 MHz, $CDCl_3$) δ = 197.5, 166.2, 140.2, 133.9, 129.8, 128.2, 52.5, 26.9.

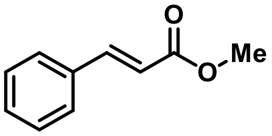

Methyl cinnamate **3m** [30]: white solid, ^{1}H NMR yield 90%; ^{1}H NMR (600 MHz, $CDCl_3$) δ = 7.60 (d, *J* = 19.2 Hz, 1H), 7.43-7.41 (m, 2H), 7.29-7.27 (m, 3H), 6.35 (d, *J* = 19.2 Hz, 1H), 3.71 (s, 3H); ^{13}C NMR (151 MHz, $CDCl_3$) δ = 167.3, 144.8, 134.3, 130.2, 128.8, 128.0, 117.7, 51.6.

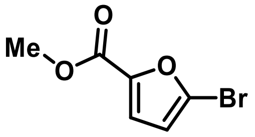

Methyl 5-bromofuran-2-carboxylate **3n** [31]: white solid, ^{1}H NMR yield 98%; ^{1}H NMR (600 MHz, $CDCl_3$) δ = 7.13 (d, *J* = 4.2 Hz, 2H), 6.46 (d, *J* = 4.2 Hz, 2H), 3.90 (s, 3H); ^{13}C NMR (151 MHz, $CDCl_3$) δ = 158.0, 146.1, 127.4, 120.1, 113.9, 52.0.

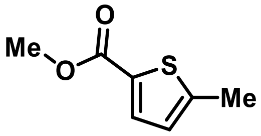

Methyl 5-methylthiophene-2-carboxylate **3o** [34]: colorless liquid, ^{1}H NMR yield 97%; ^{1}H NMR (600 MHz, $CDCl_3$) δ = 7.61 (d, *J* = 4.2 Hz, 2H), 6.76 (d, *J* = 4.8 Hz, 2H), 3.85 (s, 3H), 2.51 (s, 3H); ^{13}C NMR (151 MHz, $CDCl_3$) δ = 162.6, 147.9, 133.8, 130.8, 126.3, 51.9, 15.6.

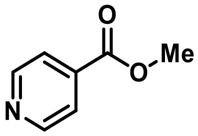

Methyl isonicotinate **3p** [35]: colorless liquid, ^{1}H NMR yield 94%; ^{1}H NMR (600 MHz, $CDCl_3$) δ = 8.78 (d, *J* = 7.2 Hz, 2H), 7.85 (d, *J* = 7.2 Hz, 2H), 3.96 (s, 3H); ^{13}C NMR (151 MHz, $CDCl_3$) δ = 165.4, 150.4, 137.1, 122.7, 52.5.

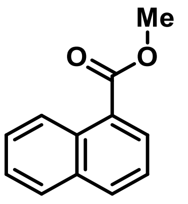

Methyl 1-naphthoate **3q** [31]: white solid, ^{1}H NMR yield 96%; ^{1}H NMR (600 MHz, $CDCl_3$) δ = 8.91 (d, *J* = 9.6 Hz, 1H), 8.16 (d, *J* = 8.4 Hz, 1H), 7.97 (d, *J* = 9.6 Hz, 1H), 7.84 (d, *J* = 11.4 Hz, 1H), 7.61-7.58 (m, 1H), 7.51-7.44 (m, 2H), 3.97 (s, 3H); ^{13}C NMR (151 MHz, $CDCl_3$) δ = 167.9, 133.7, 133.3, 131.2, 130.1, 128.5, 127.7, 127.0, 126.1, 125.7, 124.4, 52.0.

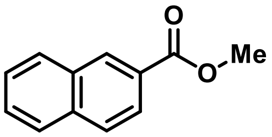

Methyl 2-naphthoate **3r** [31]: white solid, ^{1}H NMR yield 95%; ^{1}H NMR (600 MHz, $CDCl_3$) δ = 8.58 (s, 1H), 8.03 (dd, *J* = 10.8, 2.4 Hz, , 1H), 7.97 (d, *J* = 9.6 Hz, 1H), 7.83 (d, *J* = 10.8 Hz, 2H), 7.56-7.48 (m, 2H), 3.94 (s, 3H); ^{13}C NMR (151 MHz, $CDCl_3$) δ = 167.1, 135.4, 132.4, 130.9, 129.2, 128.1, 128.0, 127.6, 127.3, 126.5, 125.1, 52.1.

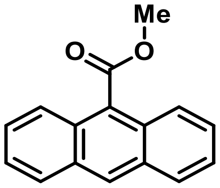

Methyl anthracene-9-carboxylate **3s** [30]: Yellow solid, ^{1}H NMR yield 95%; ^{1}H NMR (600 MHz, $CDCl_3$) δ = 8.50 (s, 1H), 8.01 (dd, *J* = 18.6, 10.8 Hz, 4H), 7.54-7.45 (m, 4H), 3.90 (s, 3H); ^{13}C NMR (151 MHz, $CDCl_3$) δ = 170.1, 130.9, 129.4, 128.6, 128.5, 127.7, 127.0, 125.4, 125.0, 52.6.

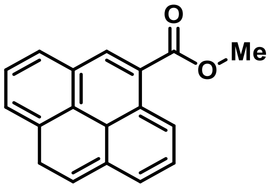

Methyl 3a^{1},9-dihydropyrene-4-carboxylate **3t** [36]: white solid, ^{1}H NMR yield 92%; ^{1}H NMR (600 MHz, $CDCl_3$) δ = 9.19 (d, *J* = 11.4 Hz, 1H), 8.53 (d, *J* = 9.6 Hz, 1H), 8.16-8.12 (m, 3H), 8.04-8.03 (m, 2H), 7.97-7.95 (m, 2H), 4.06 (s, 3H); ^{13}C NMR (151 MHz, $CDCl_3$) δ = 168.4, 134.2, 131.0, 130.8, 130.2, 129.5, 129.3, 128.3, 127.0, 126.1, 126.0, 124.8, 124.6, 124.0, 124.0, 123.3, 52.20.

10. ^{1}H NMR and ^{13}C NMR spectra of all compounds

^{1}H NMR of **3b** (600 MHz, $CDCl_3$) and ^{13}C NMR of **3b** (151 MHz, $CDCl_3$)

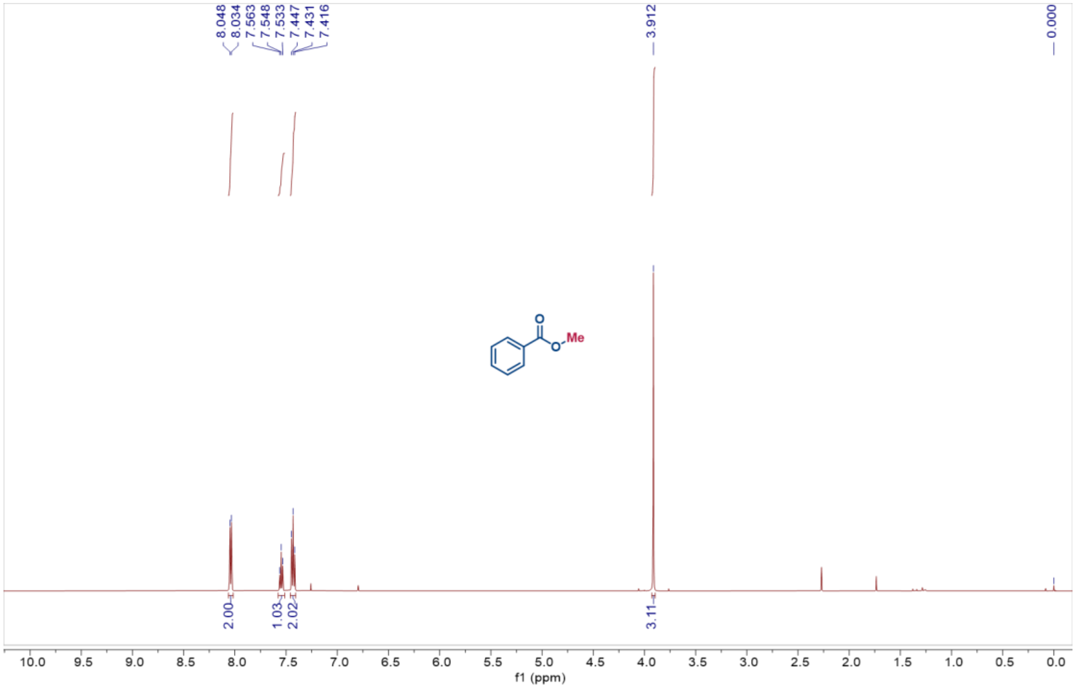

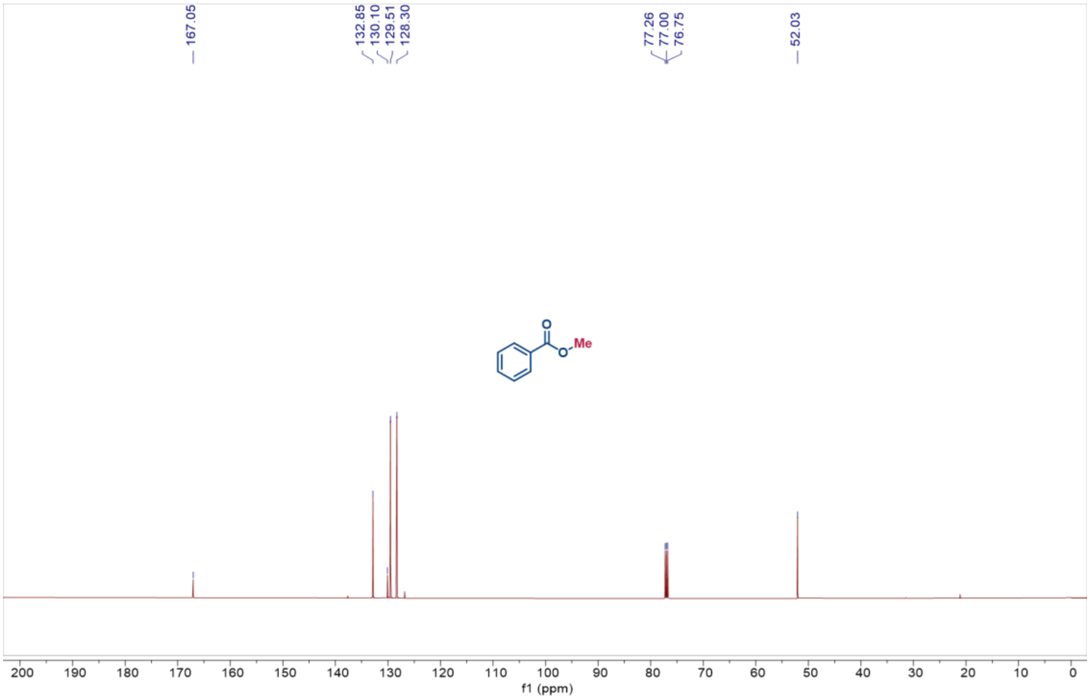

^{1}H NMR of **3c** (600 MHz, $CDCl_3$) and ^{13}C NMR of **3c** (151 MHz, $CDCl_3$)

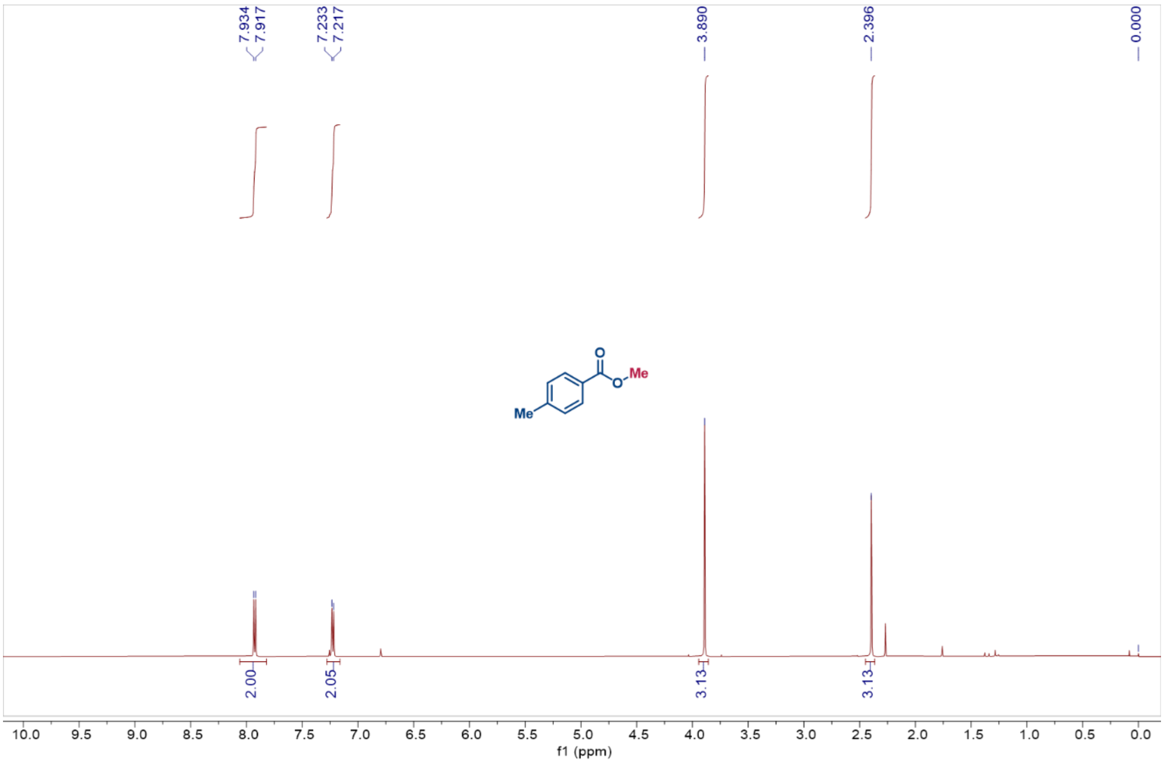

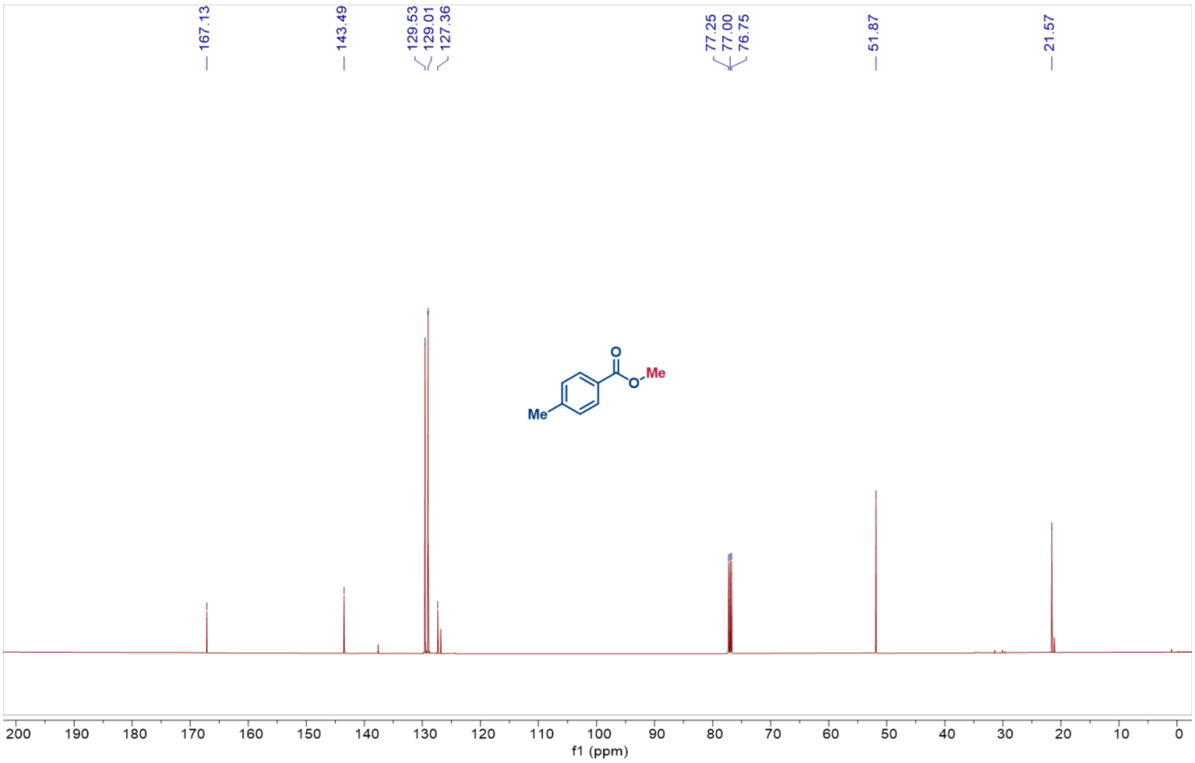

^{1}H NMR of **3d** (600 MHz, $CDCl_3$) and ^{13}C NMR of **3d** (151 MHz, $CDCl_3$)

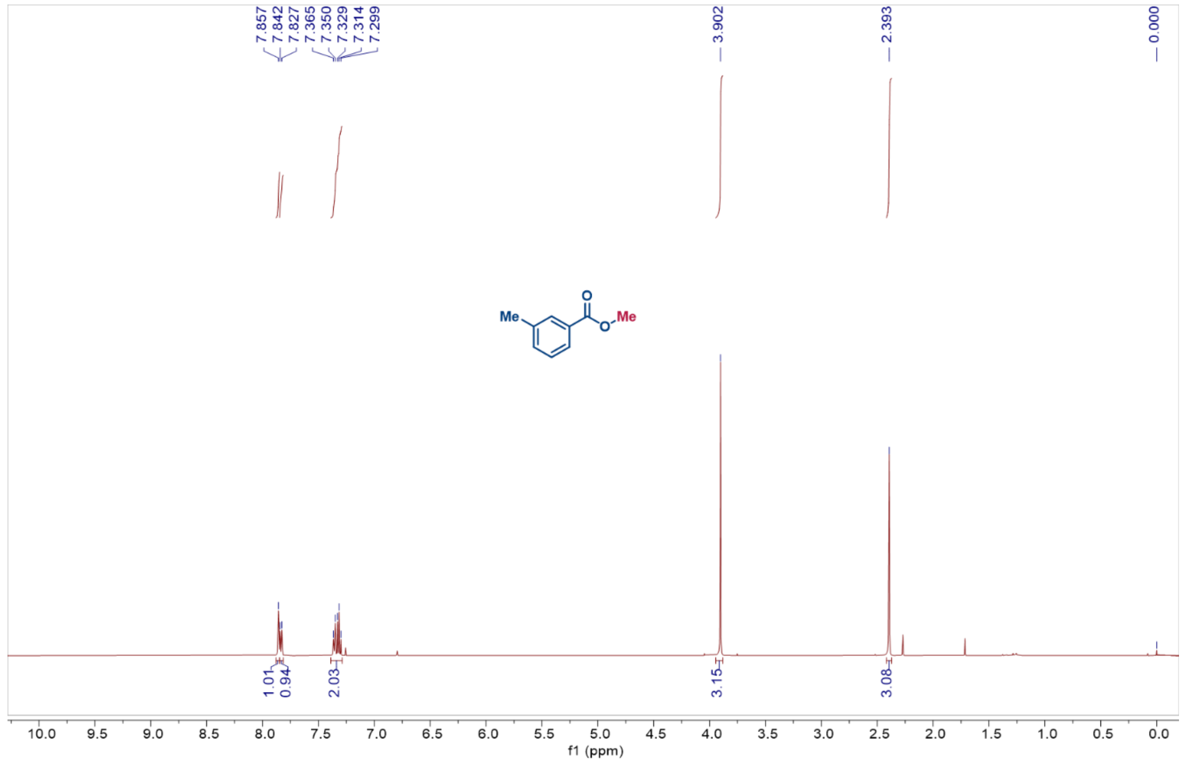

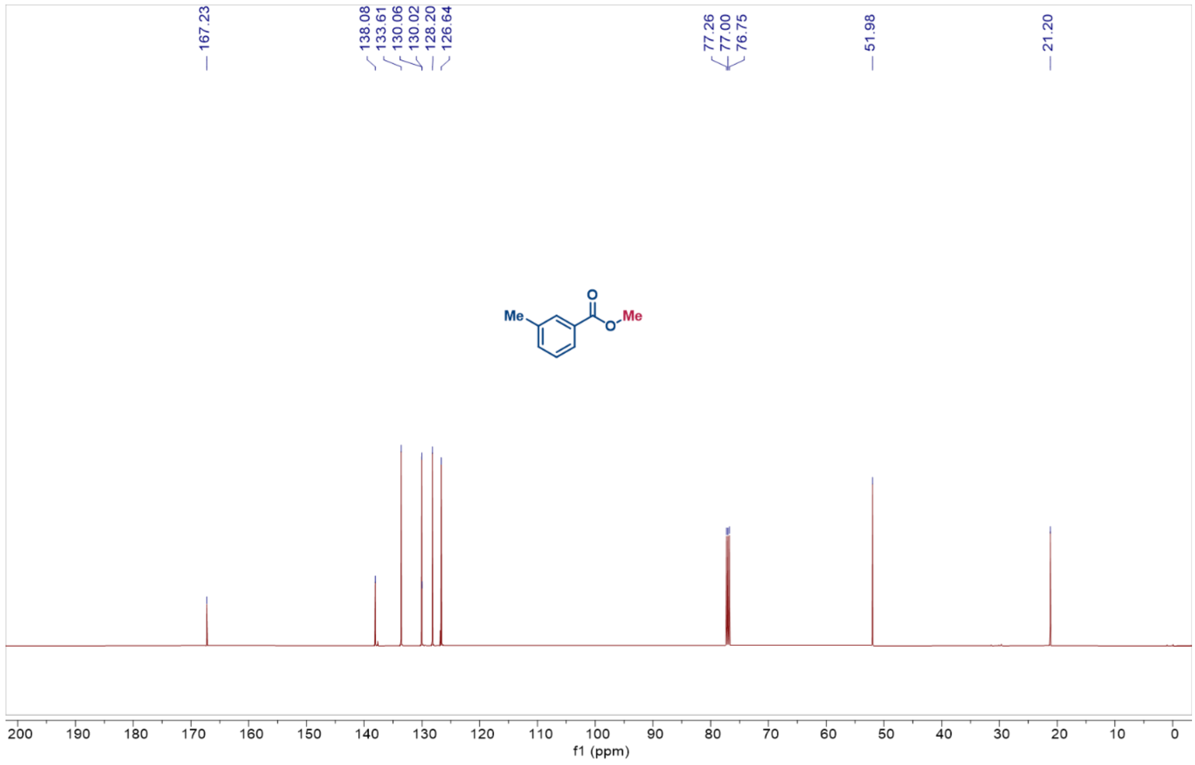

^{1}H NMR of **3e** (600 MHz, $CDCl_3$) and ^{13}C NMR of **3e** (151 MHz, $CDCl_3$)

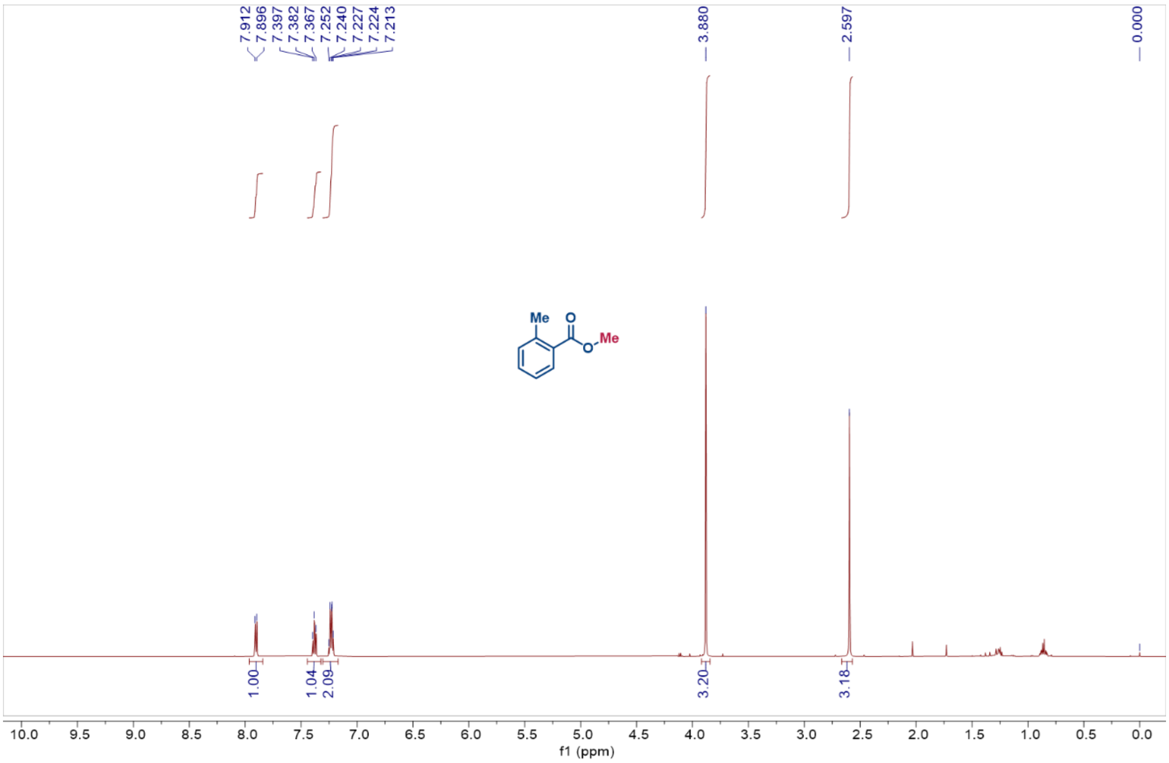

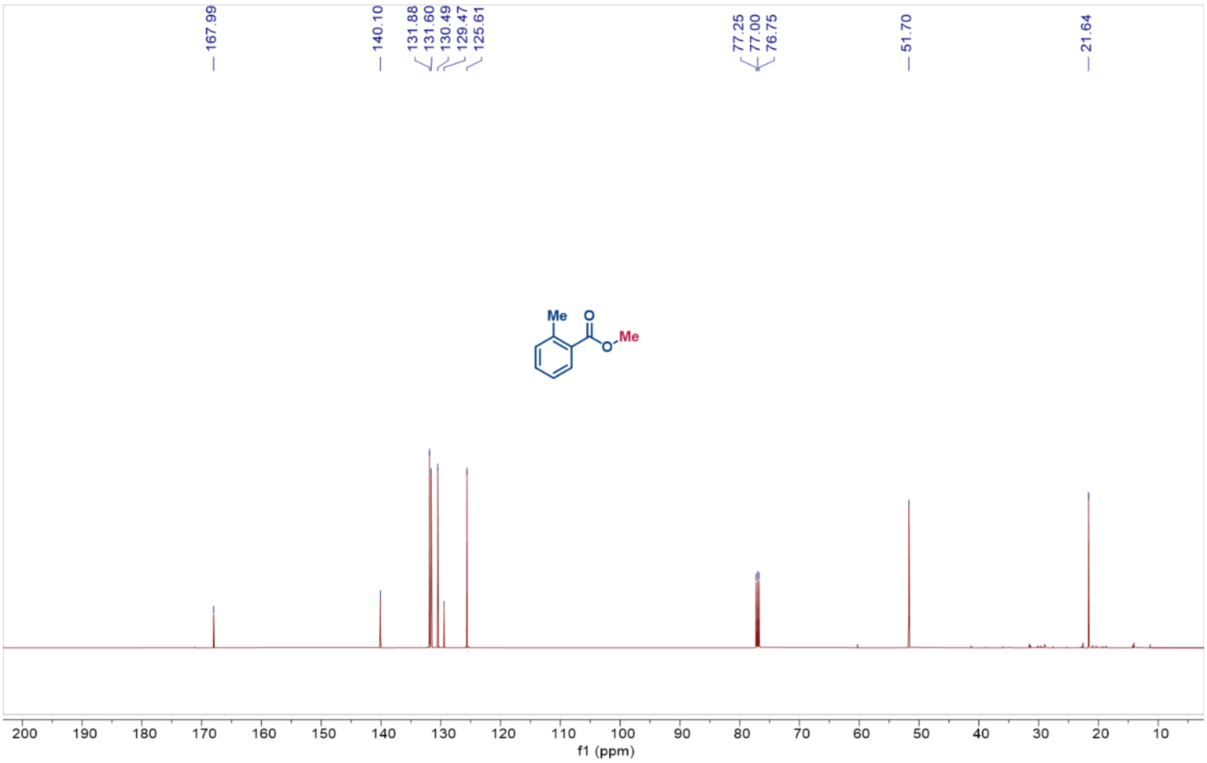

^{1}H NMR of **3f** (600 MHz, $CDCl_3$) and ^{13}C NMR of **3f** (151 MHz, $CDCl_3$)

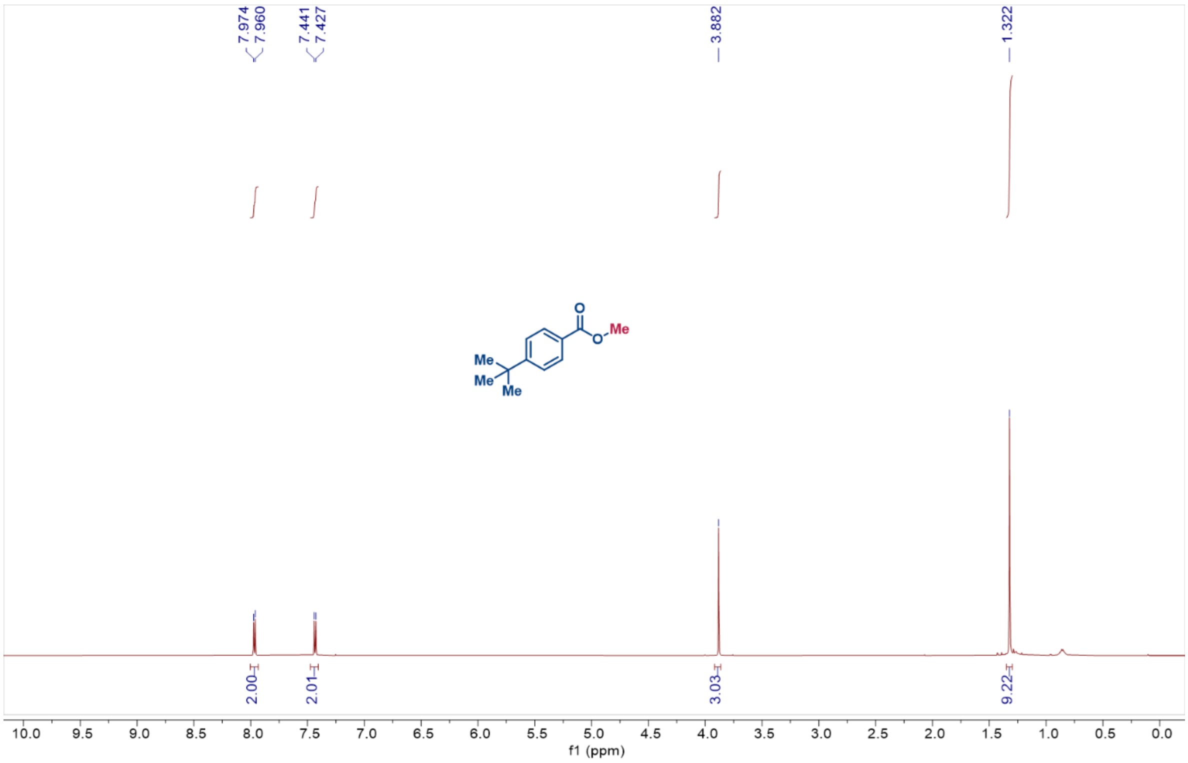

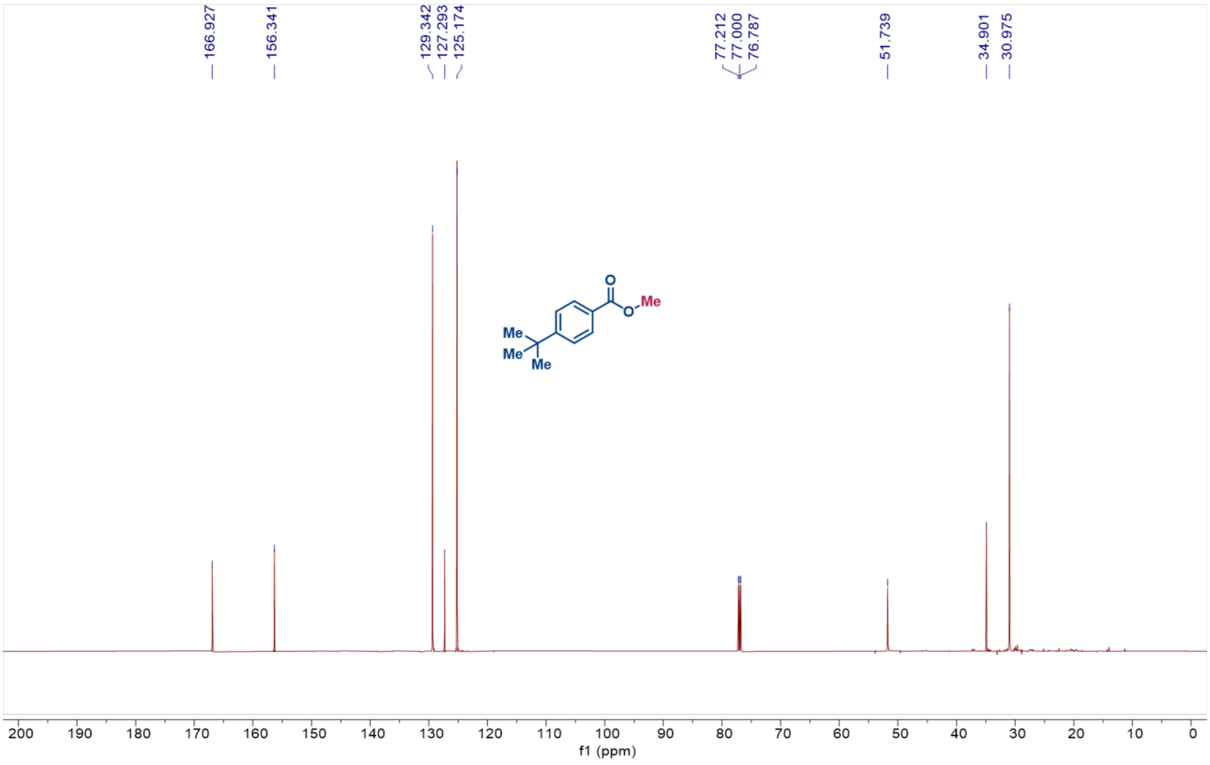

^{1}H NMR of **3g** (600 MHz, $CDCl_3$) and ^{13}C NMR of **3g** (151 MHz, $CDCl_3$)

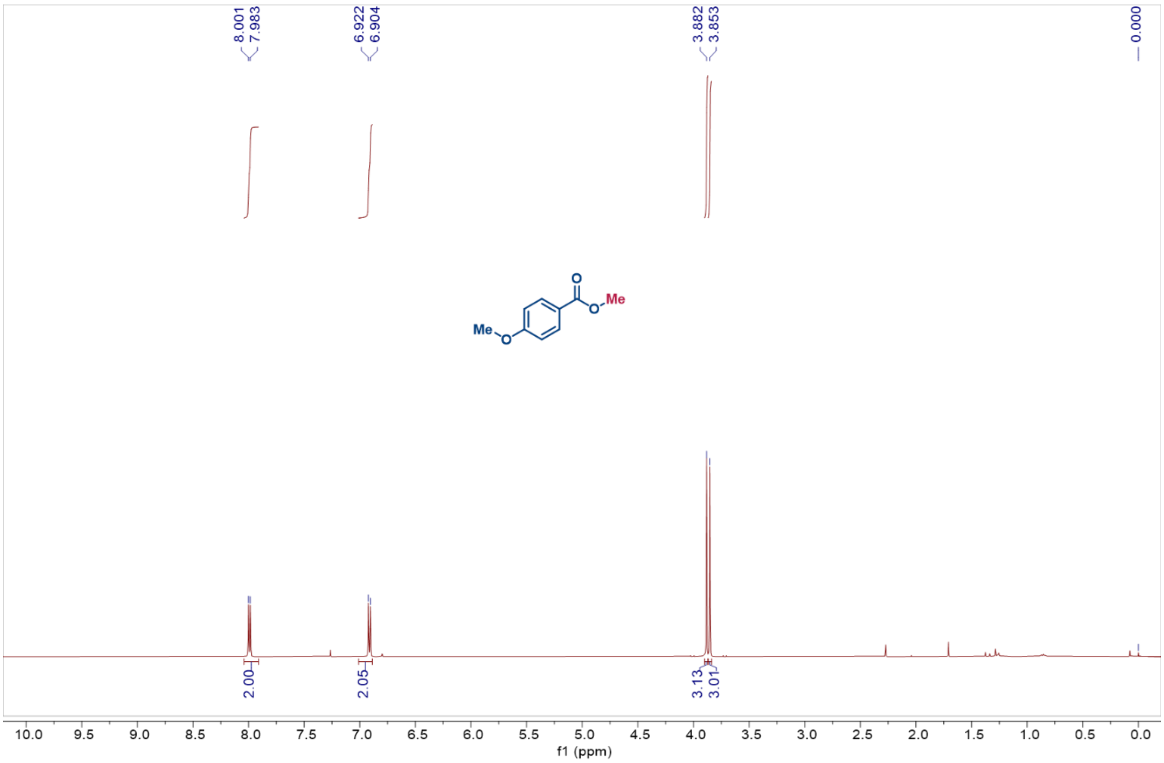

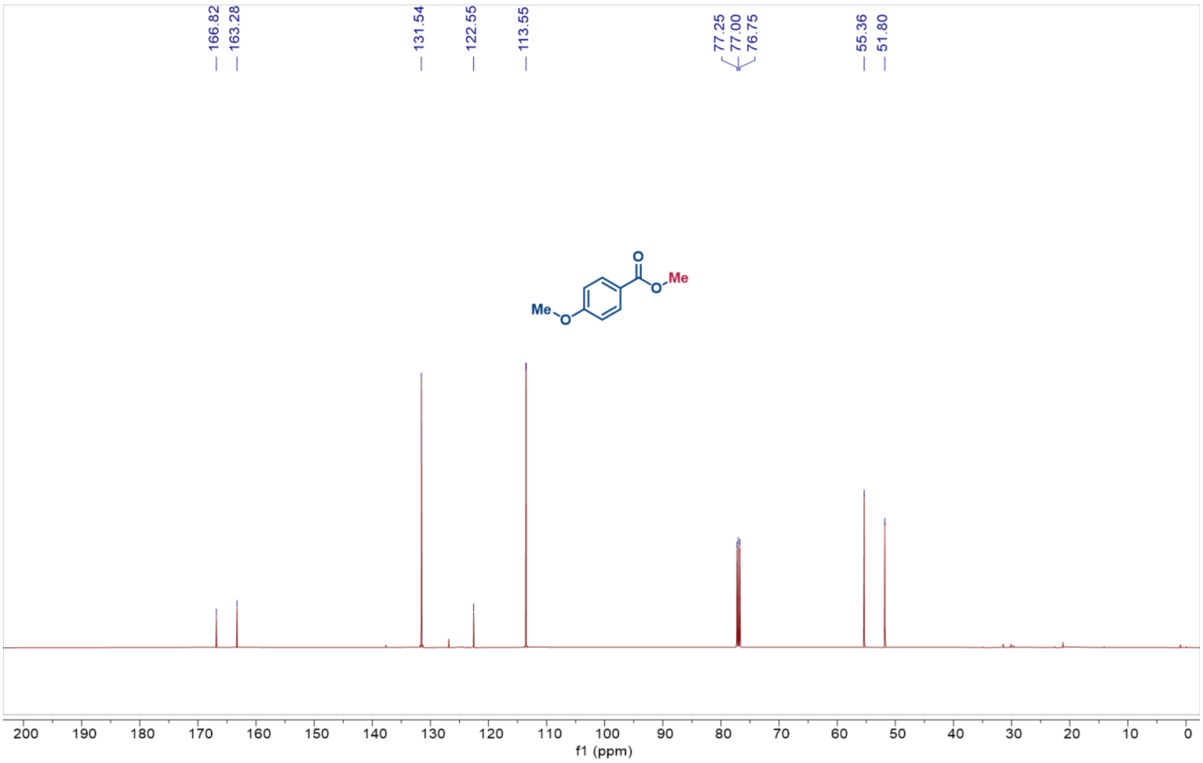

^{1}H NMR of **3h** (600 MHz, $CDCl_3$) and ^{13}C NMR of **3h** (151 MHz, $CDCl_3$)

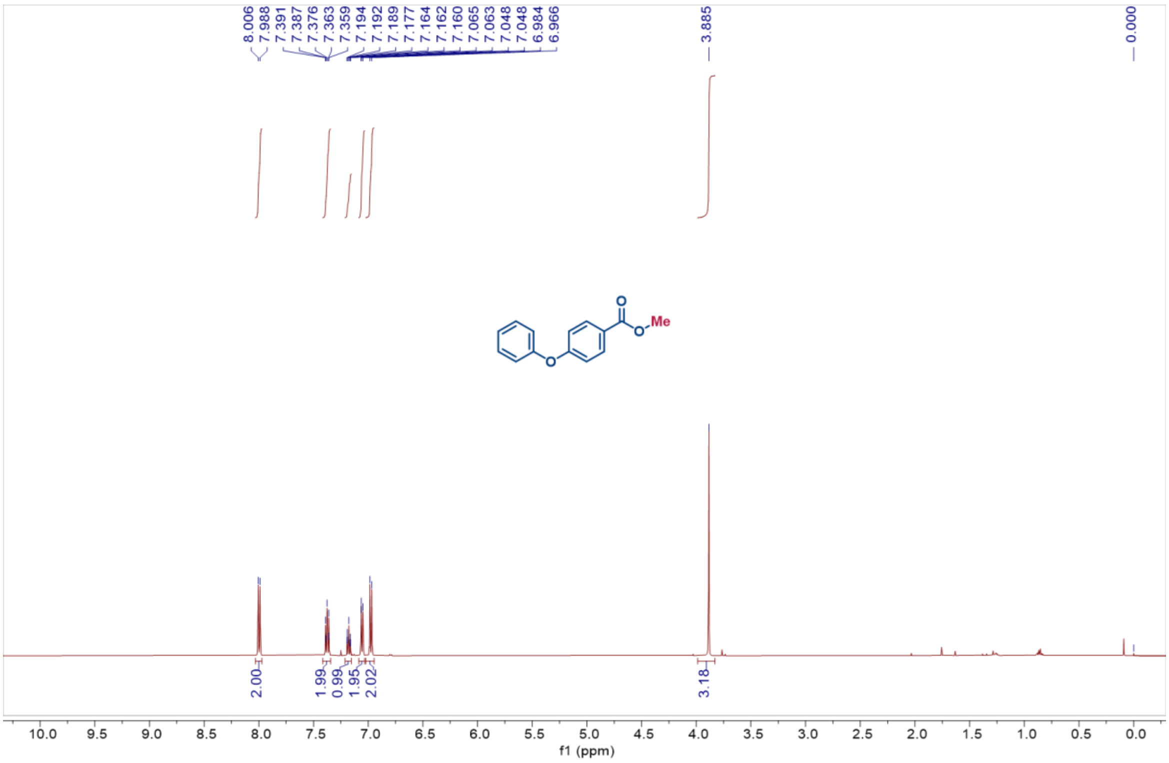

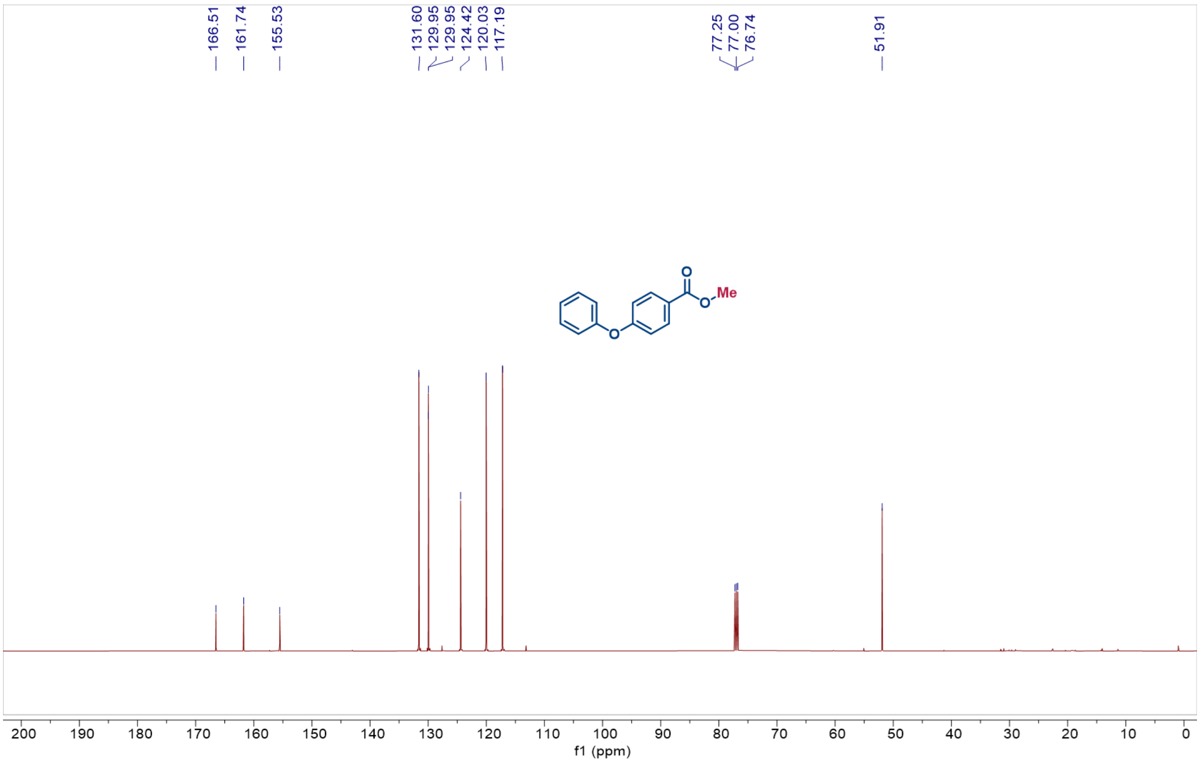

^{1}H NMR of **3i** (600 MHz, $CDCl_3$) and ^{13}C NMR of **3i** (151 MHz, $CDCl_3$)

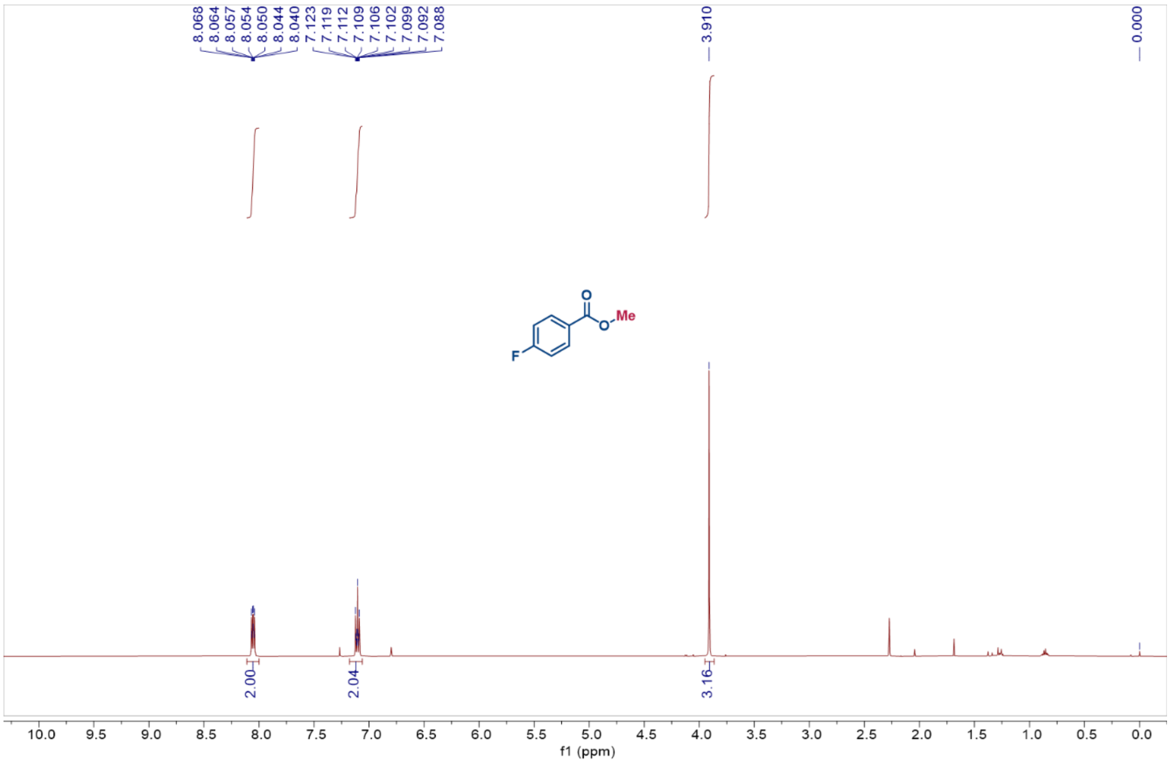

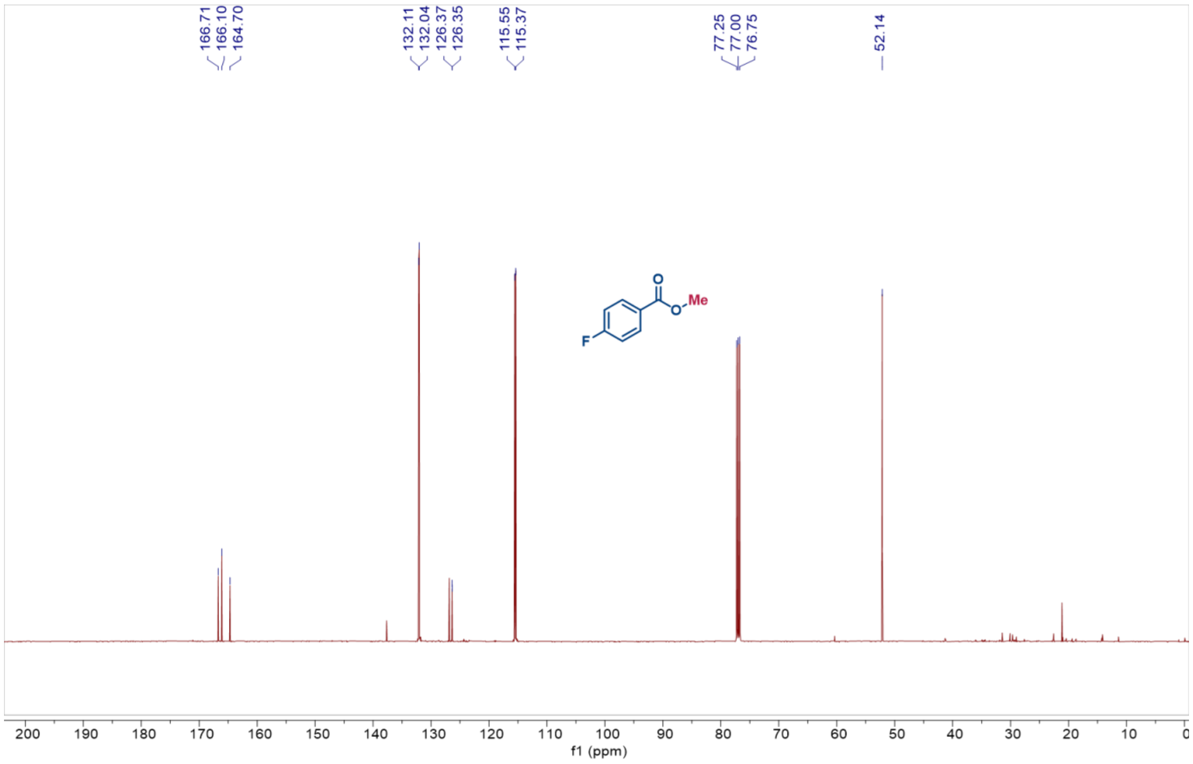

^{1}H NMR of **3j** (600 MHz, $CDCl_3$) and ^{13}C NMR of **3j** (151 MHz, $CDCl_3$)

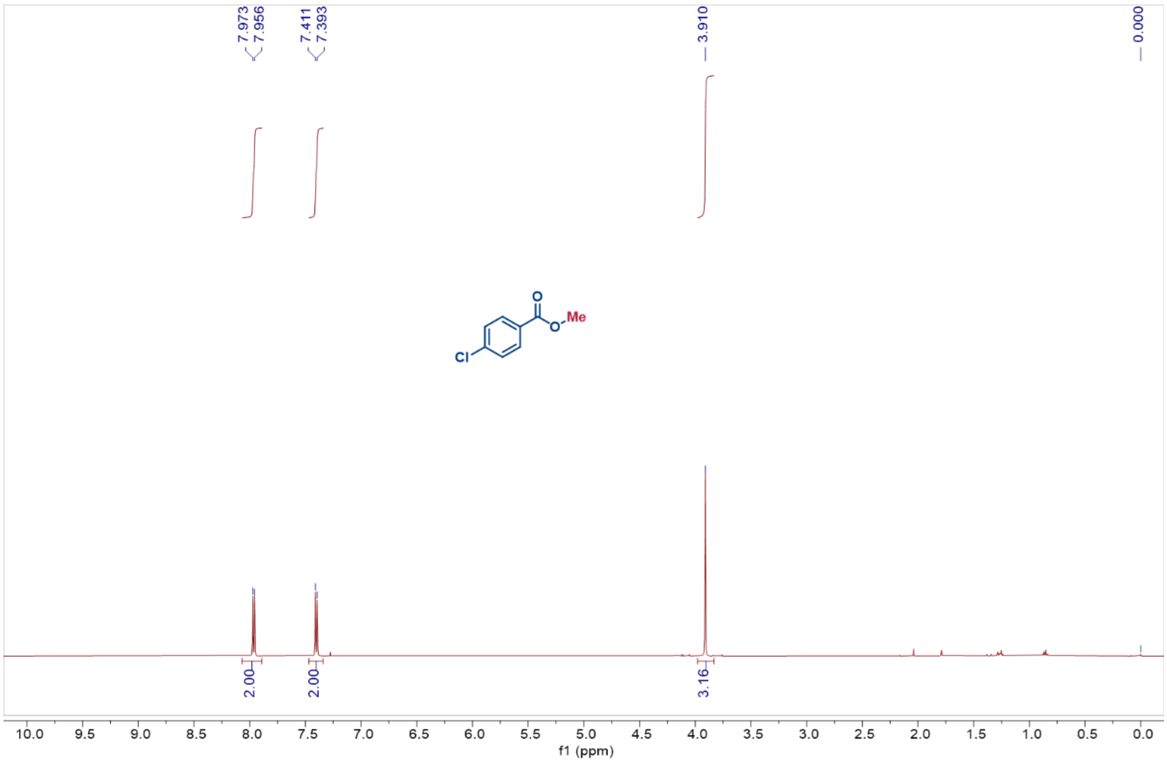

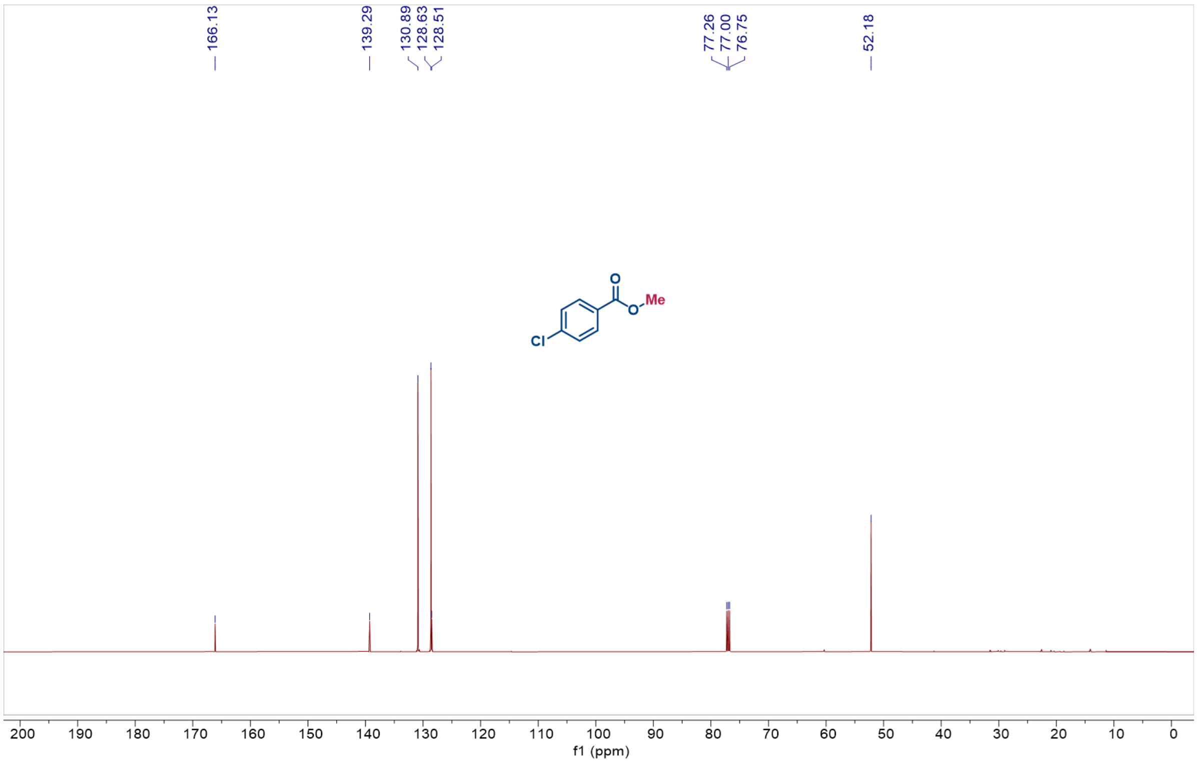

^{1}H NMR of **3k** (600 MHz, $CDCl_3$) and ^{13}C NMR of **3k** (151 MHz, $CDCl_3$)

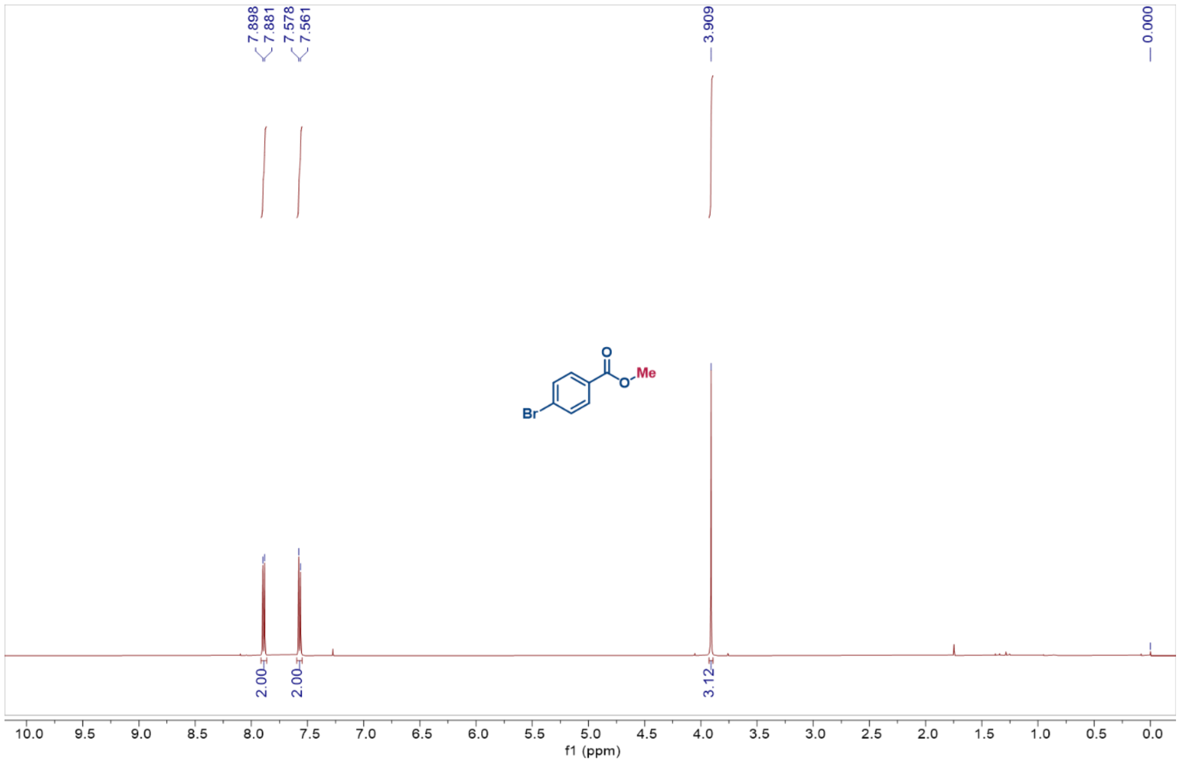

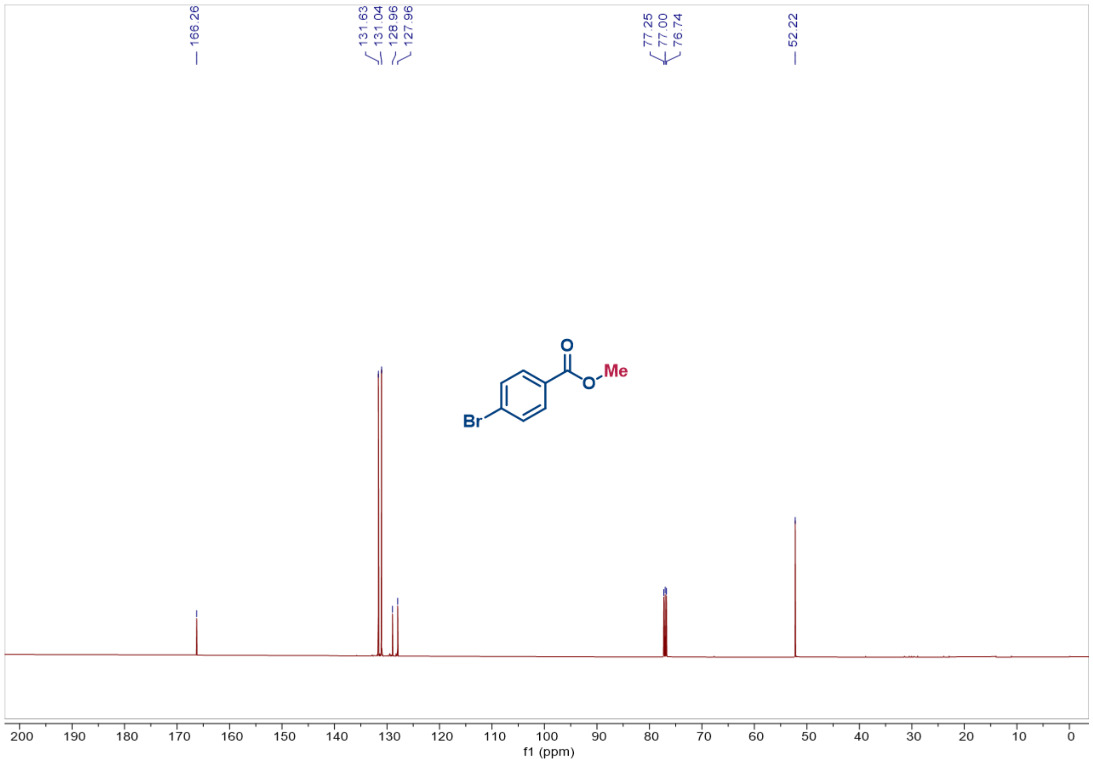

^{1}H NMR of **3l** (600 MHz, $CDCl_3$) and ^{13}C NMR of **3l** (151 MHz, $CDCl_3$)

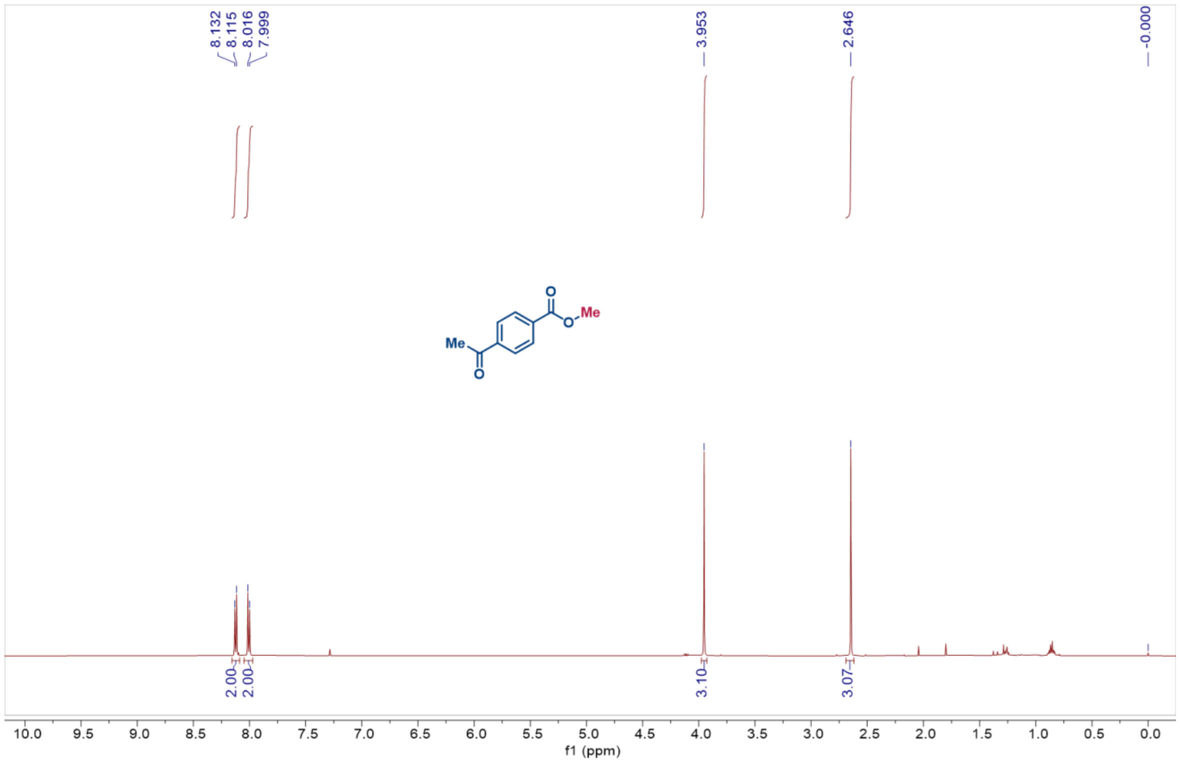

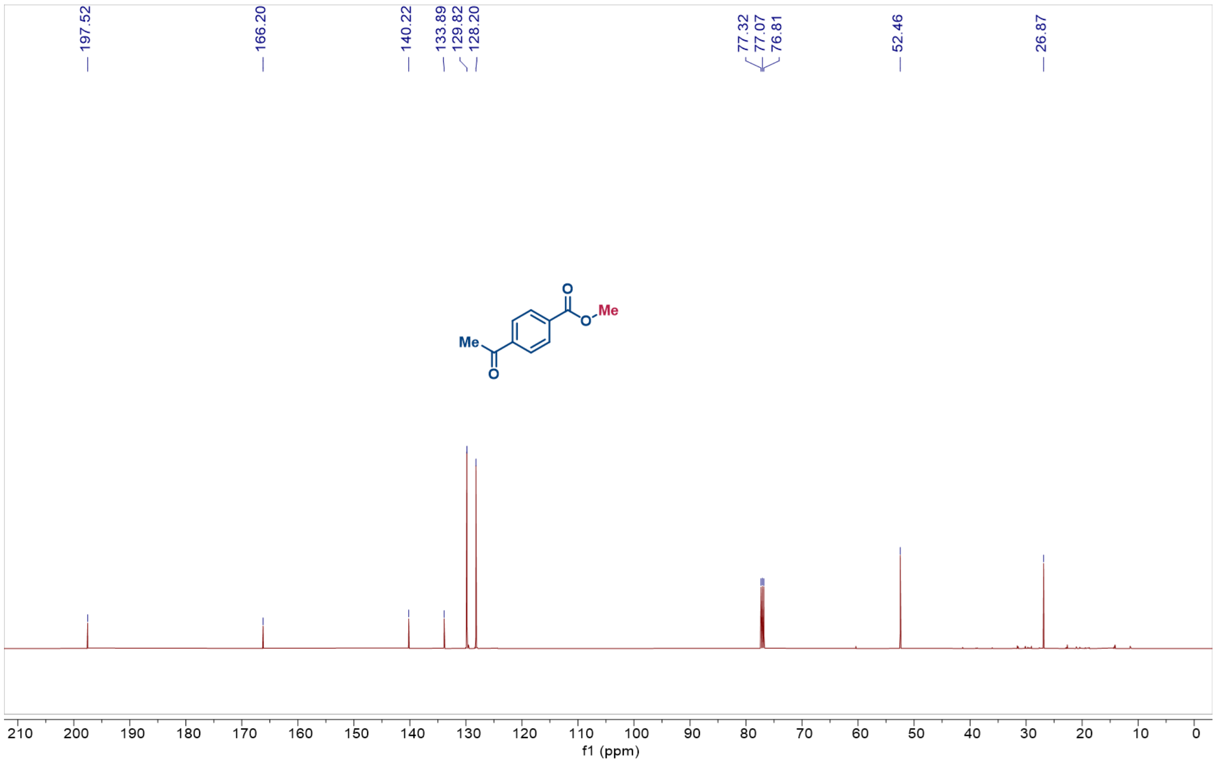

^{1}H NMR of **3m** (600 MHz, $CDCl_3$) and ^{13}C NMR of **3m** (151 MHz, $CDCl_3$)

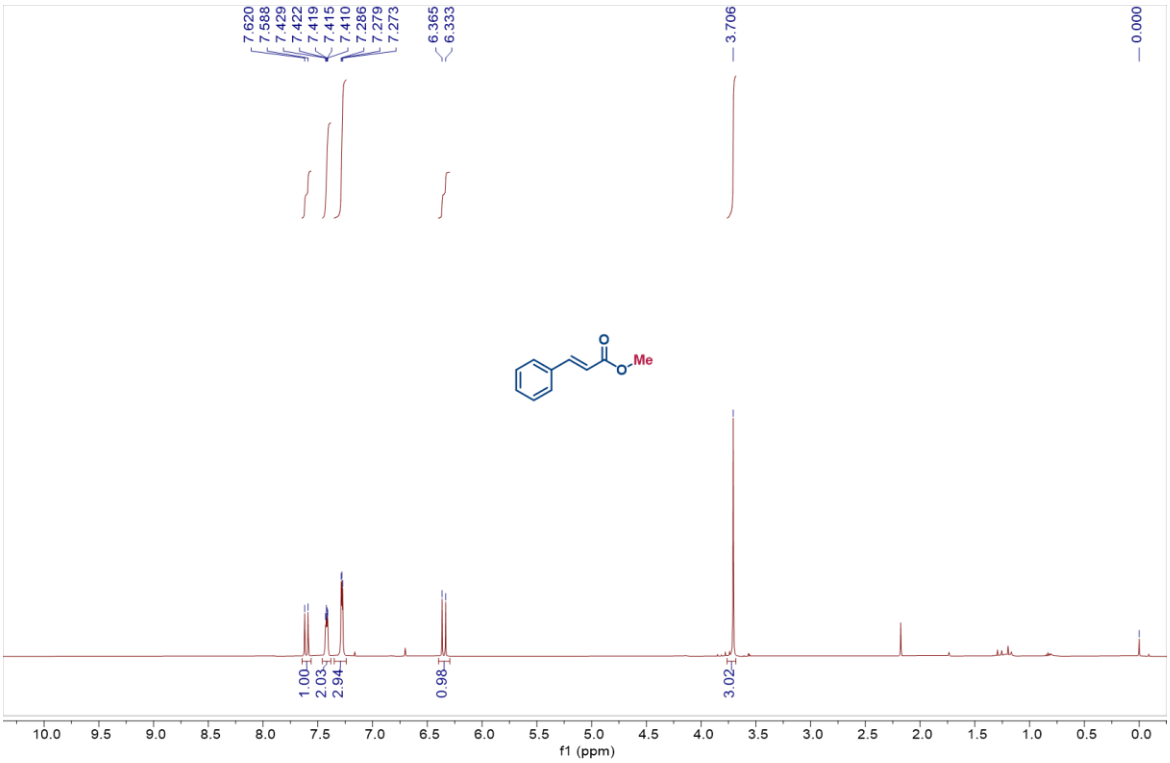

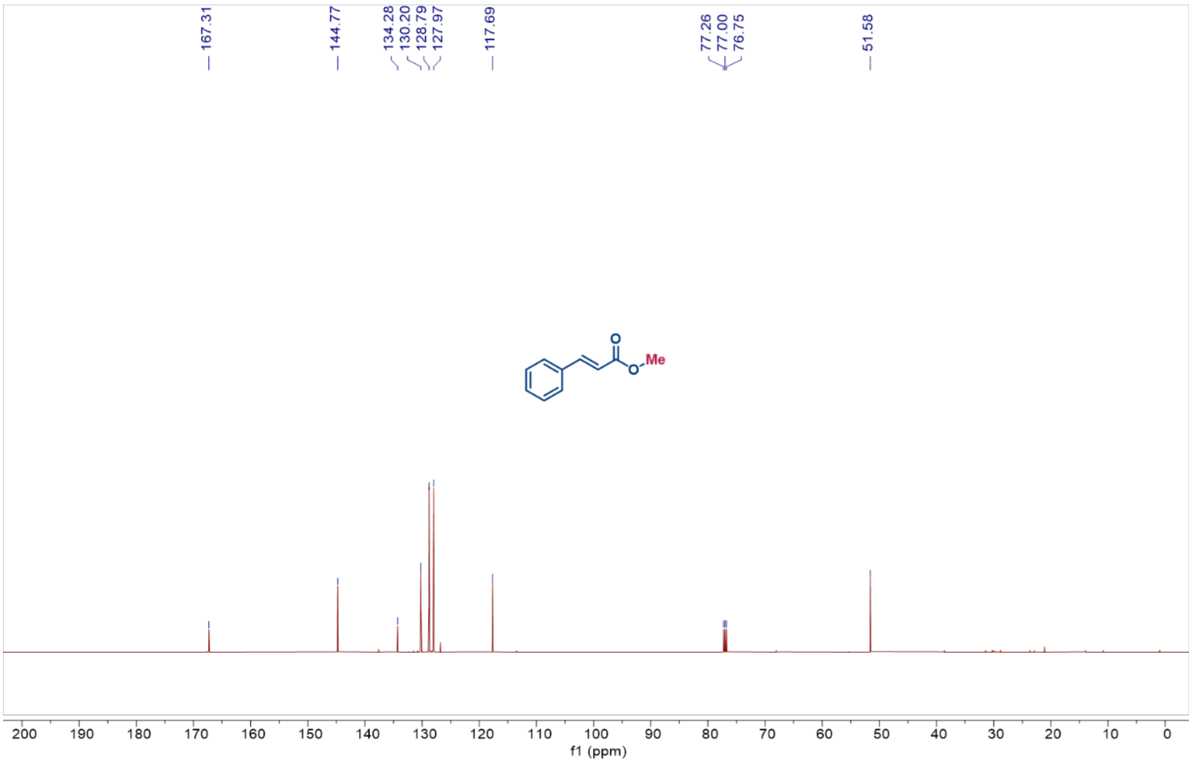

^{1}H NMR of **3n** (600 MHz, $CDCl_3$) and ^{13}C NMR of **3n** (151 MHz, $CDCl_3$)

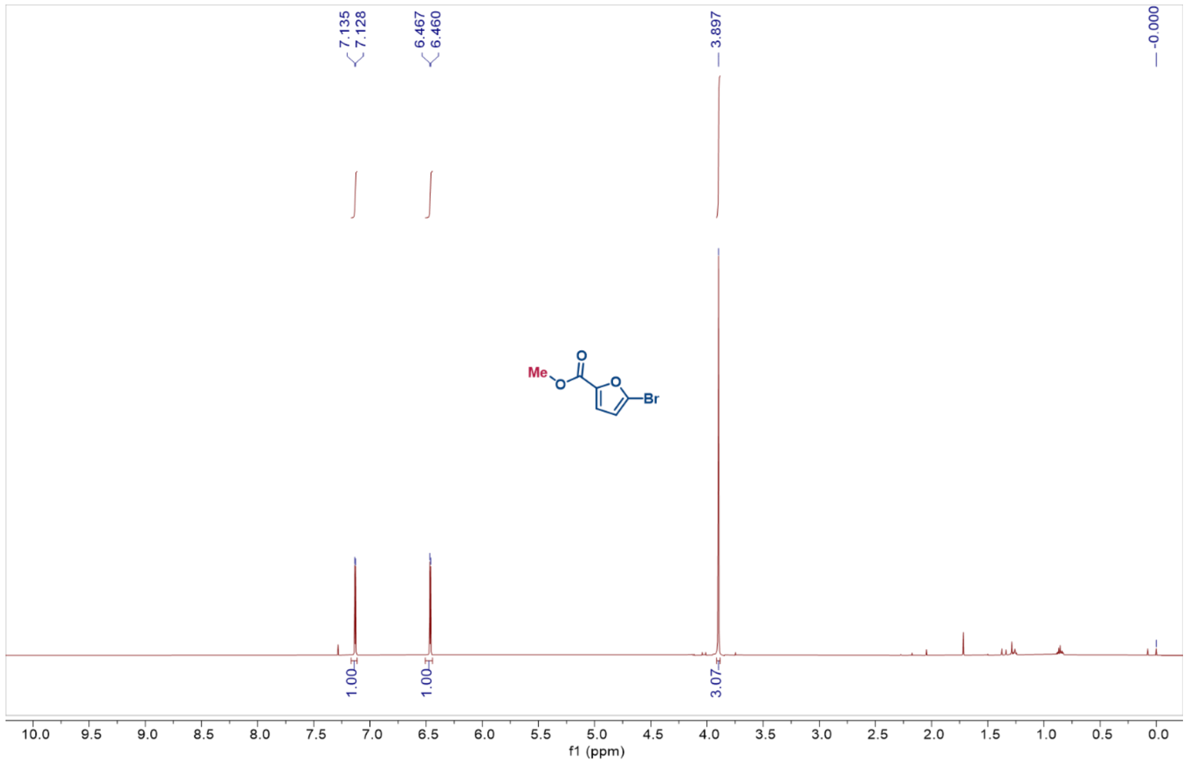

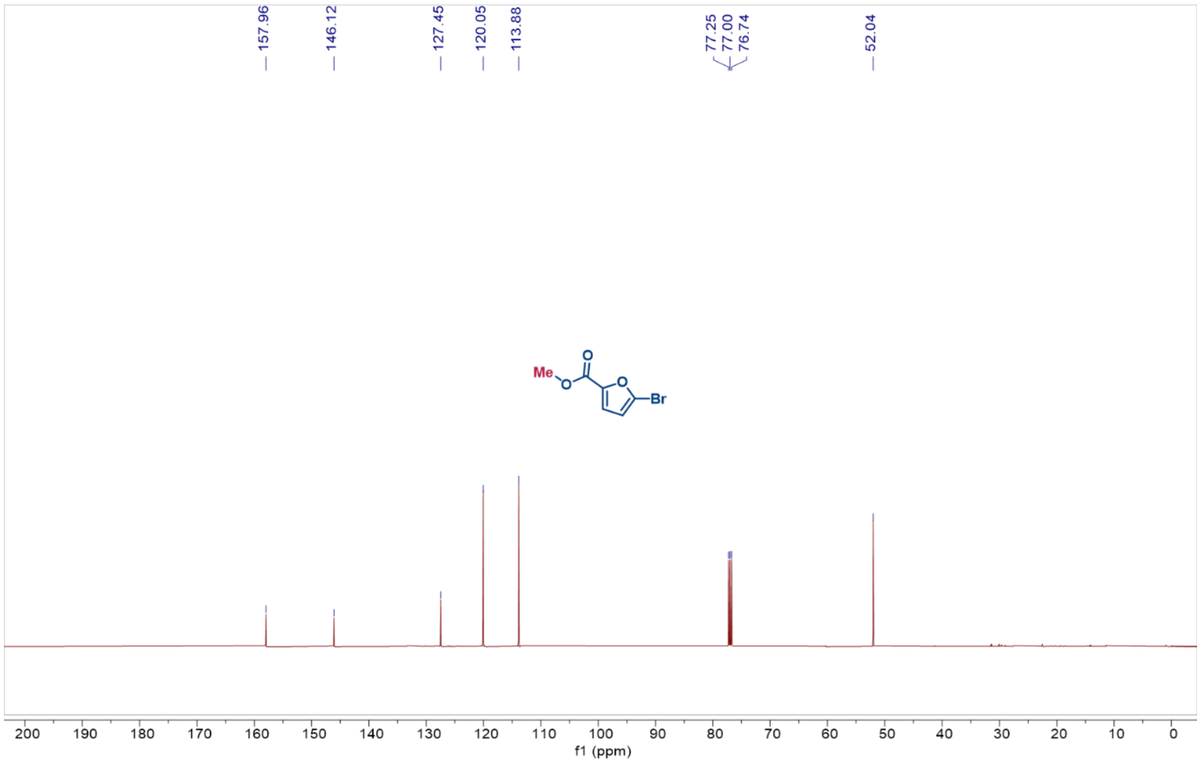

^{1}H NMR of **3o** (600 MHz, $CDCl_3$) and ^{13}C NMR of **3o** (151 MHz, $CDCl_3$)

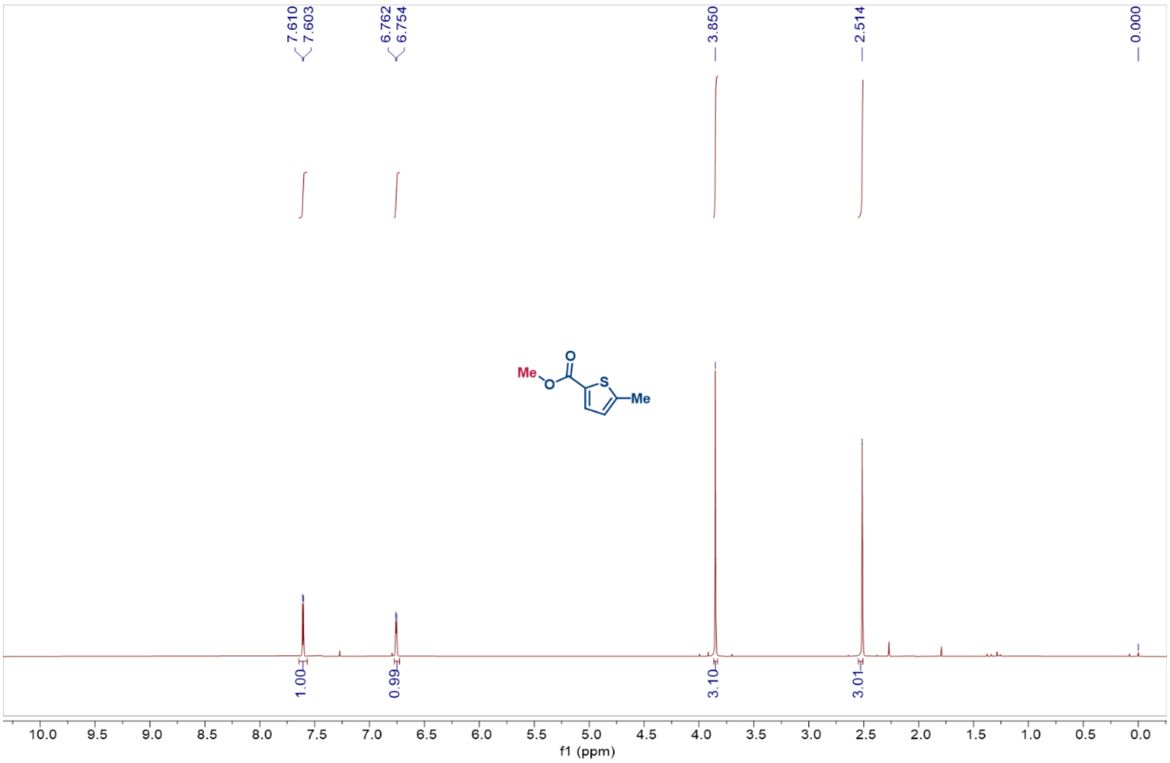

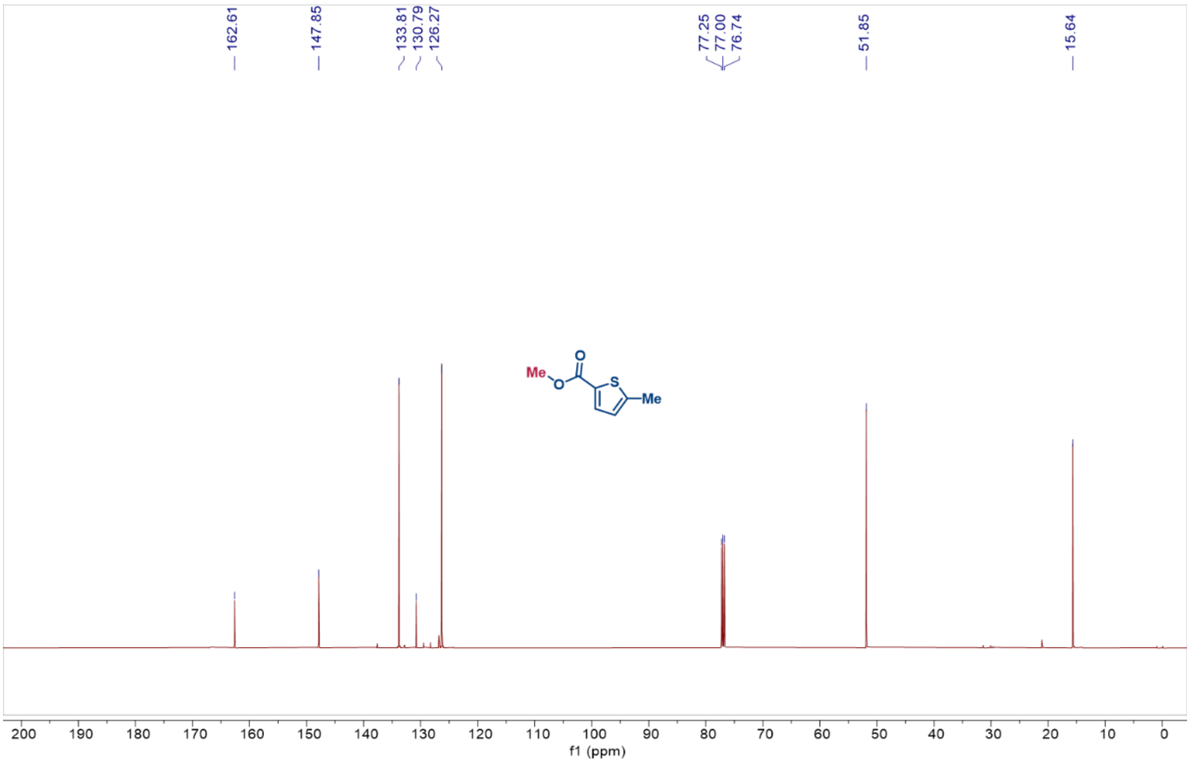

^{1}H NMR of **3p** (600 MHz, $CDCl_3$) and ^{13}C NMR of **3p** (151 MHz, $CDCl_3$)

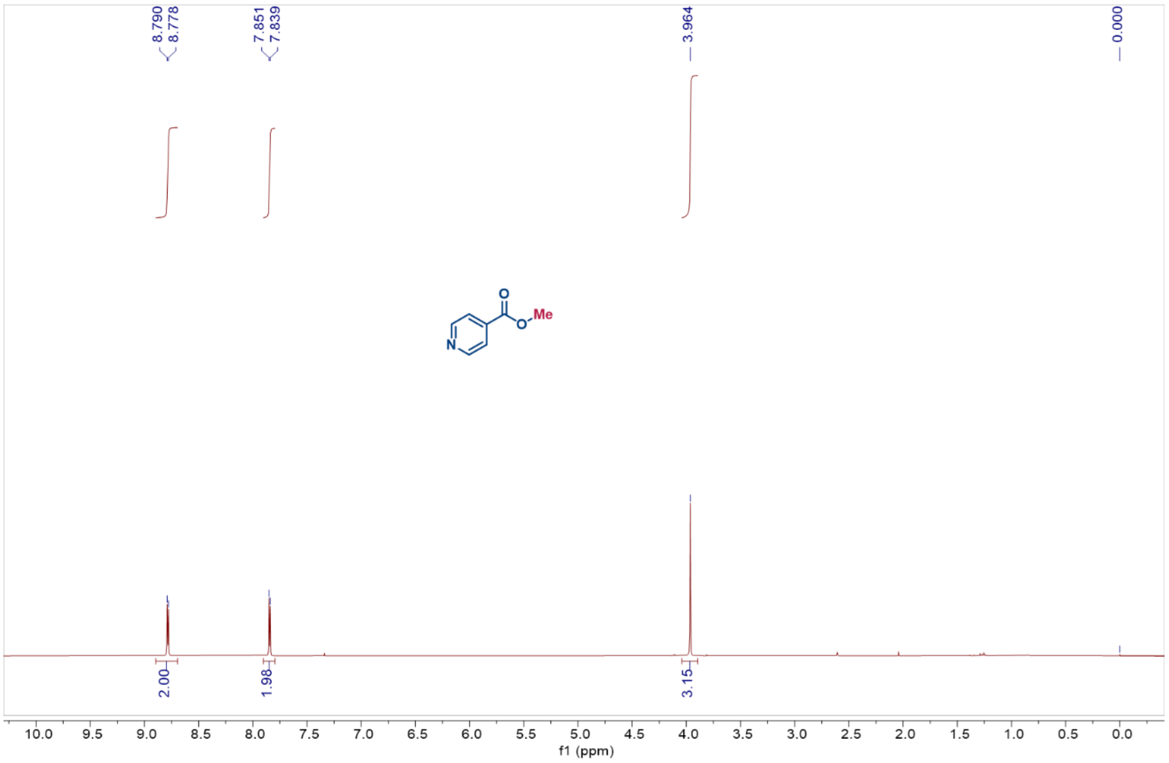

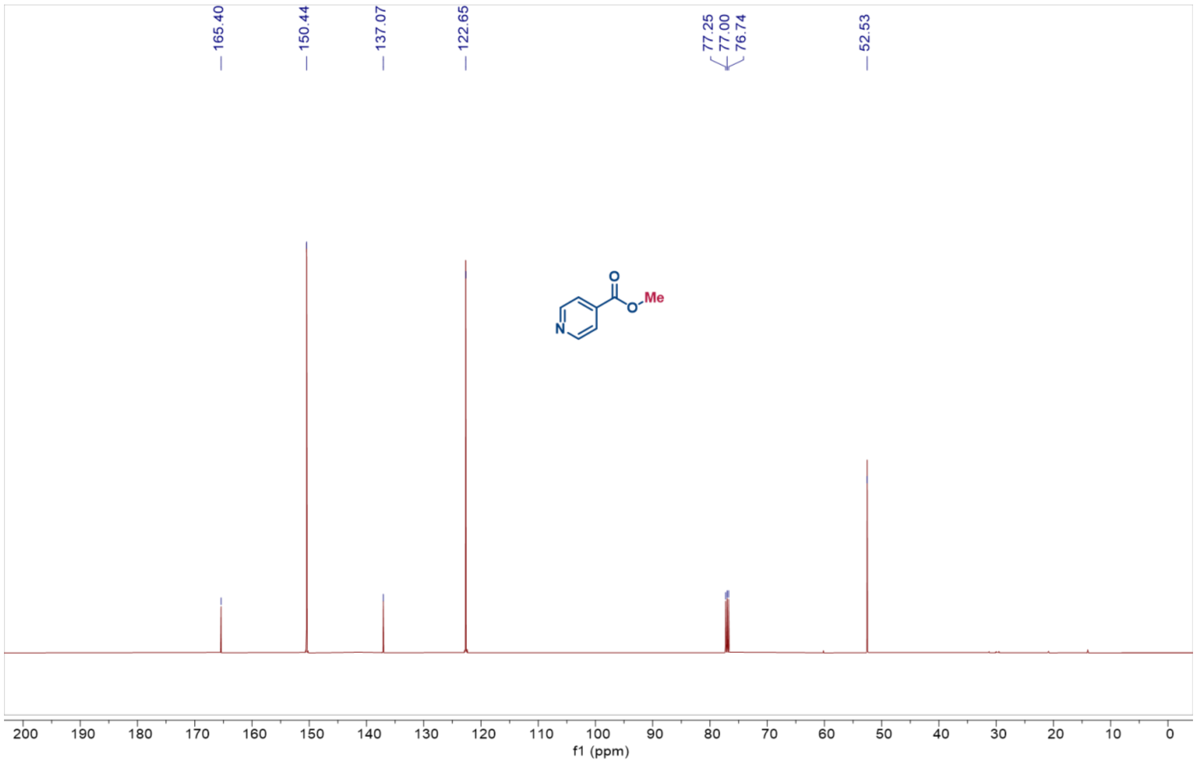

^{1}H NMR of **3q** (600 MHz, $CDCl_3$) and ^{13}C NMR of **3q** (151 MHz, $CDCl_3$)

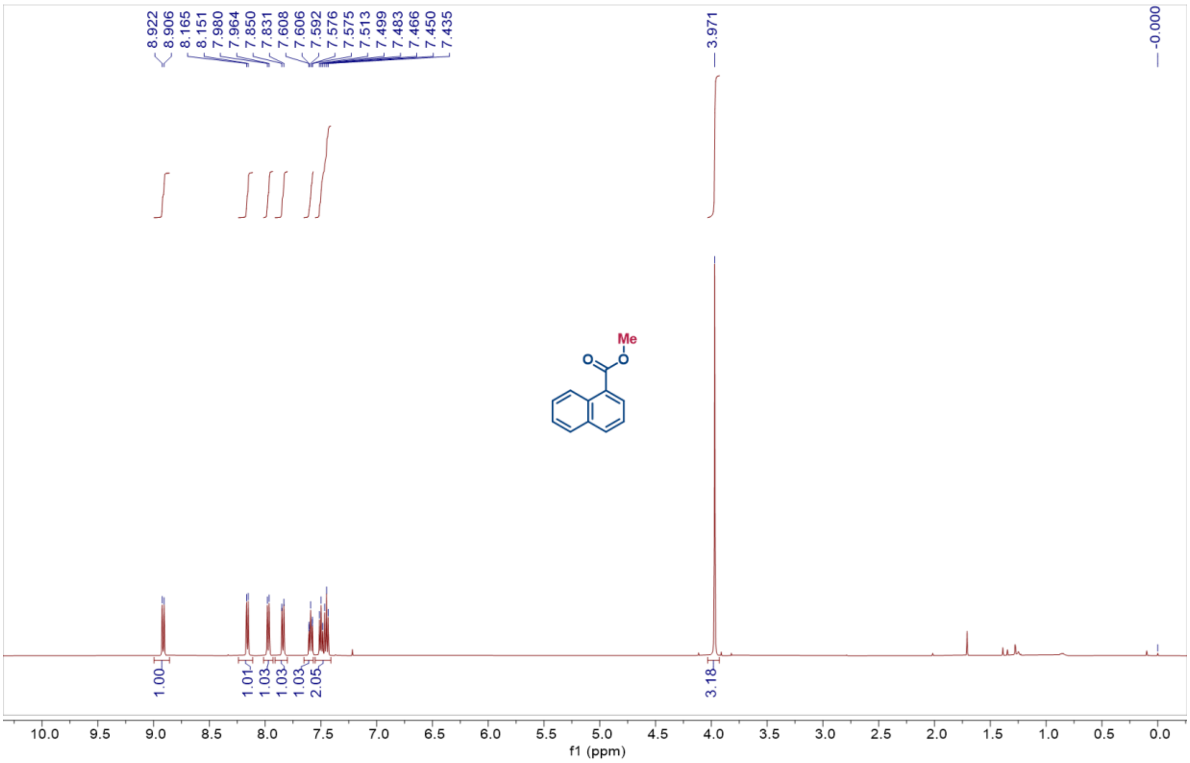

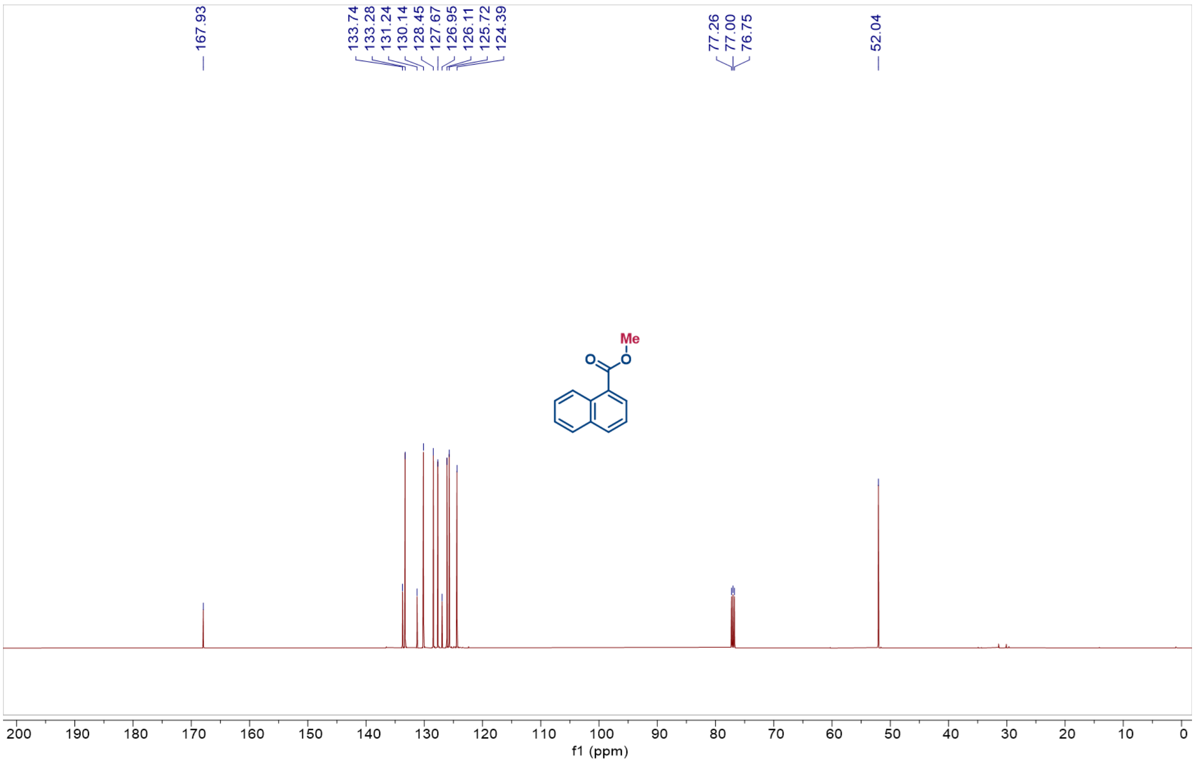

^{1}H NMR of **3r** (600 MHz, $CDCl_3$) and ^{13}C NMR of **3r** (151 MHz, $CDCl_3$)

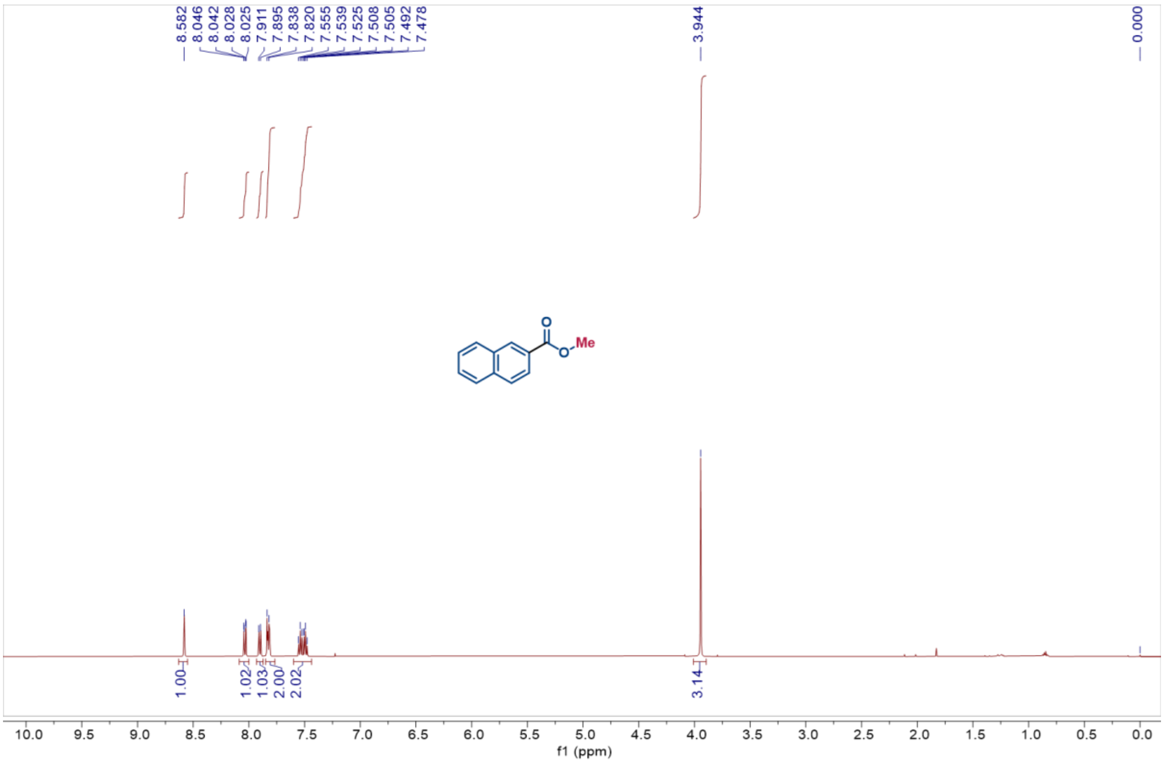

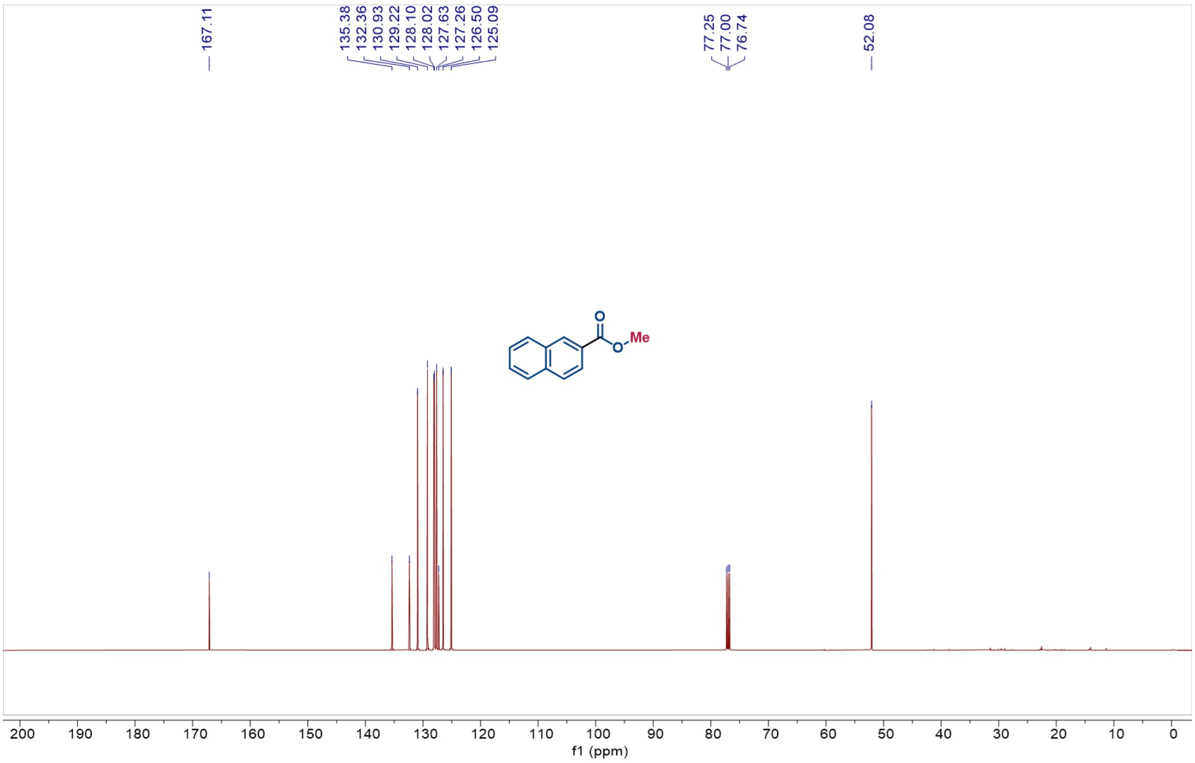

^{1}H NMR of **3s** (600 MHz, $CDCl_3$) and ^{13}C NMR of **3s** (151 MHz, $CDCl_3$)

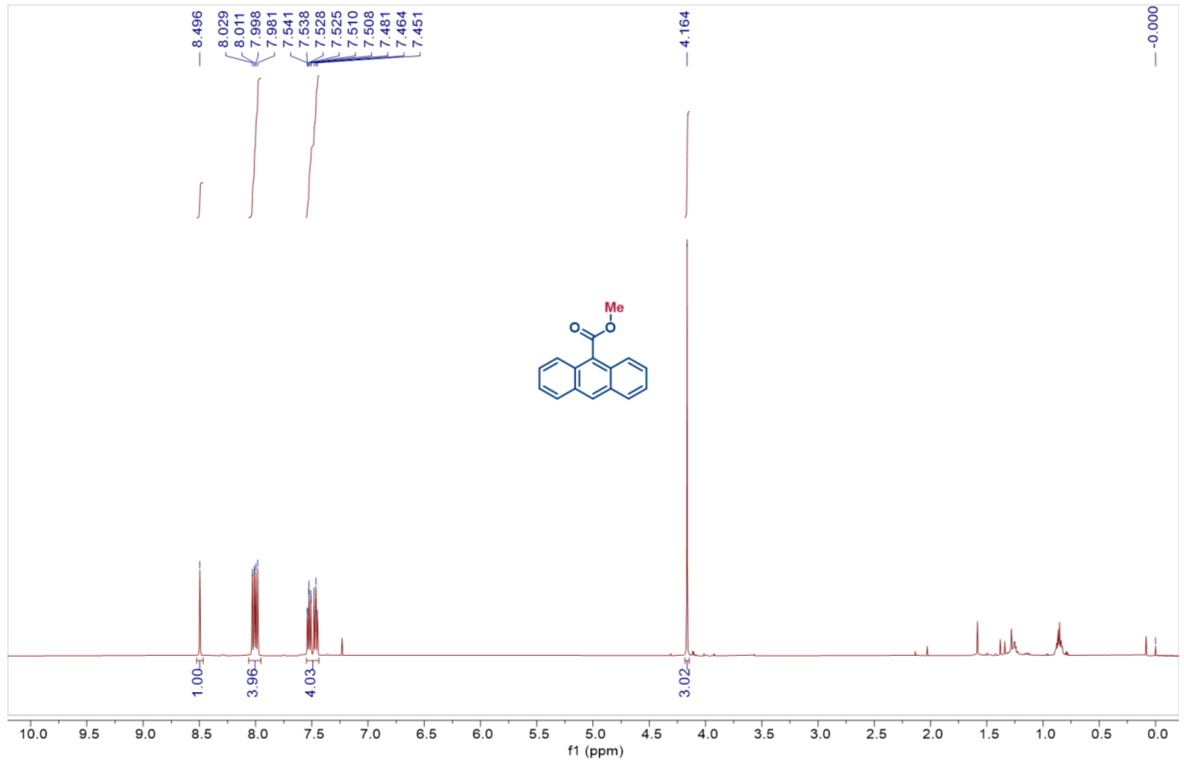

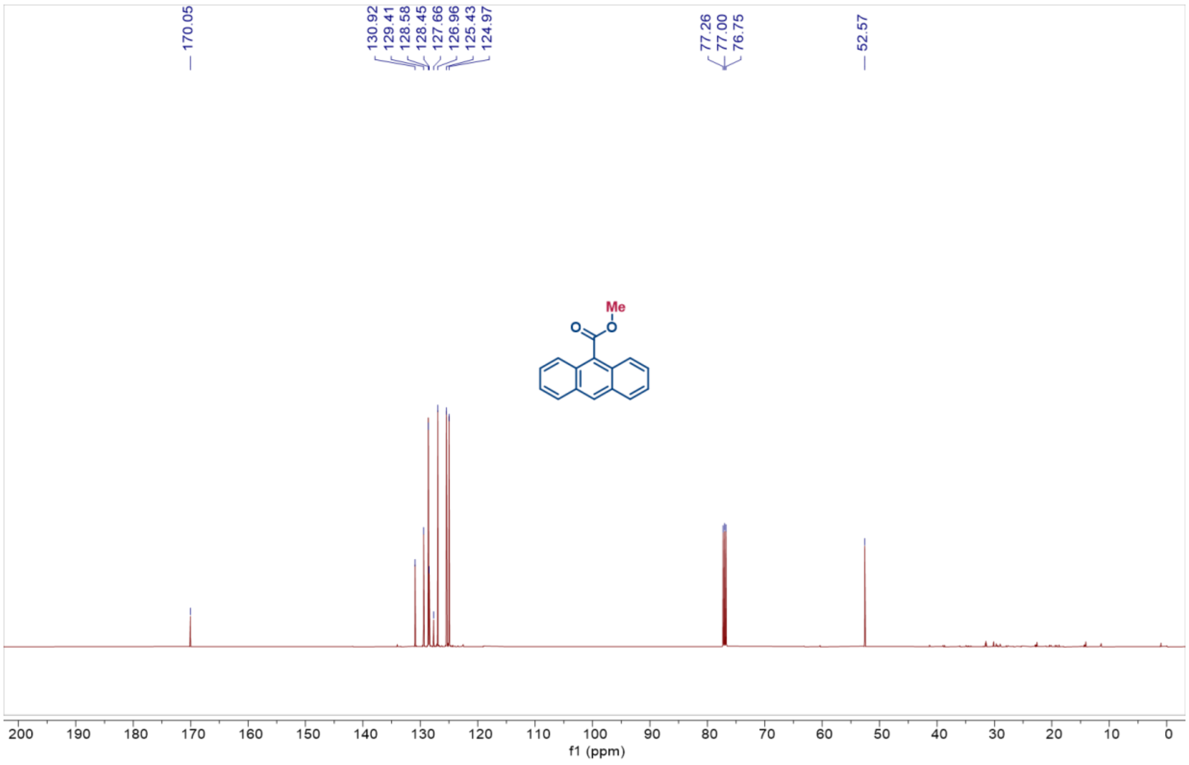

^{1}H NMR of **3t** (600 MHz, $CDCl_3$) and ^{13}C NMR of **3t** (151 MHz, $CDCl_3$)

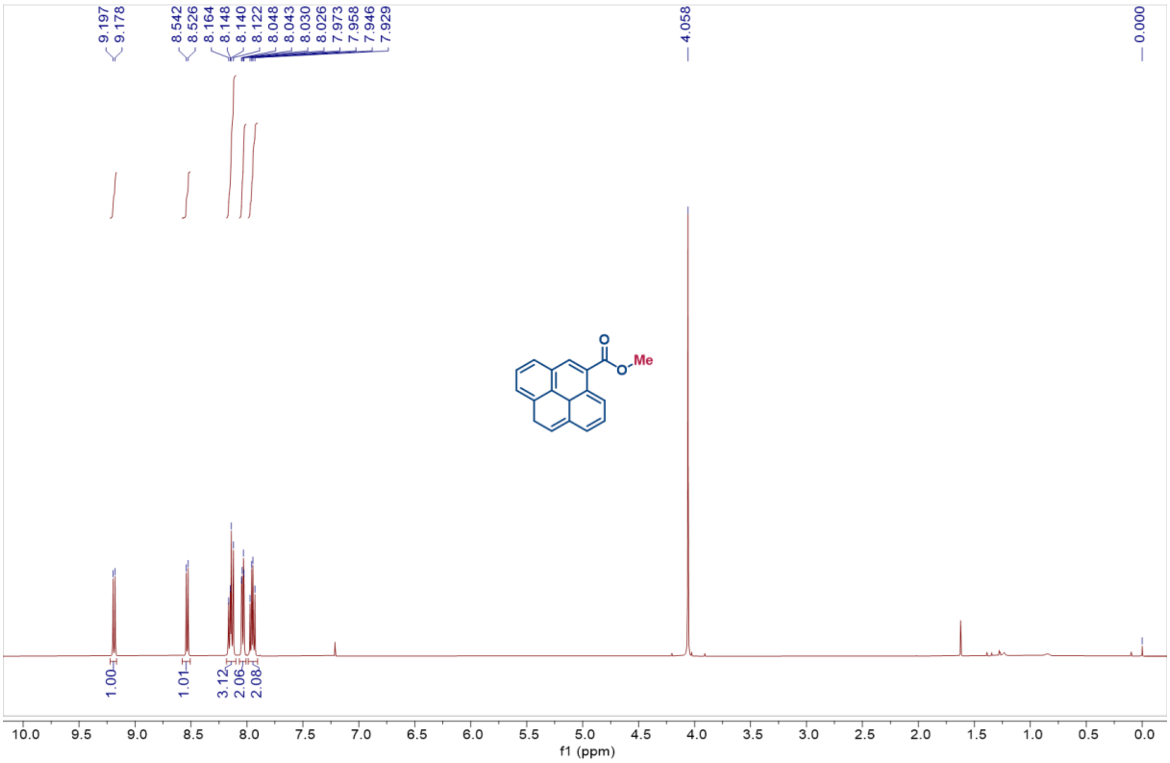

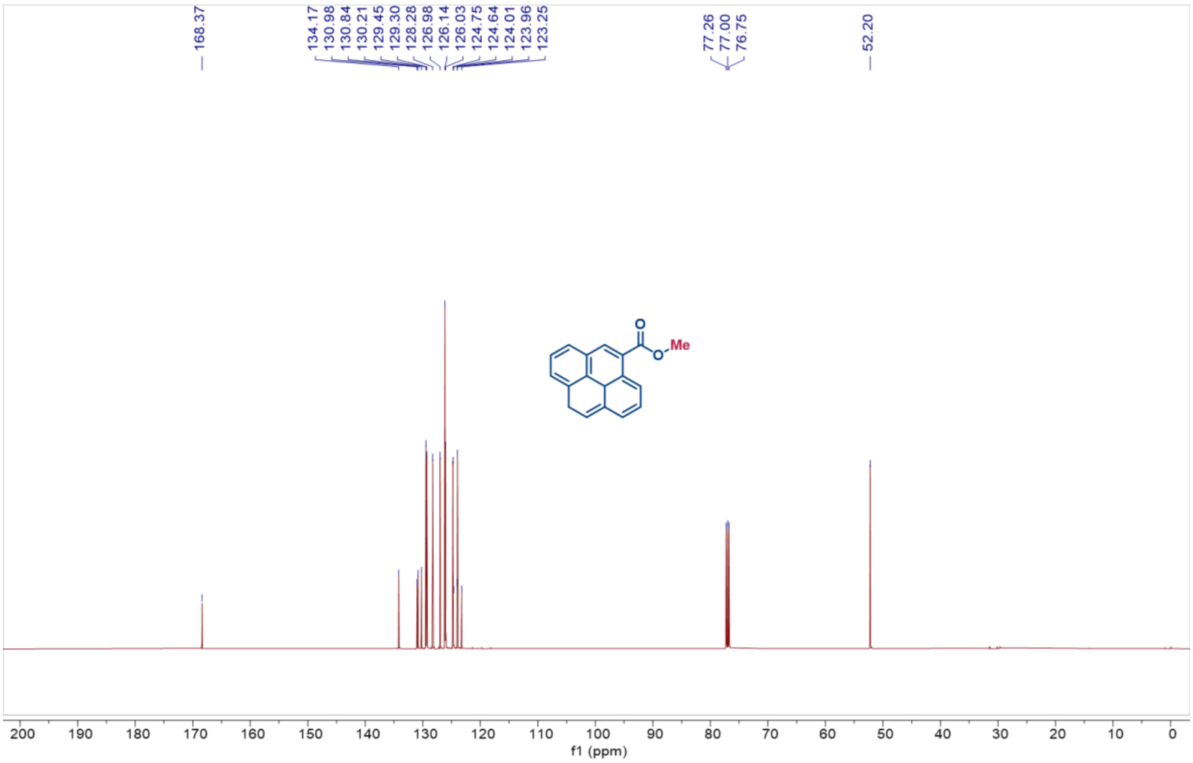

11. LC-MS spectra of part EG-derived product

Products derived from EG units using dimethyl malonate as a transesterification reagent

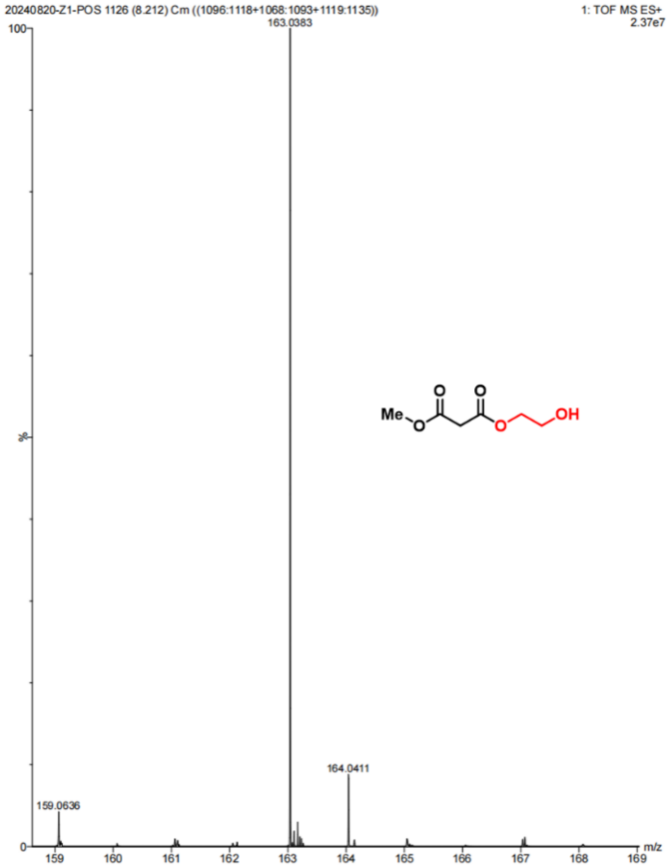

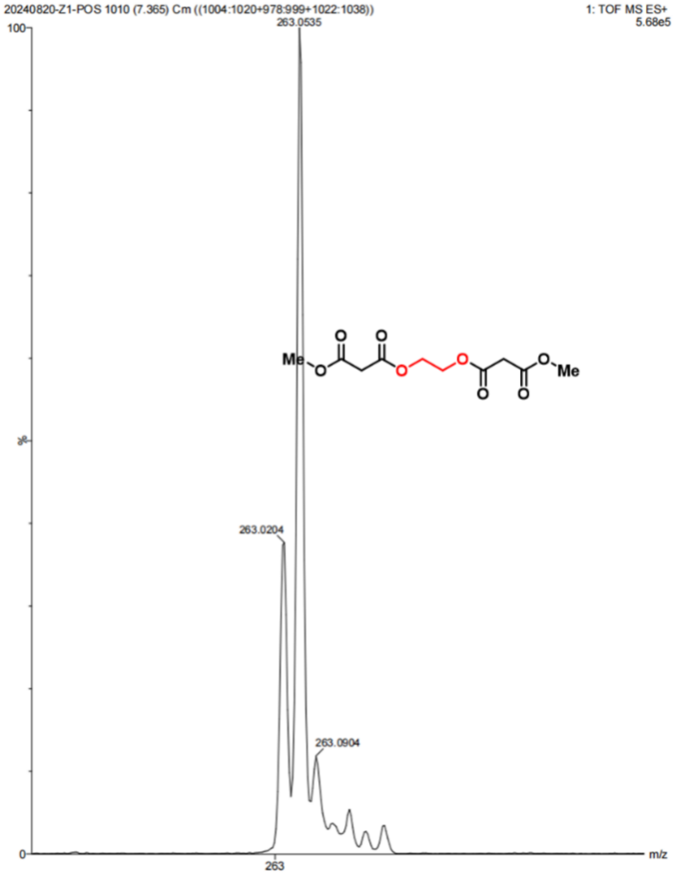

Products derived from EG units using methyl 2-oxo-2-phenylacetate as a transesterification reagent

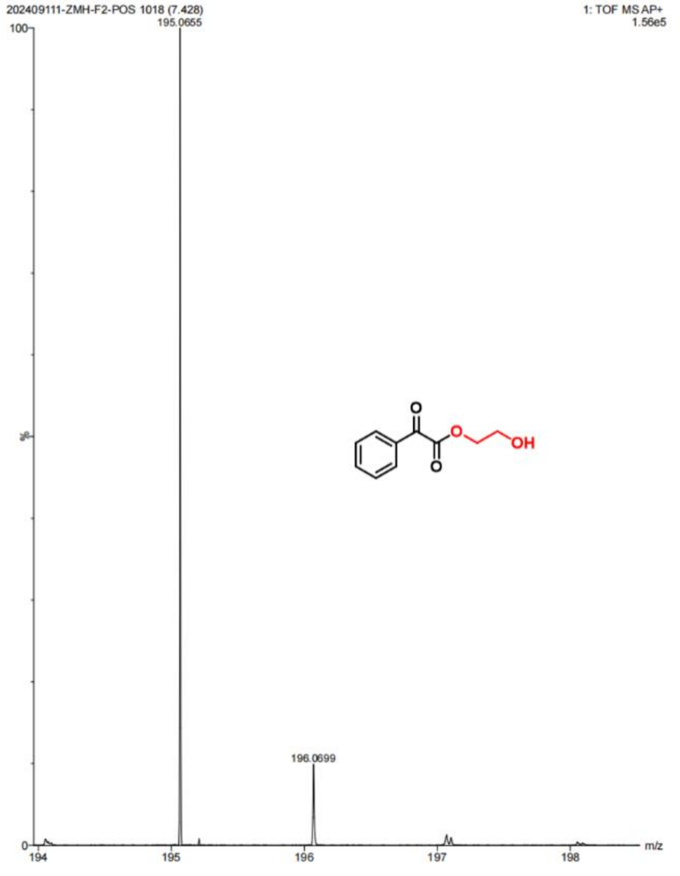

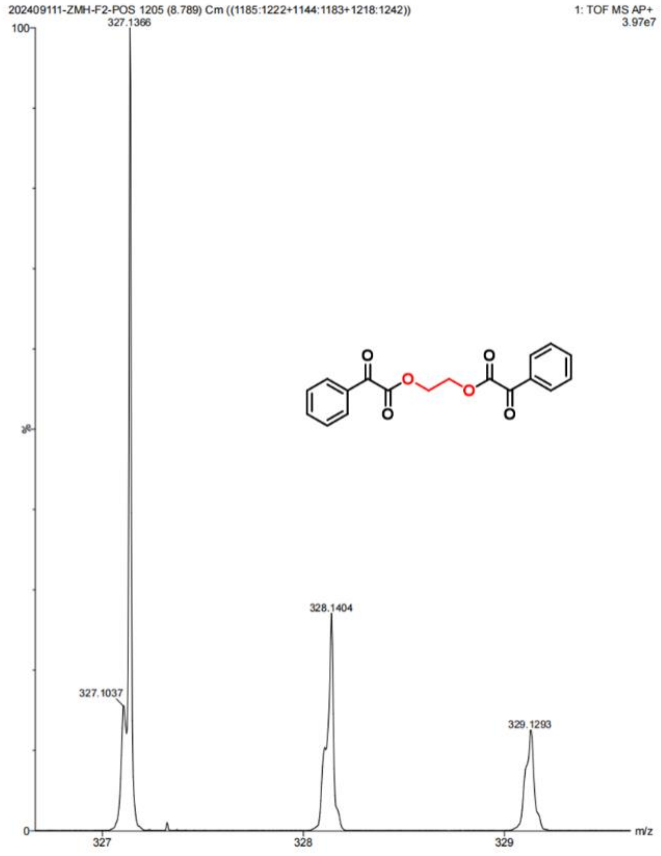

Products derived from EG units using methyl 3-oxobutanoate as a transesterification reagent

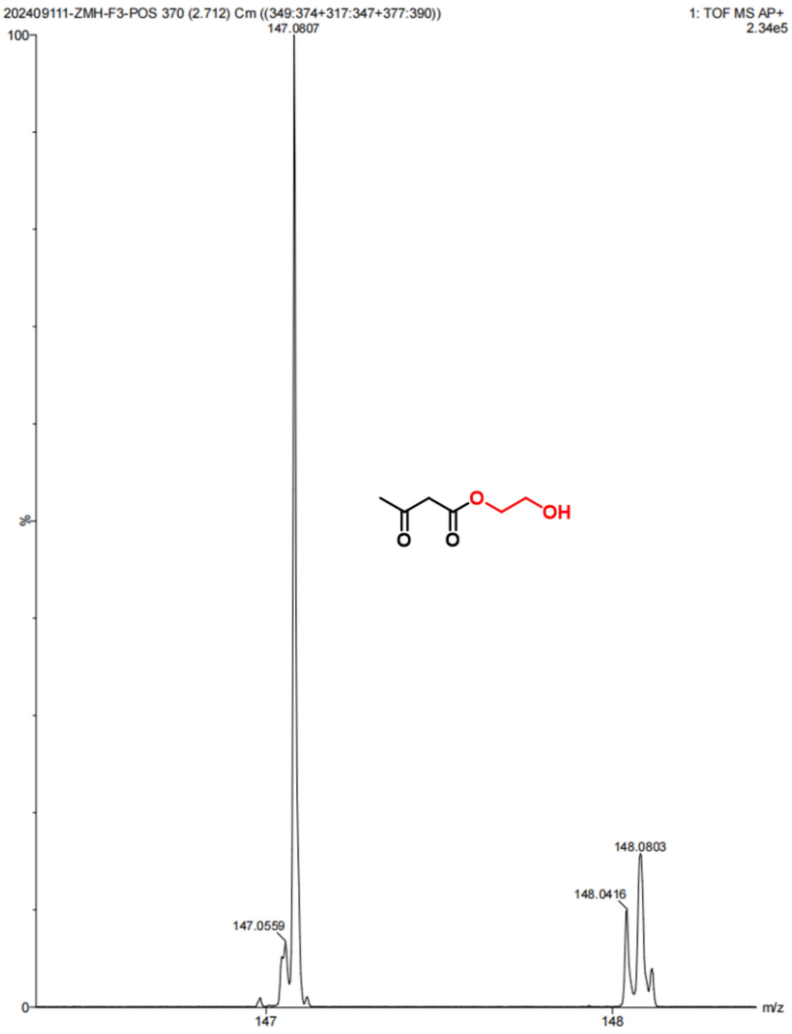

Products derived from EG using methyl trifluoroacetate as a transesterification reagent

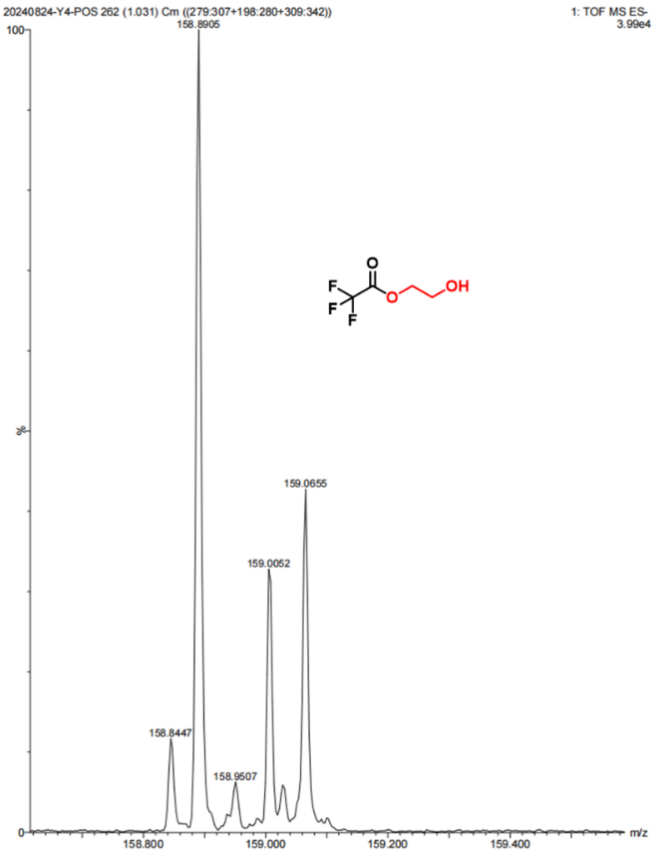

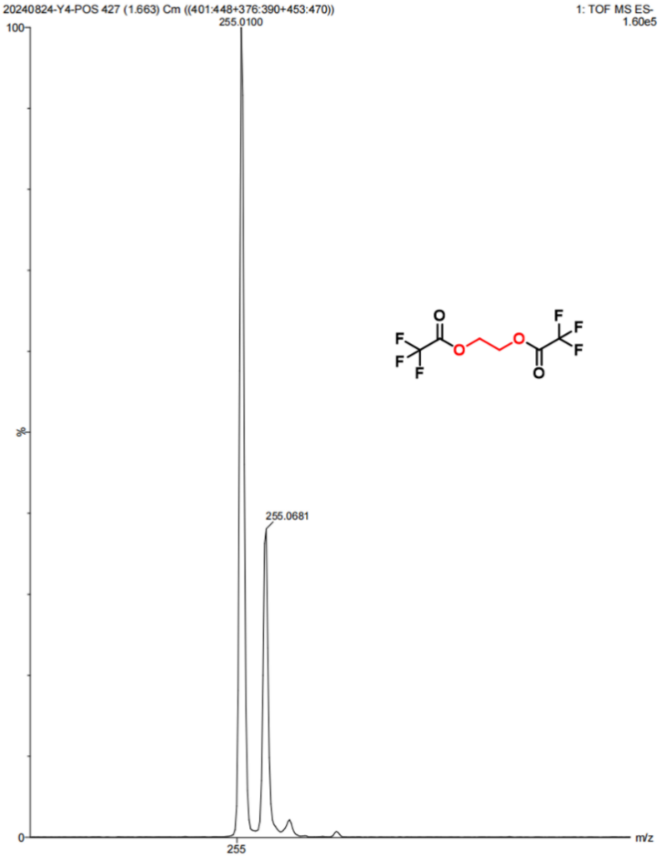

Products derived from EG using methyl acetate as a transesterification reagent

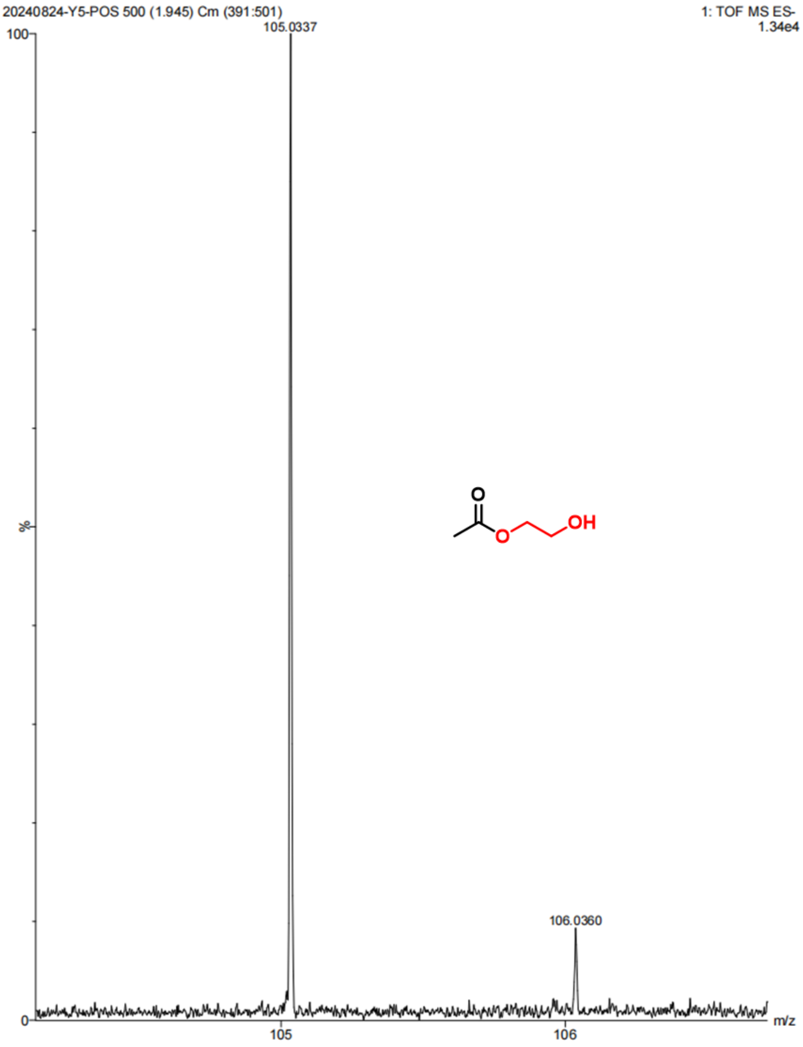

Products derived from EG units using (trimethoxymethyl)benzene as a transesterification reagent

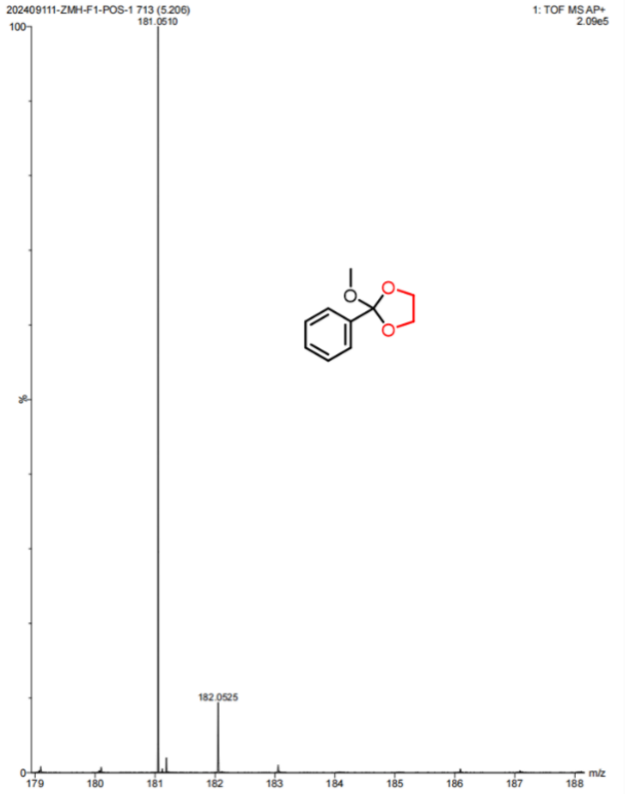

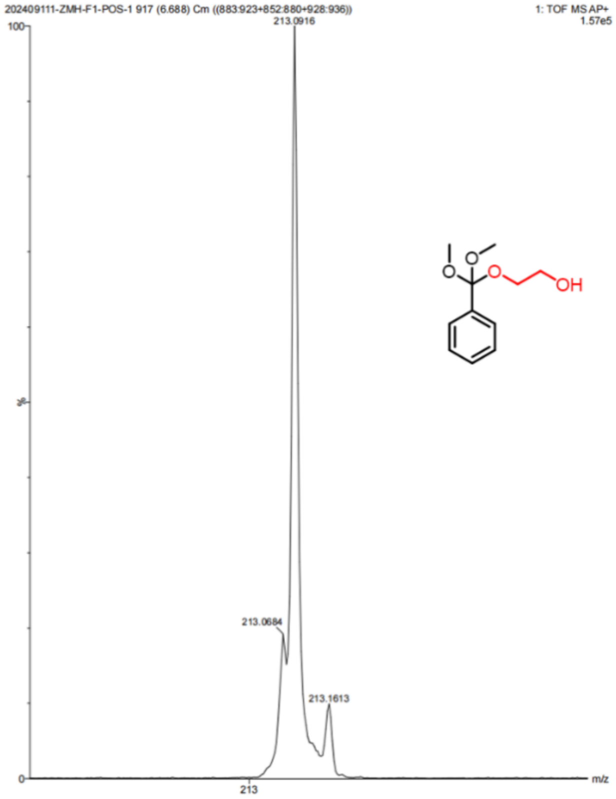

Products derived from EG using titanium methoxide as a transesterification reagent

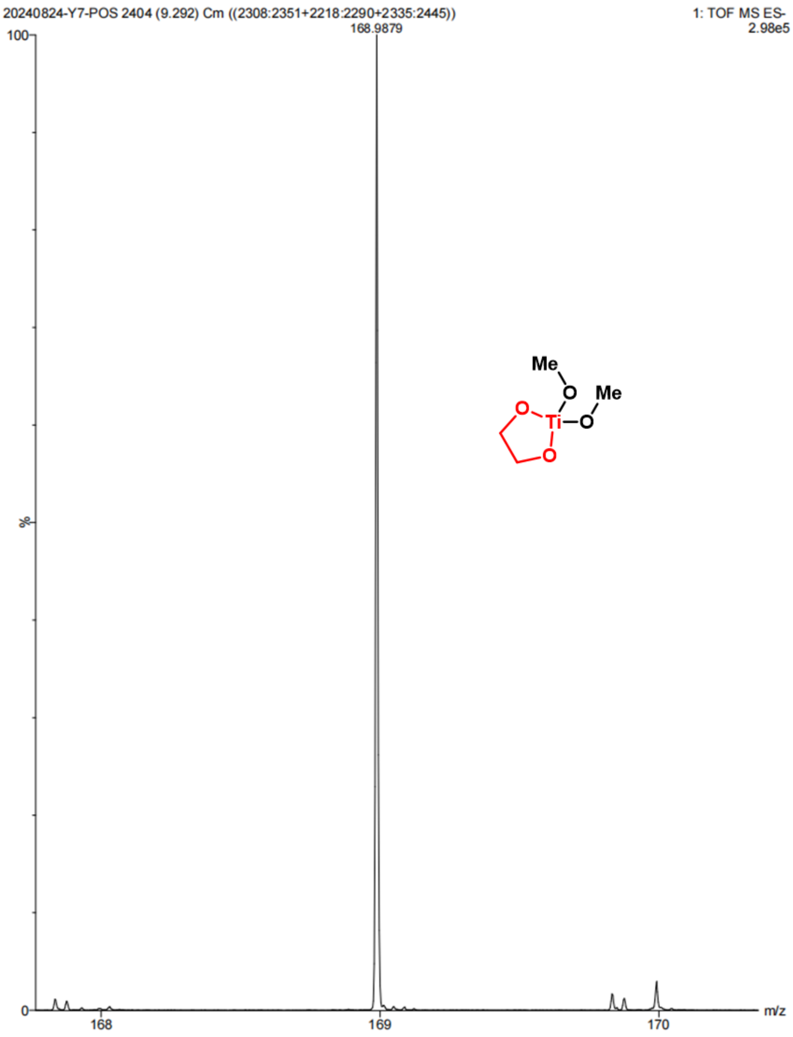

Products derived from EG using tetrabutyl titanate as a transesterification reagent

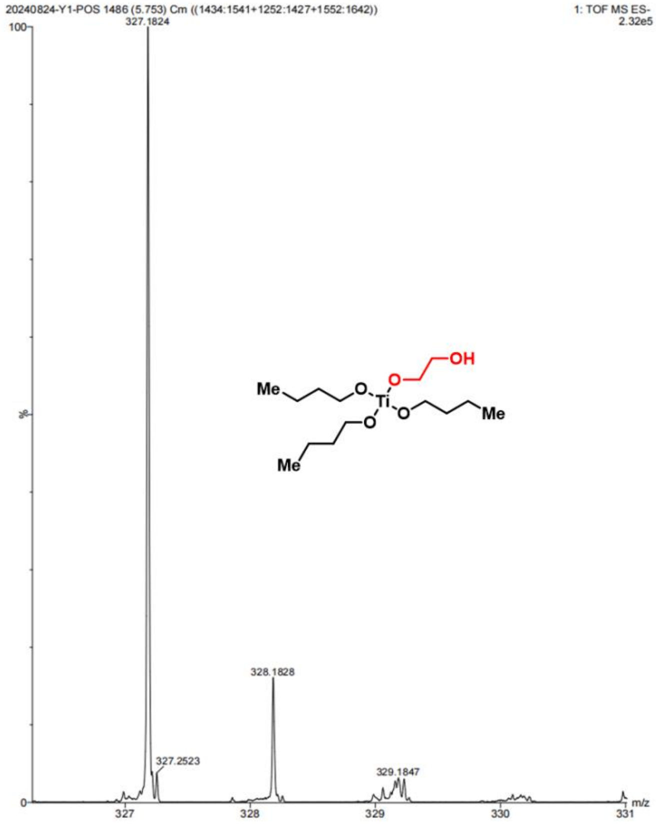

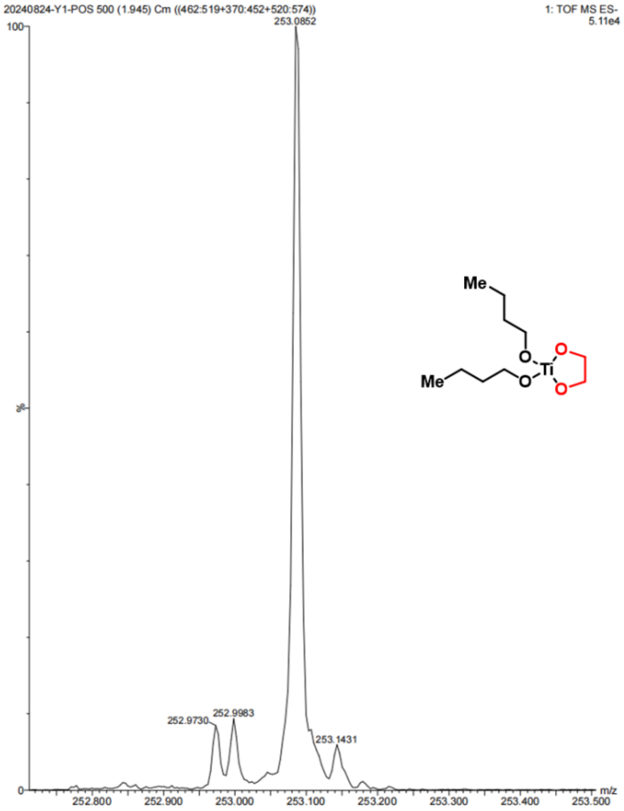

Products derived from EG using tetramethoxysilane as a transesterification reagent

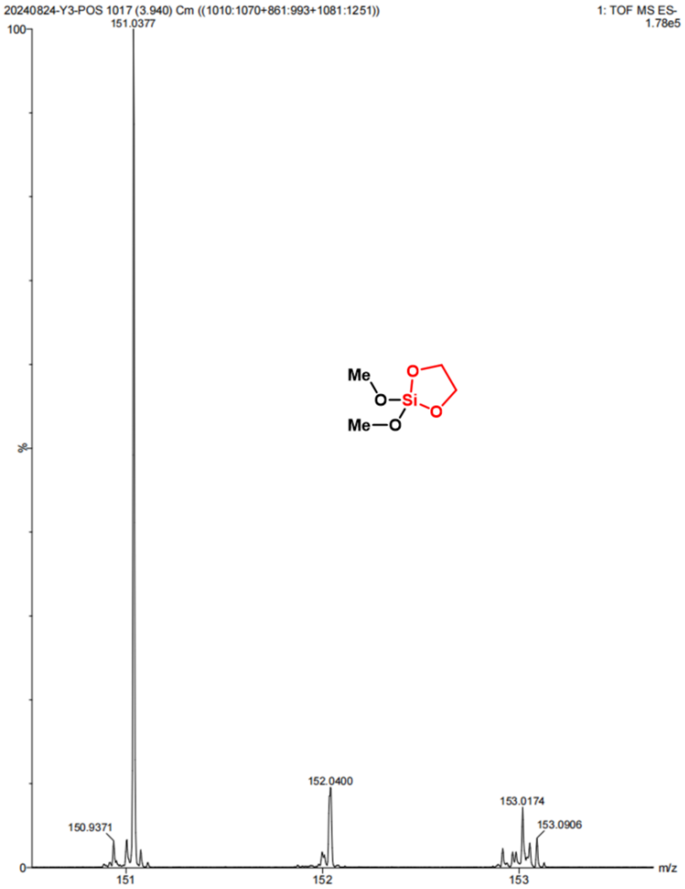

Products derived from EG using tetrabutyl orthosilicate as a transesterification reagent

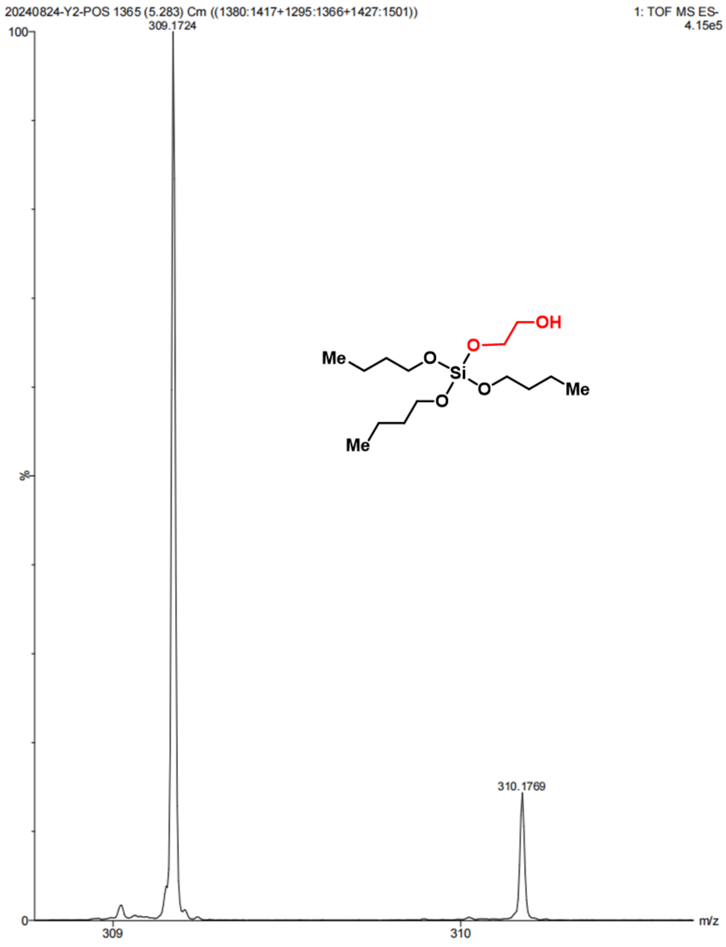

Products derived from EG units using trimethyl phosphate as a transesterification reagent

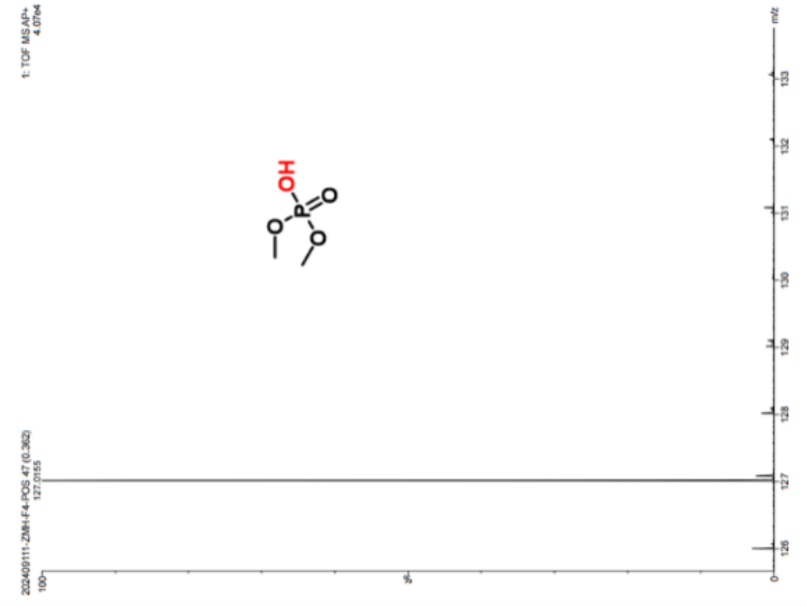

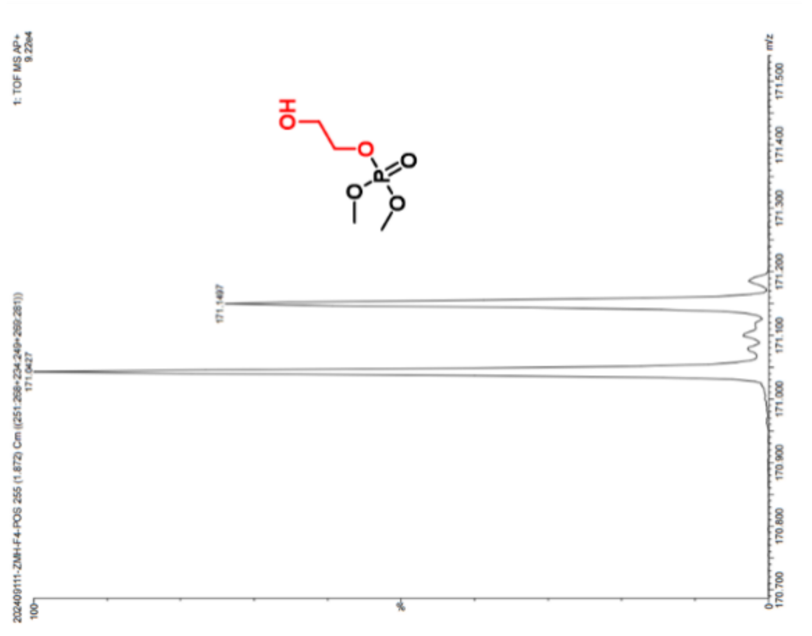

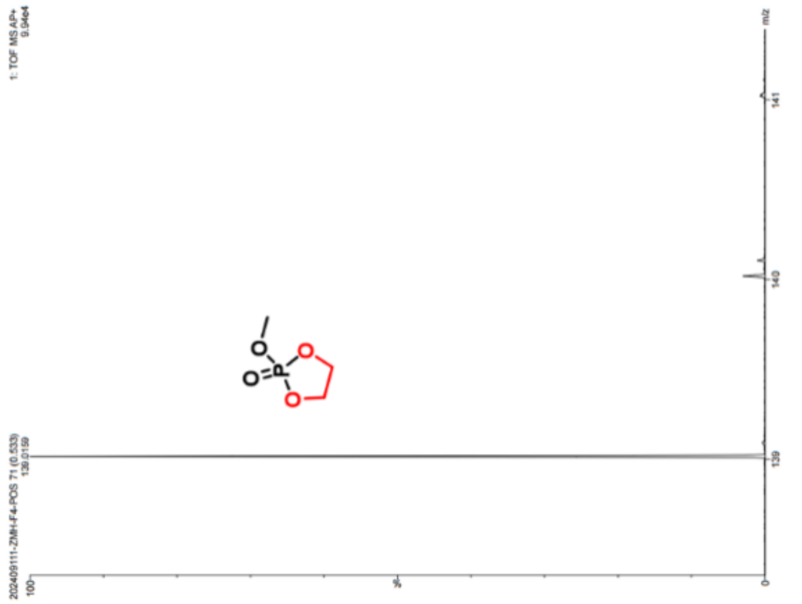

Products derived from EG using triethyl phosphate as a transesterification reagent

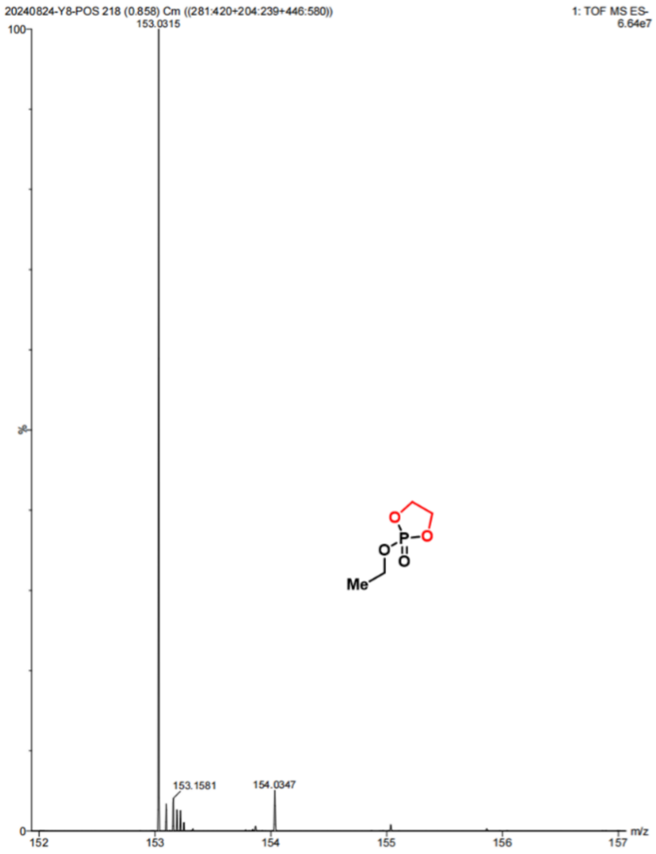

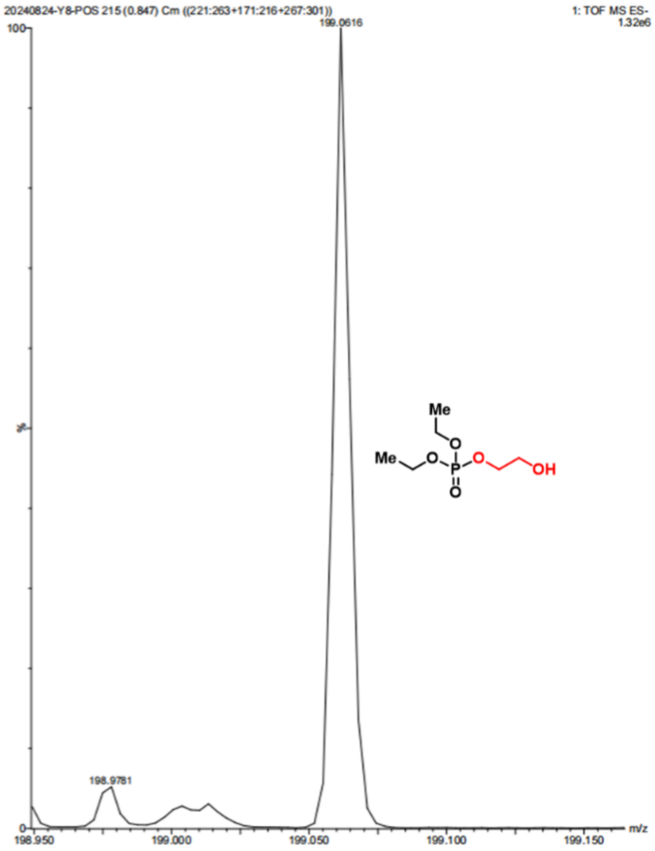

References

1 Shen, Z. Q. et al. CO_2-enhanced PET depolymerization by catalyst free methanolysis. *Process Saf. Environ. Protect.* **188**, 230-238 (2024).

2 Kurokawa, H., Ohshima, M., Sugiyama, K. & Miura, H. Methanolysis of polyethylene terephthalate (PET) in the presence of aluminium tiisopropoxide catalyst to form dimethyl terephthalate and ethylene glycol. *Polym. Degrad. Stabil.* **79**, 529-533 (2003).

3 Mishra, S. & Goje, A. S. Kinetic and thermodynamic study of methanolysis of poly(ethylene terephthalate) waste powder. *Polym. Int.* **52**, 337-342 (2003).

4 Du, J. T. et al. ZnO nanodispersion as pseudohomogeneous catalyst for alcoholysis of polyethylene terephthalate. *Chem. Eng. Sci.* **220**, 115642 (2020).

5 Pham, D. D. & Cho, J. Low-energy catalytic methanolysis of poly(ethyleneterephthalate). *Green Chem.* **23**, 511-525 (2021).

6 Laldinpuii, Z. T. et al. Biomass waste-derived recyclable heterogeneous catalyst for aqueous aldol reaction and depolymerization of PET waste. *New J. Chem.* **45**, 19542-19552 (2021).

7 Lalhmangaihzuala, S., Laldinpuii, Z. T., Khiangte, V., Lallawmzuali, G. & Vanlaldinpuia, K. Orange peel ash coated Fe_3O_4 nanoparticles as a magnetically retrievable catalyst for glycolysis and methanolysis of PET waste. *Adv. Powder Technol.* **34**, 12 (2023).

8 Tanaka, S., Sato, J. & Nakajima, Y. Capturing ethylene glycol with dimethyl carbonate towards depolymerisation of polyethylene terephthalate at ambient temperature. *Green Chem.* **23**, 9412-9416 (2021).

9 Laldinpuii, Z. T. et al. Methanolysis of PET waste using heterogeneous catalyst of bio-waste origin. *J. Polym. Environ.* **30**, 1600-1614 (2022).

10 Ye, B. Y. et al. Upcycling of waste polyethylene terephthalate to dimethyl terephthalate over solid acids under mild conditions. *Green Chem.* **25**, 7243-7252 (2023).

11 Tang, S. X. et al. Calcined sodium silicate as solid base catalyst for alcoholysis of poly(ethylene terephthalate). *J. Chem. Technol. Biotechnol.* **97**, 1305-1314 (2022).

12 Tang, S. X. et al. MgO/NaY as modified mesoporous catalyst for methanolysis of polyethylene terephthalate wastes. *J. Environ. Chem. Eng.* **10**, 107927 (2022).

13 Tang, J. et al. Mechanistic insights of cosolvent efficient enhancement of PET methanol alcohololysis. *Ind. Eng. Chem. Res.* **62**, 4917-4927 (2023).

14 Liu, M. S., Guo, J., Gu, Y. Q., Gao, J. & Liu, F. S. Versatile Imidazole-anion-derived ionic liquids with unparalleled activity for alcoholysis of polyester wastes under mild and green conditions. *ACS Sustain. Chem. Eng.* **6**, 15127-15134 (2018).

15 Jiang, Z. Q. et al. Poly(ionic liquid)s as efficient and recyclable catalysts for methanolysis of PET. *Polym. Degrad. Stabil.* **199**, 109905 (2022).

16 Ma, M. Y. et al. Insights into the depolymerization of polyethylene terephthalate in methanol. *J. Appl. Polym. Sci.* **139**, e52814 (2022).

17 Li, J. B. et al. Efficient methanolysis of PET catalyzed by nonmetallic deep eutectic solvents. *Ind. Eng. Chem. Res.* **63**, 12373-12384 (2024).

18 Qu, X. L. et al. Synergistic catalysis of imidazole acetate ionic liquids for the methanolysis of spiral poly(ethylene 2,5-furandicarboxylate) under a mild condition. *Green Chem.* **23**, 1871-1882 (2021).

19 Hunt, P. A., Ashworth, C. R. & Matthews, R. P. Hydrogen bonding in ionic liquids. *Chem. Soc. Rev.* **44**, 1257-1288 (2015).

20 Liu, Y. C. et al. Degradation of poly(ethylene terephthalate) catalyzed by metal-free choline-based ionic liquids. *Green Chem.* **22**, 3122-3131 (2020).

21 Yu, X. X., Wang, M. M. & Huang, X. R. Spectroscopy and kinetics evidence for the hydrogen-bond activating effect of anion/cation of [Bmim]OAc on the hydrolysis of esters. *J. Mol. Liq.* **216**, 354-359 (2016).

22 Schick, D. et al. Influence of solvents and salts on CO_2 solubility and the impact on an esterification reaction. *J. Chem. Eng. Data* **69**, 560-572 (2023).

23 Pabsch, D. et al. Influence of solvent and salt on kinetics and equilibrium of esterification reactions. *Chem. Eng. Sci.* **263**, 118046 (2022).

24 Adamson, A. et al. Improving lithium-ion cells by replacing polyethylene terephthalate jellyroll tape. *Nat. Mater.* **22**, 1380-1386 (2023).

25 Marciniak, A. A., Alves, O. C., Appel, L. G. & Mota, C. J. A. Synthesis of dimethyl carbonate from CO_2 and methanol over CeO_2: Role of copper as dopant and the use of methyl trichloroacetate as dehydrating agent. *J. Catal.* **371**, 88-95 (2019).

26 Wang, Q. et al. Urea as an efficient and reusable catalyst for the glycolysis of poly(ethylene terephthalate) wastes and the role of hydrogen bond in this process. *Green Chem.* **14**, 2559-2566 (2012).

27 Liu, M. S. et al. Pushing the limits in alcoholysis of waste polycarbonate with DBU-based ionic liquids under metal- and solvent-free conditions. *ACS Sustain. Chem. Eng.* **6**, 13114-13121 (2018).

28 Liu, M. Y. et al. Transformation of alcohols to esters promoted by hydrogen bonds using oxygen as the oxidant under metal-free conditions. *Sci. Adv.* **4**, eaas9319 (2018).

29 Ji, Y., Sweeney, J., Zoglio, J. & Gorin, D. J. Catalytic methyl transfer from dimethylcarbonate to carboxylic acids. *J. Org. Chem.* **78**, 11606-11611 (2013).

30 Wang, X., Wang, Z. H., Ishida, T. & Nishihara, Y. Methoxylation of acyl fluorides with tris(2,4,6-trimethoxyphenyl)phosphine via C-OMe bond cleavage under metal-free conditions. *J. Org. Chem.* **85**, 7526-7533 (2020).

31 Zhu, Y. et al. Copper-catalyzed methyl esterification reactions via C-C bond cleavage. *J. Org. Chem.* **78**, 9898-9905 (2013).

32 Guo, Y. F. et al. Oxidation of aromatic aldehydes to esters: a sulfate radical redox system. *J. Org. Chem.* **82**, 1591-1599 (2017).

33 Yu, C. J., Özkaya, B. & Patureau, F. W. Electro-oxidative selective esterification of methylarenes and benzaldehydes. *Chem.-Eur. J.* **27**, 3682-3687 (2021).

34 Lee, M., Hwang, Y. K. & Kwak, J. Ag(I)-catalyzed C-H carboxylation of thiophene derivatives. *Organometallics* **40**, 3136-3144 (2021).

35 Yasukawa, T., Yang, X. & Kobayashi, S. Development of N-doped carbon-supported cobalt/copper bimetallic nanoparticle catalysts for aerobic oxidative esterifications based on polymer incarceration methods. *Org. Lett.* **20**, 5172-5176 (2018).

36 Takaishi, K., Iwachido, K., Takehana, R., Uchiyama, M. & Ema, T. Evolving fluorophores into circularly polarized luminophores with a chiral naphthalene tetramer: proposal of excimer chirality rule for circularly polarized luminescence. *J. Am. Chem. Soc.* **141**, 6185-6190 (2019).
